# Adipokinome Signatures in Obese Mouse Models Reflect Adipose Tissue Health and Are Associated with Serum Lipid Composition

**DOI:** 10.3390/ijms20102559

**Published:** 2019-05-24

**Authors:** Birgit Knebel, Pia Fahlbusch, Gereon Poschmann, Matthias Dille, Natalie Wahlers, Kai Stühler, Sonja Hartwig, Stefan Lehr, Martina Schiller, Sylvia Jacob, Ulrike Kettel, Dirk Müller-Wieland, Jörg Kotzka

**Affiliations:** 1 Institute of Clinical Biochemistry and Pathobiochemistry, German Diabetes Center at the Heinrich-Heine-University Duesseldorf, Leibniz Center for Diabetes Research; 40225 Duesseldorf, Germany; birgit.knebel@ddz.de (B.K.); pia.fahlbusch@ddz.de (P.F.); matthias.dille@ddz.de (M.D.); nawah101@hhu.de (N.W.); sonja.hartwig@ddz.de (S.H.); stefan.lehr@ddz.de (S.L.); martina.schiller@ddz.de (M.S.); sylvia.jacob@ddz.de (S.J.); ulrike.kettel@ddz.de (U.K.); 2 German Center for Diabetes Research (DZD), Partner Duesseldorf, 40225 Duesseldorf, Germany; 3 Institute for Molecular Medicine, University Hospital Duesseldorf, Heinrich Heine University Duesseldorf, 40225 Duesseldorf, Germany; gereon.poschmann@hhu.de (G.P.); kai.stuehler@hhu.de (K.S.); 4 Heinrich-Heine-University Duesseldorf, Molecular Proteomics Laboratory, BMFZ, 40225 Duesseldorf, Germany; 5 Clinical Research Centre, Department of Internal Medicine I, University Hospital Aachen, 52074 Aachen, Germany; dirmueller@ukaachen.de

**Keywords:** Nonalcoholic fatty liver disease, fatty liver, free fatty acids, label-free proteomic profiling, adipokine, obesity, visceral fat, sick fat

## Abstract

Adipocyte and hepatic lipid metabolism govern whole-body metabolic homeostasis, whereas a disbalance of de novo lipogenesis (DNL) in fat and liver might lead to obesity, with severe co-morbidities. Nevertheless, some obese people are metabolically healthy, but the “protective” mechanisms are not yet known in detail. Especially, the adipocyte-derived molecular mediators that indicate adipose functionality are poorly understood. We studied transgenic mice (alb-SREBP-1c) with a “healthy” obese phenotype, and obob mice with hyperphagia-induced “sick” obesity to analyze the impact of the tissue-specific DNL on the secreted proteins, i.e., the adipokinome, of the primary adipose cells by label-free proteomics. Compared to the control mice, adipose DNL is reduced in both obese mouse models. In contrast, the hepatic DNL is reduced in obob but elevated in alb-SREBP-1c mice. To investigate the relationship between lipid metabolism and adipokinomes, we formulated the “liver-to-adipose-tissue DNL” ratio. Knowledge-based analyses of these results revealed adipocyte functionality with proteins, which was involved in tissue remodeling or metabolism in the alb-SREBP-1c mice and in the control mice, but mainly in fibrosis in the obob mice. The adipokinome in “healthy” obesity is similar to that in a normal condition, but it differs from that in “sick” obesity, whereas the serum lipid patterns reflect the “liver-to-adipose-tissue DNL” ratio and are associated with the adipokinome signature.

## 1. Introduction

Obesity is a worldwide health burden. Obesity is prone to severe co-morbidities, including diabetes, cardiovascular disease, and lipotoxicity due to ectopic lipid accumulation. However, some obese individuals do not suffer from obesity-associated syndromes, which is the so-called phenomenon of “fit and fat”. This was due to a healthy metabolism and insulin-sensitive adipose tissue, which might increase the likelihood of the incidence of a fatty liver. In contrast, “sick fat” people show an elevated lipid load, inflammation, hyperplasia, insufficient vascularization, and fibrosis of adipose tissue [1,2,3]. Unfortunately, the “point of no return” from healthy obese people, with functional adipose tissue, to unhealthy obesity, prone to comorbidities, still remains unknown.

There is a close interaction between adipocyte and hepatic lipid metabolism. Adipocytes are essential in whole-body energy homeostasis for the storage of dietary lipids and of lipids generated by de novo lipogenesis (DNL) from alimentary carbohydrates in adipose tissue or the liver. While it is an elementary process for survival, excessive hepatic lipogenesis is a key feature of many models of obesity and diabetes. Therefore, hepatic DNL is thought to be a health burden, as it correlates with ectopic hepatic lipid accumulation and insulin resistance [4,5,6]. One key regulator of hepatic DNL is the transcription factor sterol regulatory element-binding protein (SREBP)-1. SREBP-1c activity persists even in insulin resistant states, as seen in obesity and T2D. In this context, we have shown that the hepatic overexpression of the transcription active domain of SREBP-1c increases hepatic DNL, without severe insulin resistance, resulting in a fatty liver and a massively increased adipose tissue mass in mouse models [7,8].

In contrast to the liver, adipose tissue stores lipids from blood circulation, without the need for synthesis, and only a marginal amount of lipids is produced by adipose tissue DNL [9]. Therefore, it is unlikely that the role of adipose tissue DNL is primary for lipid storage, but it may act solely for signaling or regulation processes, initiated by the physiological status of the adipose tissue.

It is well accepted that adipose tissue secretes endocrine- and exocrine-acting proteins, i.e., adipokines or adipokinome. These secreted proteins influence food intake, energy metabolism, and insulin sensitivity [10,11,12]. The alteration of adipokinome in regard to metabolic conditions or diseases has been shown to have an impact on fat mass, either by affecting adipocyte hyperplasia or hypertrophy [13,14,15,16]. We recently showed that adipokinomes are correlated with clinical parameters in diabetes [17]. Thus, adipokinome changes in relation to the lipid composition of the adipose tissue and reflects the overall physiological condition of the adipose tissue. Furthermore, there are hints that certain adipokines interfere with the mechanism of hepatic fibrosis [10,11,12,17].

It is therefore tempting to raise the hypothesis that adipokinome is also involved in the adipose tissue-to-liver interaction for energy homeostasis regulation.

In the present study, we compare the adipokinomes of mice with increased hepatic DNL by the genetic overexpression of the N-terminal domain of SREBP-1c [7], as a model for “healthy” obesity, with hyperphagia-induced morbid obese mice (obob), as model for “sick” adipose tissue or lean mice (C57Bl6) by label-free proteomics.

## 2. Results

### 2.1. Physiological Characterization of the Mouse Models

SREBP-1c and obob mice had a marginally higher body weight, fat mass, and liver weight than C57Bl6 animals (Table 1). Blood glucose (BG) and triglycerides (TG) were also increased in the obese mouse models, and additionally, cholesterol was increased in obob mice. Food consumption was comparable in C57Bl6 and alb-SREBP-1c mice and increased in obob animals. Interestingly, the weight gain per unit of food consumed was increased 1.5- to 2-fold in obese mice. Liver enzymes ALT, AST, and GLDH indicated gradual hepatic impairment in the obese mouse models. Overall, alb-SREBP-1c mice were intermediate to obob and C57Bl6 mice. Relevant metabolic hormones showed higher levels of insulin in the obese mice. Leptin was higher in alb-SREBP-1c, compared to C57Bl6, and a leptin deficiency was confirmed in obob. Surrogate parameters for insulin resistance (HOMA-IR) and insulin secretion (HOMA-β%) confirmed a mild insulin resistance (IR) in alb-SREBP-1c and a strong IR in obob mice.

### 2.2. Lipid Composition of Serum, Liver, and Adipose Tissue

**Figure 1:**
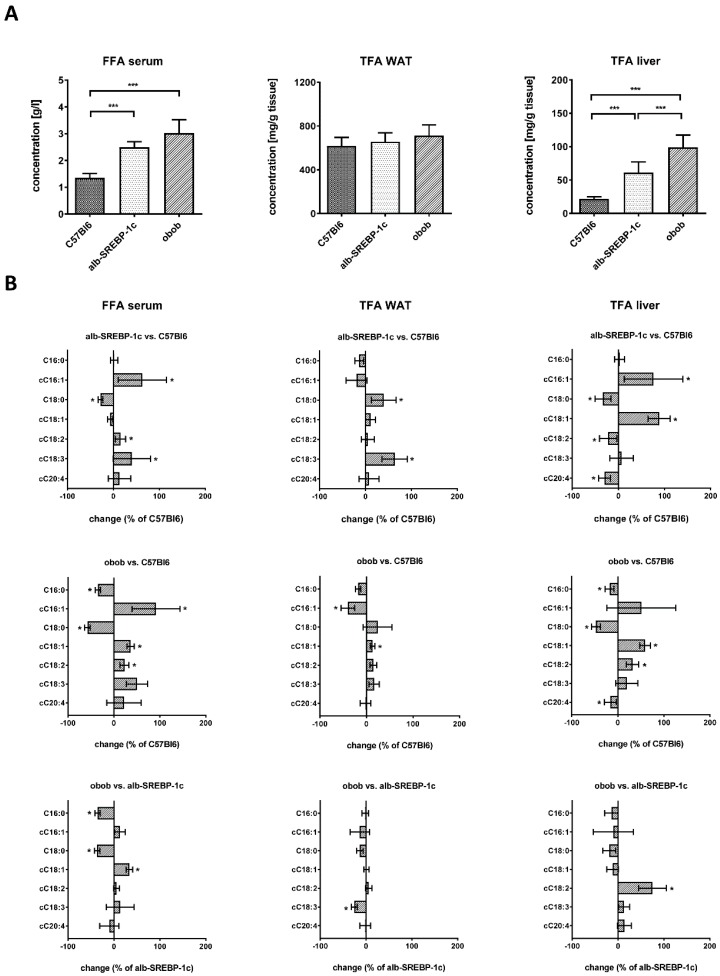
Lipid compositions of the C57Bl6, alb-SREBP-1c, and obob mice. (**A**) Total and specific fractional composition of the serum free fatty acids (FFAs) and the liver or adipose tissue total fatty acids (TFAs); (**B**) the percentage changes in the serum FFA and the liver or adipose tissue TFAs in alb-SREBP-1c vs. C57Bl6, obob vs. C57Bl6, and obob vs. alb-SREBP-1c. Data are expressed as mean ± SD (*n* = 8 of each phenotype). * *p* < 0.05, ** *p* < 0.01, and *** *p* < 0.001, by one-way ANOVA, with a Sidak post-hoc test (A) or students’ *t*-test (B).

Serum-free fatty acid (FFA) was increased more than 2-fold in alb-SREBP-1c mice and more than 3-fold in obob mice. However, the total fatty acid (TFA) content of adipose tissue was similar to C57Bl6 mice, independent of the cause of obesity. In contrast, hepatic TFA was increased to a similar degree as free fatty acid in serum (Figure 1A). The change in the lipid composition in serum revealed increased cC16:1 and decreased C18:0 in obese models, compared to controls (Figure 1B). Both obese models differ in C16:0 and C18:0 and show inverse levels for the FFA cC18:1. FFA cC18:2, cC18:3, and cC20:4 were comparably altered. In adipose tissue, lipid composition, compared to controls, indicated, in alb-SREBP-1c mice, an increase in C18:0 and cC18:3 and, in obob mice, a decrease in cC16:1 and an increase in cC18:1. The obesity models differed only in the essential FA cC18:3 (Figure 1B). In the liver, the obese models mainly differed in an increase in cC16:1 and cC18:1 and a reduction of C18:0 and cC20:4, compared to the control mice, whereas the essential FA cC18:2 was more pronounced in the obob mice (Figure 1B).

In further detail, the percentage of serum lipid classes is shown in Figure S1A. The grouping of lipids showed that the amounts of saturated FA (SFA) were decreased, while the unsaturated FA (UFA), mono-UFA (MUFA), poly-UFA (PUFA), or essential FA (EFA) were increased, in the serum of obese mice (Figure S1B). The percentage of adipose tissue lipid classes is shown in Figure S2A. Here, only SFA was changed in both obese mice, compared to the controls, whereas UFA, PUFA, and EFA differed only in the obob mice (Figure S2B). The percentage of liver lipid classes is shown in Figure S3A. In the liver, a decreased SFA and increased UFA and MUFA were present in the obesity models, the latter being the highest in the alb-SREBP-1c mice. Compared to the controls, the content of EFA was higher in the obob and lower in the alb-SREBP-1c mice, and vice versa, for the non-essential FA (NEFA) (Figure S3B).

Lipid indices and surrogate parameters for the enzyme activity in lipid metabolism were calculated based on the lipid compositions determined in the tissues. In WAT (Figure S4A), the Δ6 desaturase activity was reduced in the alb-SREBP-1c mice. However, the elongase activity was elevated in both obese mice. In the liver (Figure S4B), the Δ9 desaturase activity for C16:0 as well as C18:0 was elevated in obese mice. The Δ6 desaturase activity was comparable in the C57Bl6 and obob mice, but lower in the alb-SREBP-1c mice, whereas the Δ5 desaturase activity was comparable in the C57Bl6 and alb-SREBP-1c and higher in the obob mice. Furthermore, a lower elongase activity, compared to controls, was observed in both obese mice.

### 2.3. Adipose Tissue DNL versus Hepatic DNL

**Figure 2:**
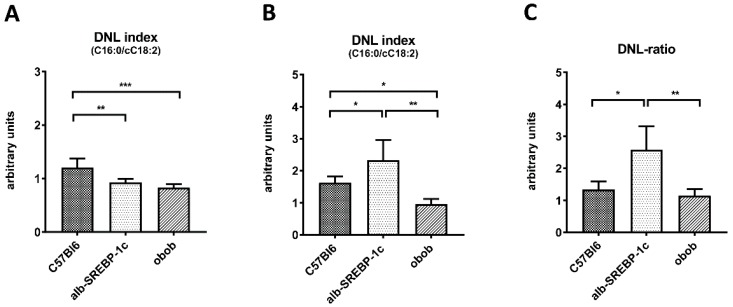
Tissue-specific de novo lipogenesis (DNL). (**A**) The DNL index (C16:0/cC18:2) of adipose tissue; (**B**) DNL index (C16:0/cC18:2) of the liver; and (**C**) “liver-to-adipose-tissue DNL” ratio. Data are expressed as mean ± SD (*n* = 8 of each phenotype). * *p* < 0.05, ** *p* < 0.01, and *** *p* < 0.001, by one-way ANOVA, with a Sidak post-hoc test.

In adipose tissue, DNL declined according to the degree of obesity and insulin resistance status from C57Bl6 (1.21 ± 0.18) to alb-SREBP-1c (0.93 ± 0.07) and obob mice (0.83 ± 0.07) (Figure 2A). Analyses of the hepatic DNL showed that it was higher in alb-SREBP-1c (2.33 ± 0.55) but lower in obob mice (0.96 ± 0.17), compared to the controls (1.62 ± 0.17) (Figure 2B). In regard to the serum lipid levels of cC16:1 and cC18:1, solely a correlation to the adipose tissue DNL in alb-SREBP-1c mice (cC16:1 to DNL: R = 0.940, P = 0.017; and cC18:1 to DNL: R = 0.941, P = 0.017), but neither in the lean control nor in the obob mice, was observed. (Table S1). A direct comparison of the adipocyte and hepatic DNL indicated that alb-SREBP-1c mice have the largest difference in “liver-to-adipose-tissue” DNL ratio (2.58 ± 0.67), whereas obob mice have a ratio of 1.14 ± 0.23, which is a comparable DNL-ratio to C57Bl6 mice (1.34 ± 0.3) (Figure 2C). 

### 2.4. Adipokinome

The secretome of the isolated adipocytes from visceral fat depots of the mouse models, analyzed by electron spray MS, identified 922 unique proteins. Of all of the proteins, 543 (59%) were predicted as classical or non-classical secreted proteins (SP+/SP−). The remaining 379 proteins identified were not predicted to be secreted (NP) (Table S2).

**Figure 3:**
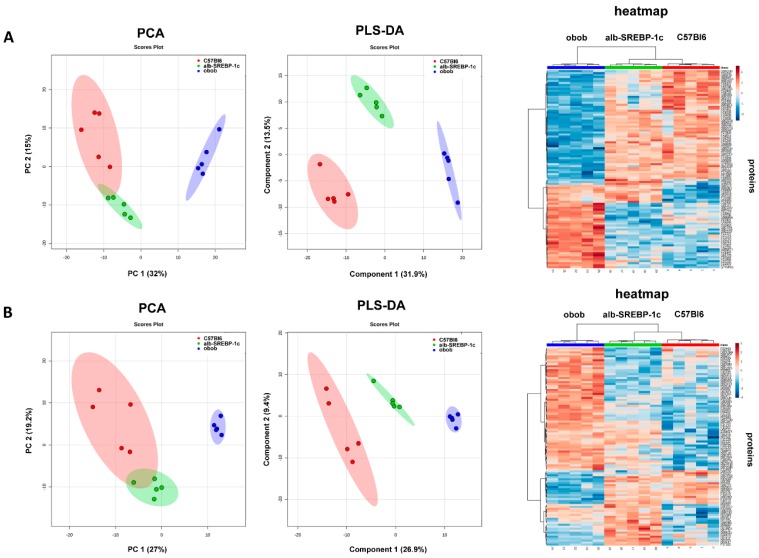
Classification of the mouse models according to the identified differential adipokinomes: (**A**) classically or non-classically secreted (SP+/SP−) proteins and (**B**) NP proteins. Principal component analyses (PCA), partial least square discriminant analyses (PLS-DA), and a heat map of the top 100 proteins with the greatest difference (ANOVA).

The intensity patterns of the proteins were predicted to be either classically or non-classically secreted (SP+/SP−) or not predicted to be secreted (NP), and the various mouse models were distinguished in PCA analyses (Figure 3A). Component 1 of the PCA accounted for 30% of the total variance and clearly separated obob from lean mice. The separation of C57Bl6 and alb-SREBP-1c mice was not so clearly achieved in these analyses, as the 95% confidence levels overlapped. Nevertheless, PLS-DA analyses (Figure 3B) and unsupervised cluster analyses segregated mouse models according to phenotype, indicating specific differences in the adipokines patterns.

### 2.5. Differential Adipokinomes

Overall, 60% of the SP+/SP− and NP proteins were differentially abundant in the comparisons between C57Bl6 vs. alb-SREBP-1c, C57Bl6 vs. obob, and alb-SREBP-1c vs. obob (Table S3). In the comparison of alb-SREBP-1c and lean C57Bl6 mice secretomes, 121 proteins were identified (70 SP+/SP−; 51 NP) with significantly different secretions. SP+/SP− proteins mainly point to cell cycle modification, including cellular component organization or biogenesis (adjP = 4.67 × 10^−5^), actin cytoskeleton organization (adjP = 0.0006), or actin remodeling (adjP = 0.001). Further alterations can solely be annotated to metabolic GO category superfamilies, e.g., cellular process (adjP = 0.0002), but are not specified in more detail. NP adipokines also annotate to cellular modifications, e.g., cellular component organization or biogenesis (adjP = 3.93 × 10^−7^), macromolecule localization, (adjP = 8.79 × 10^−7^) or inhibitory signaling processes, e.g., inhibitor activity to phospholipase A2 (adjP = 0.0003) or lipase (adjP = 0.0016), with a moderate stringency (Table S4).

The overall comparison C57Bl6 vs. obob identified 376 differentially abundant proteins (235 SP+/SP−; 141 NP). SP+/SP−proteins were involved solely in metabolic pathways, like the preamble cellular process (adjP = 1.22 × 10^−13^), and a vast amount of detailed metabolic relevant annotations, including metabolic process (adjP = 7.87 × 10^−17^), organic acid metabolic process (adjP = 3.64 × 10^−13^), or NAD binding (adjP = 2.63 × 10^−8^). Of 141 differentially abundant NP proteins, metabolic processes, e.g., pyridoxal phosphate-binding adjP = 1.33 × 10^−5^ and the carboxylic acid metabolic process adjP = 6.78 × 10^−8^, as well as cell modifying functions, e.g., the protein complex adjP = 8.75 × 10^−13^, cellular component biogenesis adjP = 1.00 × 10^−6^, or actin-binding adjP = 1.33 × 10^−5^, are equally present (Table S4).

A direct comparison of alb-SREBP−1c vs. obob adipocyte secretomes identified 396 different adipokines (156 SP+/SP−; 150 NP). SP+/SP− adipokines were exclusively annotated to metabolic processes (adjP = 4.47 × 10^−26^), including, e.g., organic acid metabolic process (adjP = 1.44 × 10^−27^) or NAD binding (adjP = 1.56 × 10^−12^). NP adipokines also solely annotate to metabolic processes, e.g., the metabolic carboxylic acid metabolic process (adjP = 1.27 × 10^−15^) and oxoacid metabolic process (adjP = 4.68 × 10^−15^), or energy-producing organelles (mitochondrion; adjP = 5.10 × 10^−16^) (Table S4).

### 2.6. Knowledge-Based Analyses

**Figure 4:**
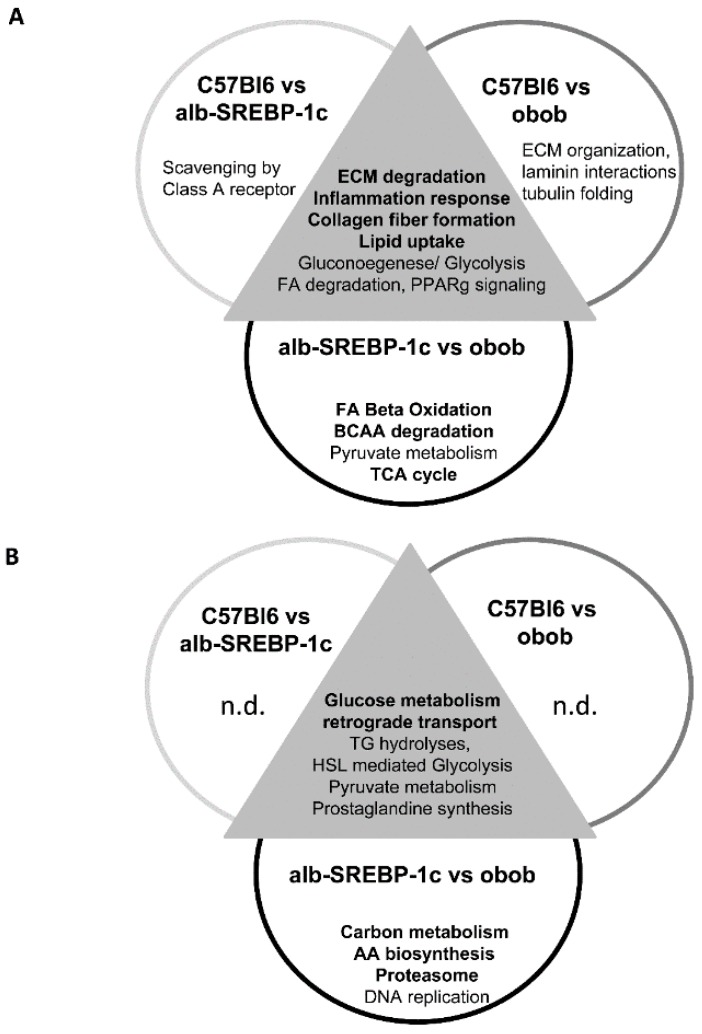
Unique pathways activated by differentially secreted adipokines. The prediction of secretory protein classification as classical SP+/SP− signaling sequences or non-classically secreted proteins was performed with SignalP 4.1 or SecretomeP 2.0, as indicated in the methods section. The functional annotation of differentially abundant proteins was performed separately on (**A**) proteins with classical SP+/SP− signaling sequences and (**B**) non-classically secreted proteins. Abbreviations: AA: amino acids, BCAA: branched chain amino acids; ECM: extracellular matrix; FA: fatty acids; n.d.: not detected, TCA cycle: tricarboxylic acid cycle.

Venn analyses revealed comparisons of C57Bl6 and either alb-SREBP-1c or obob, as well as alb-SREBP-1c vs. obob bare overlapping, but also specific different adipokine patterns of SP+/SP− and NP proteins (Figure 4, complete analyses in Table S5).

Knowledge-based analyses of protein interactions indicated the common and individual specificity of all three different adipokinomes. The common pathways affected by secreted SP+/SP− proteins are involved in ECM modeling, inflammation, lipid uptake, and gluconeogenesis or FA degradation. In NP proteins, the transport and glucose metabolism proteins were common, while in SP+/SP− proteins, C57Bl6 and alb-SREBP-1c differed in the proteins involved in class A receptor signaling, and proteins involved in the formation of extracellular matrix proteins dominated in C57Bl6 vs. obob.

No specific pathway can be identified in the NP proteins, compared to the controls. The comparison of both obese models focusses on proteins involved in central metabolic pathways, with a major participation of lipid metabolic processes in SP+/SP− proteins and the proteasome complex formation in NP (Figure 4).

**Figure 5:**
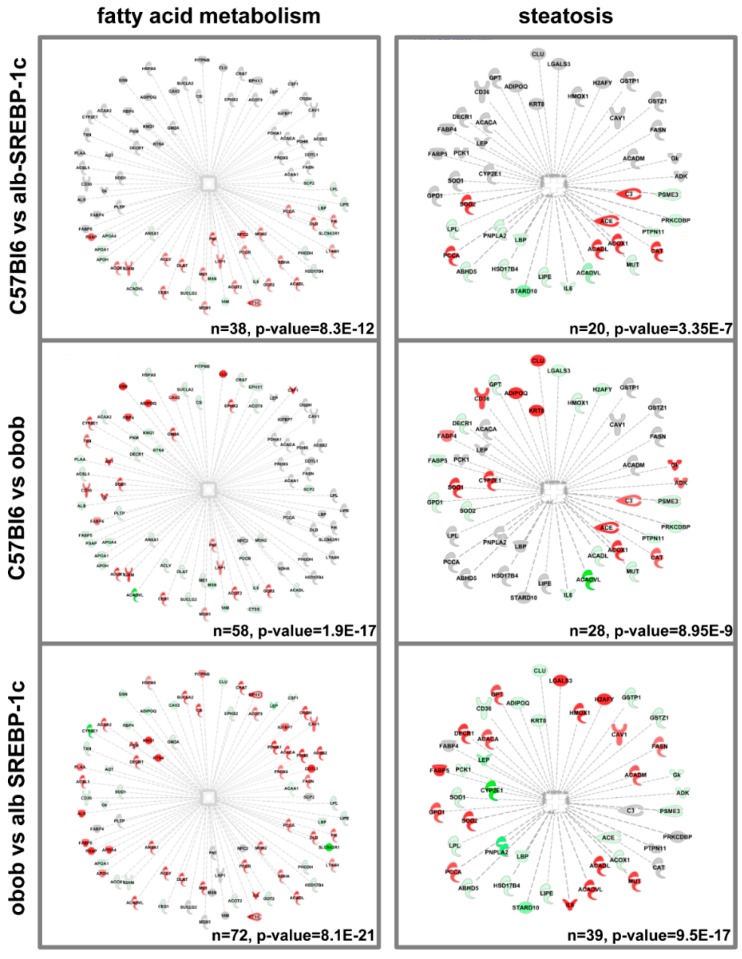
Differential enrichment of FA metabolism and steatosis-related proteins in the adipokinomes. The annotation of specific differentially abundant proteins to lipid metabolism or steatosis is shown. Data were analyzed with IPA^®^ core analyses (default settings). Coloring represents proteins with different abundances in the comparisons C57Bl6 vs. alb-SREBP-1c, C57Bl6 vs. obob, and alb-SREBP-1c vs. obob. (Red: more abundant; green: less abundant; grey: not regulated in the specific comparison).

To determine more informative interactions of the different adipokinomes, we used integrative annotation to extend the information to the suggested up- or downstream interactions of the identified proteins. Within these analyses, the most prominent functional overlap was the differential protein patterns per genotype, set to fatty acid metabolism or proteins initially identified to be related to steatosis (Figure 5). There was a differential overlap with fatty acid metabolism in the protein sets of C57Bl6 vs. alb-SREBP-1c (*n* = 38, *p*-value = 8.3 × 10^−12^), C57Bl6 vs. obob (*n* = 58, *p*-value = 1.9 × 10^−17^), and C57Bl6 vs. alb-SREBP-1c (*n* = 72, *p*-value = 8.1 × 10^−21^) (Figure 5). In addition, the number and significance of the proteins related to steatosis processes gradually increased with the severity of obesity in the comparisons of C57Bl6 vs. alb-SREBP-1c (*n* = 20, *p*-value 3.35 × 10^−7^), C57Bl6 vs. obob (*n* = 28, *p*-value 8.9 × 10^−9^), and obob vs. alb-SREBP-1c (*n* = 39, *p*-value 9.5 × 10^−17^) (Figure 5).

### 2.7. Adipokinome—Marker for Tissue-Specific DNL?

To account for the hypothesis that adipokinomes reflected, a marker for adipose tissue functionality and the physiological status of the adipose tissue, we analyzed the different adipokine patterns, identified for correlations, in relation to the specific “liver-to-adipose-tissue DNL” ratio. Of the 922 proteins observed, 55 proteins in C57Bl6 showed a correlation with the “liver-to-adipose-tissue DNL” ratio (Table S1). These included proteins involved in lipid droplet formation, like perilipin, actin-binding or assembly molecules; echinoderm microtubule-associated protein-like 2, plastin-2, -3, or villin molecules; metabolic enzymes, like acyl-CoA dehydrogenase, glycogen phosphorylase, L-lactate dehydrogenase, or NADP(+)-dependent alcohol dehydrogenase; and molecules of the glutathione metabolism involved in ROS clearance.

A total of 50 proteins were correlated with the “liver-to-adipose-tissue DNL” ratio in alb-SREBP-1c and 42 in obob mice. In alb-SREBP-1c mice, more metabolic active molecules were present, e.g., pyruvate kinase; NADPH-cytochrome P450 reductase; fructose-1,6-bisphosphatase; and l-lactate dehydrogenase or the peroxisomal delta(3,5)-delta(2,4)-dienoyl-CoA isomerase (ECH), an auxiliary involved in lipid metabolism. Other proteins were regulatory, like farnesyl pyrophosphate synthase, and protease regulatory subunits were present. In obob mice, the share of relevant correlative metabolic proteins included glycolysis and glyconeogenesis-related proteins, like pyruvate and malat dehydrogenases, and fructose-1,6-bisphosphatase 1, but also proteins involved in redox clearance, like peroxiredoxines, carboxyestherases, lipid droplet formation proteins, and the laminin and catepsin family; or metabolic signaling molecules, like carboxylesterase 1C, 14-3-3 protein, and dipeptidyl peptidase 3.

**Figure 6:**
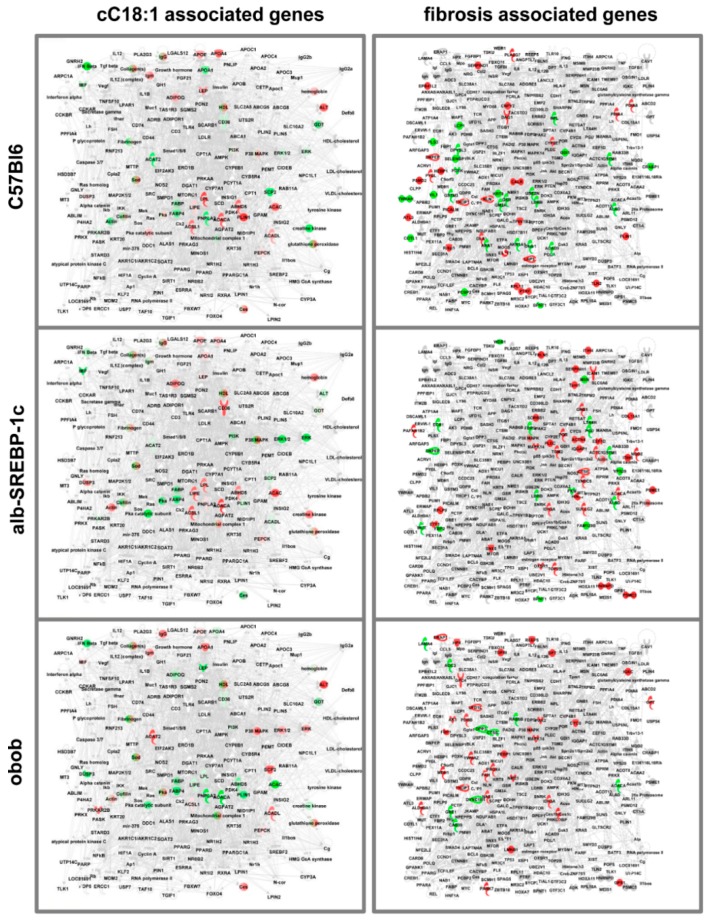
Differential abundance of FA metabolism and steatosis-related proteins in adipokines correlated to the “liver-to-adipose-tissue DNL” ratio. Virtual pathways were generated from proteins related to the keywords to cC18:1 or fibrosis. The proteins that correlated with the “liver-to-adipose-tissue DNL” ratio were used for IPA^®^ core analyses to extend the information to upstream regulating proteins (Supplementary Materials Table S6). Coloring represents the different abundances in the comparisons of C57Bl6 vs. alb-SREBP-1c, C57Bl6 vs. obob, and alb-SREBP-1c vs. obob. (Red: more abundant; green: less abundant; grey: not regulated in the specific comparison).

For the functional annotation, the proteins that correlated with the DNL ratio were used for IPA^®^ core analyses to extend the information to upstream regulating proteins. As our analyses revealed a marked difference in the abundance of proteins known to be regulated by or related to fatty acid metabolism or fibrosis (Figure 6), we generated virtual pathways for all proteins related to these keywords in order to visualize the IPA^®^ analyses. Based on these pathways, lean and healthy obese mice, due to increased hepatic DNL, showed similar patterns, in contrast to the “sick” obese obob mice (Figure 6; differential adipokines and upstream regulator molecules that causes different patterns were summarized in Table S6A,B).

## 3. Discussion

In the present study, we provide evidence that (i) the “liver-to-adipose-tissue DNL” ratio shows genotype-specific differences; (ii) this DNL-ratio can be monitored in the serum lipid pattern; and (iii) the pattern of the secreted proteins of adipocyte cells are different, indicated as a shift from secreted proteins, mainly involving tissue remodeling in lean and “healthy” obese mice, to metabolic active adipokines and fibrosis in morbid obese mice, corresponding to the health status of the adipose tissue.

To determine the systemic interaction of liver and adipose tissue in obesity, we choose a mouse model with obesity, according to hyperphagia and leptin deficiency (obob). Obob mice are an accepted morbid obesity model with a fatty liver, which develops cardiovascular complications and increased oxidative stress, including increased macrophage infiltration in adipose tissue and an inflammatory marker concertation [18,19,20]. On the other hand, we used a transgenic animal with a liver-specific overexpression of human SREBP-1c, which has previously been shown to induce obesity under isocaloric conditions due to increased hepatic DNL [7,8]. In these mice, a fatty liver, as an initial pathophysiological burden, is captured by the massively increasing visceral fat mass. The development of massive obesity in these mice is only accompanied by hepatic insulin resistance (IR), but without signs of inflammation in serum or adipose tissue. Therefore, alb-SREBP-1c mice resemble a “healthy” obesity phenotype, compared to obob mice. In regard to insulin secretion, alb-SREBP-1c mice showed a compensatory beta cells effect, whereas the beta cells failed to offset IR in obob mice. Nevertheless, IR poses a risk, as it increases lipolysis in adipose tissue, resulting in a release of fatty acids to serum, which finally elevates the triglyceride content in the liver [21].

The models showed a characteristic profile, with a decreased saturated C16:0 and C18:0 in the obese model, accompanied by increased levels of desaturated FA in serum. The main sources for the composition of the serum lipid profiles are nutrition, tissue-specific DNL, and the mobilization of FA by lipolysis from adipose tissue [22]. However, the tissue-specific FA patterns we determined in the liver and adipose tissue did not completely account for the different serum lipid patterns. Overall, adipose tissue lipolysis does not seem to be essential for explaining the differences seen in the serum FFA composition. In contrast, alterations observed in the hepatic FA, especially for cC16:1 and cC18:1, were also seen in serum lipids, except in the EFA cC18:2. There is controversy regarding the role of serum cC16:1 in health, as it increases insulin sensitivity in healthy subjects [23,24] but has adverse effects in obesity [25]. On the other hand, insulin resistance or the progression of NAFLD to non-alcoholic liver steatosis is accompanied by an increase of lipids, including cC16:1 and cC18:1 [22]. In NAFLD, cC16:1 and its elongation product, cC18:1, were increased due to the SREBP-1c-regulated increased Δ9 stearoyl-CoA desaturase 1 (SCD-1) activity [22], as seen in the alb-SREBP-1c model. Hepatic DNL was higher in alb-SREBP-1c mice, which is consistent with our previous observation [8]. Especially, the essential cC16:1 is, in general, very present in adipose tissue, making it a direct product and marker for adipose tissue DNL, and its presence in the serum lipid pool favors a role in signaling [23,26,27]. Thus, the data derived support the hypothesis that DNL in adipose or liver tissue might have further physiological functions beyond simple nutrient conversion.

Adipose tissue secretome has been thoroughly studied in metabolic disturbances and has been accepted as a model for various metabolic alterations [10,11,12,17]. As the FA composition of adipocytes was not grossly altered, thus excluding a predominant lipokine, adipocyte-secreted adipokines might act as a signaling moiety to adjust tissue-specific DNL rates. Overall, analyses of the adipokinome indicated a close relation between the adipokinomes of C57Bl6 and those of alb-SREBP-1c mice. This might indicate the healthy status of the adipose tissue in alb-SREBP-1c mice, in contrast to obob mice.

In proteins with a secretion motive, both obesity models differed in regard to proteins associated with metabolic processes. There was an accumulation of proteins found to be related to or involved in fibrosis-associated processes in obob mice, compared to the controls. This is consistent with the excess accumulation of adipose tissue extra cellular matrix (ECM) components and IR in obesity [28], and to a recent observation of the ECM organization and assembly markers that were increased in obese humans with a high serum FFA mobilization from adipose tissue [29].

Proteins without a secretion signal are probably related to the endomembrane system for vesicular secretion processes [30]. In this context, we have recently shown that adipose tissue-secreted exosomes are enriched in relevant metabolic proteins, without signal peptides [31]. Here, both obesity models differed in proteins related to, e.g., proteosomal degradation processes. In obesity, a proteasome dysfunction further aggravates the cell toxic effects of increased oxidative stress or unfolded protein responses to ER stress in adipocytes. Proteasome function has also been found to maintain insulin sensitivity in adipocytes [32]. In the context of adipose tissue, this might indicate that the fibrosis of the adipose organ is a marker for functionality defects, which is in line with previous observations [10,11,12].

The most striking difference within the obese mouse models was still that the “healthy” obese alb-SREBP-1c mice showed the largest difference in the “liver-to-adipose-tissue DNL” ratio, and the serum cC16:1 and cC18:1 correlated to adipose tissue DNL solely in the alb-SREBP-1c mice. The correlative adipokines indicated a specific and gradual difference for the obesity models, but the patterns were not conclusive of a certain pathway. Nevertheless, a differential accumulation of cC18:1- and fibrosis-associated proteins in the adipokinome can be determined.

The interaction of adipose tissue and hepatic DNL is in a tight balance in lean healthy conditions, but runs out of control in obesity, IR, or NAFLD [33,34,35]. In obesity, adipose DNL is reduced [36] and can be restored by caloric restriction [37]. Increased adipocyte DNL seems to be beneficial in regard to IR or glucose homeostasis, independent of obesity in humans [4,13,38]. This idea identified adipocyte-derived lipokines, cC16:1 and cC18:1, as essential systemic mediators that interfere with adipose tissue physiological functionality, with hepatic lipid metabolism and DNL [38,39]. In conclusion, whole body energy homeostasis is mainly dependent on hepatic and adipose tissue communication. The concept of communication by -kines identified cC16:1 and cC18:1 as adipocyte-derived DNL products, as mediators for the adipocyte DNL status to the liver [10,11,12,27,38].

Our study indicated that the obesity models differ in circulating cC16:1, compared to lean controls, and further by cC18:1, which might act as marker. As the concentration of the lipokine cC18:1 also differs in the serum in the obese models, our observations point to a central role of adipokines as indicators that differentiate healthy and metabolically diseased models. It is noteworthy that a recent study of a mouse model showed that oleate (cC18:1) specifically featured a nuclear accumulation of the master-regulator SREBP-1c of the DNL in hepatocytes, suggesting cC18:1 as central in SREBP-1c-mediated signaling and therefore in DNL [40]. This further supports the hypothesis that an increase in hepatic DNL is essential for maintaining adipose tissue health, and adipokine secretion is part of the systemic regulation.

The composition of the serum lipids reflects the “liver-to-adipose-tissue DNL” ratio. Interestingly, adipokine patterns that correlate with the “liver-to-adipose-tissue DNL” ratio seem to be rather phenotype-specific, as only a marginal overlap can be observed in the models. However, in both obese models, but not in the controls, mitogen-activated protein kinase 14 or isocitrate dehydrogenase show a comparable correlation, whereas acetyl-CoA carboxylase 1, catalyzing the rate-limiting step in the lipid synthesis of malonyl-CoA synthesis from acetyl-CoA, is inversely correlated in healthy and sick obesity. On the other hand, proteins involved in free radical scavenging, like glutathione S-transferase Mu 2, were equally correlated in the healthy obese and the control animals. This, and observation that peroxisomal ECH accounts for alb-SREBP-1c, further supports our previous finding, i.e., that peroxisomal function might play a role in preventing increased hepatic lipid accumulation in metabolic syndrome or diabetes [17,41].

Our data further support the idea that the adipose tissue returns information back to the liver also regarding its status of plasticity and metabolic capacity. Thus, depending on the degree of the “liver-to-adipose-tissue DNL” ratio, adipokinomes show fibrotic pathways as a marker for the beginning of a loss of adipose tissue health.

Conclusion: our analyses features the concept of impaired adipose tissue functionality in obesity. We provide evidence that the “liver-to-adipose-tissue DNL” ratio is a marker for the shift from healthy to diseased adipose tissue. Adipose tissue “health” can be maintained in obesity as long as the hepatic DNL can be increased according to the metabolic requirements. This can be monitored by serum fatty acid cC16:1 and especially cC18:1, as biomarkers, and is accompanied by altered adipose tissue secreted proteins, as the adipokinome of “healthy” or “sick fat” differs in regard to cC16:1 and cC18:1 and fibrosis-dependent proteins.

## 4. Materials and Methods

### 4.1. Animals

C57Bl6 (C57Bl6), B6.Cg-Lep^ob^ (obob), and B6-TgN(alb-HA-SREBP-1cNT) (alb-SREBP-1c) [7] mice were bred and maintained under standard conditions (12h light/dark cycle; 22 ± 1 °C, 50% ± 5% humidity). At 6 weeks of age, male littermates of each genotype were kept under standardized conditions, with free access to water and regular laboratory chow (13.7 mJ/kg: 53% carbohydrate, 36% protein, 11% fat (Ssniff, Soest, Germany)). At the age of 18 weeks, the mice were fasted for 6 h and sacrificed by CO_2_ asphyxiation (7:00 am). Blood samples were collected by a left ventricular puncture, and organ samples, i.e., the liver and visceral adipose tissue, were removed. The Animal Care Committee of the University Duesseldorf approved the animal care and procedure employed (Approval#84-02.04.2015.A424; 2 April 2015).

### 4.2. Animal Characterization

Phenotypical characterization; serum diagnostics of clinical measures; as well as the surrogate parameters of insulin resistance, lipid profiling in serum, and liver and adipose tissue by gas chromatography were performed, as previously described [7,42]. Serum-free fatty acids (FFA), hepatic as well as adipose cell total fatty acids (TFA) content, and the specific fractional composition of FAs were determined by gas chromatography. FA data of adipocytes were further used to calculate the Δ5-desaturase index (cC18:2/cC20:4); Δ6-desaturase index (cC18:2/cC18:3; Δ9-desaturase index (cC16:1/C16:0 or cC18:1/C18:0); DNL index (C16:0/cC18:2); elongation index (C18:0/C16:0); as well as the sums of the total FA, non-saturated FA, monounsaturated FA, saturated FA, essential FA (cC18:2+cC18:3), and non-essential FA (C16:0+cC16:1+C18:0+cC18:1) [43]. The nomenclature of FA is given according to IUPAC. The liver-to-adipose-tissue DNL was calculated by the liver DNL/adipose tissue DNL.

### 4.3. Secretome Profiling by Liquid Chromatography (LC)-Electrospray Ionization (ESI)-MS/MS and Data Analyses

Mature adipocytes were isolated from minced biopsies by collagenase digestion. Adipocytes were cultured (2 days), washed extensively, and supplemented with an FCS-free culture medium to harvest and process the secretome, as previously described [17,44]. Data on all mouse models were acquired in parallel, as described in detail previously [17,45].

### 4.4. Data Annotation

The functional annotation and prediction of secretory proteins was performed with SignalP 4.1 [46], (http://www.cbs.dtu.dk/services/SignalP/), SecretomeP 2.0. [47], (http://www.cbs.dtu.dk/services/SecretomeP/). We identified 922 individual unique proteins in the secretomes of the mouse models investigated (Supplementary Materials Table S1). Of all proteins, 543 (59%) are characterized as classically or non-classically secreted proteins (SP+/SP−). The remaining 379 identified proteins do not contain classical secretion signals.

### 4.5. Statistical Analysis

Clinical values are presented as the mean ± SD. Statistical analysis was performed with Student’s *t*-test or one-way ANOVA, with a Sidak post hoc test, calculated with Prism 7.04 (GraphPad Software Inc., San Diego, CA, USA), as indicated. Secretome data were further analyzed with the Metabolist 3.0 package [48] or SPSS (IBM Ver. 22). Pearson correlation coefficients, with a two-sided *p*-value, were determined in SPSS (IBM Ver. 22).

### 4.6. Web-Based Functional Annotation

For the functional annotation, web-based tools from public database sources were used: https://www.ncbi.nlm.nih.gov/, http://www.informatics.jax.org/mgihome/, http://bioinfo.vanderbilt.edu/webgestalt/ [49], https://toppcluster.cchmc.org/ [50], David Bioinformatics Resources 6.8 (https://david.ncifcrf.gov/) [51], and IPA^®^ (Ingenuity^TM^, Qiagen, Hilden, Germany). The fold change of different adipokinome patterns was analyzed, and the *t*-test-derived *p*-values of the comparisons C57Bl6 vs. alb-SREBP-1c, C57Bl6 vs. obob, and alb-SREBP-1c vs. obob were entered for the IPA^®^ analyses. Furthermore, a spearman coefficient and two-sided *p*-value were used. Data were used for core analyses and comparison analyses. The pathways were generated from respective networks, as suggested by IPA^®^. For expression analyses of different protein sets, an expression fold change (1.5×) and expression differences (*p*-value <0.05) were analyzed, following the core analysis modules. Differentially abundant proteins (1.5× fold difference, *p*-value < 0.05) (one–way ANOVA, posthoc, Welch test) were analyzed separately for C57 vs. obob, C57 vs. alb-SREBP-1c, and alb-SREBP-1c vs. obob mice.

## Figures and Tables

**Table 1:** Metabolic characterization of the lean mice (C57Bl6), transgenic mice (alb-SREBP-1c) with “healthy” adipose tissue, and of the “sick” adipose tissue obob mice used in the study.

Parameter	C57Bl6	alb-SREBP-1c	obob
**body weight [g]**	28.62 ± 2.54	35.23 ± 3.44 **	56.82 ± 6.76 **
**liver weight [g]**	1.56 ± 0.20	2.10 ± 0.30 **	3.72 ± 0.80 **
**fat mass [g]**	0.40 ± 0.13	1.79 ± 0.57 **	5.31 ± 0.87 **
**blood glucose [mg/dL]**	148.4 ± 15.24	184.41 ± 10.01 **	769.20 ± 142.31 **
**cholesterol [mg/dL]**	92.67 ± 12.98	111.18 ± 22.01	140.13 ± 33.70 **
**triglycerides [mg/L]**	123.60 ± 16.07	244.00 ± 52.86 **	403.60 ± 54.47 **
**ALT [U/L]**	48.75 ± 22.15	78.45 ± 13.19 **	181.87 ± 46.14 **
**AST [U/L]**	102.67 ± 24.19	156.36 ± 35.77 **	274.80 ± 102.36 **
**GLDH [U/L]**	13.46 ± 6.08	29.36 ± 13.68 **	139.75 ± 66.59 **
**insulin [ng/mL]**	1.06 ± 0.24	3.90 ± 1.39 **	16.51 ± 3.05 **
**leptin [ng/mL]**	0.90 ± 0.60	12.08 ± 3.48 **	n.d.
**HOMA-IR**	0.38 ± 0.0.08	2.12 ± 0.36 **	34.43 ± 3.05 **
**HOMA-β%**	96.26 ± 35.21	324.19 ± 175.06 **	180.71 ± 94.68 **
**food uptake/bodyweight (kJ/g)**	10.33 ± 2.95	11.84 ± 2.61	16.06 ± 1.79 **
**weight gain/food uptake (mg/kJ)**	1.55 ± 0.19	2.48 ± 0.25 **	3.14 ± 0.45 **

Data are expressed as mean ± SD (*n* = 8 of each phenotype). * *p* < 0.05, ** *p* < 0.01 ***, and *p* < 0.001, by Student’s *t*-test in comparison to controls.

## Supplementary Materials

Supplementary Materials can be found at https://www.mdpi.com/1422-0067/20/10/2559/s1.

## Abbreviations

ALT	alanine transaminase
AST	aspartate transaminase
BG	blood glucose
BG	blood glucose
DNL	de novo lipogenesis
ECM	extra cellular matrix
EFA	essential FA
ER	endoplasmatic reticulum
FA	fatty acids
FCS	fetal calf serum
FFA	free fatty acids
GLDH	glutamate dehydrogenase
GO	gene ontology
HOMA-%β	Homeostatic model assessment of β -cell function (%)
HOMA-IR	Homeostatic model assessment of insulin resistance
IR	insulin resistance
MS	mass spectrometry
MUFA	monounsaturated fatty acids
NAFLD	nonalcoholic fatty liver disease
NEFA	non-saturated FA
PCA	principal component analysis
PLS-DA	partial least square discriminant analysis
PUFA	poly unsaturated fatty acids
SCD1	Δ9 stearoyl-CoA desaturase 1
SFA	saturated fatty acids
SREBP	sterol regulatory element-binding protein
T2D	type-2 diabetes mellitus
TFA	total fatty acids
TG	triglycerides
UFA	unsaturated fatty acids
WAT	white adipose tissue
