# Supplementary material for: Adipokinome Signatures in Obese Mouse Models Reflect Adipose Tissue Health and Are Associated with Serum Lipid Composition
Source: Int J Mol Sci. 2019 May 24;20(10):2559. doi: 10.3390/ijms20102559 (PMC6567124; doi:10.3390/ijms20102559)
Supplement: Supplementary file 1 [file ijms-20-02559-s001.zip › IJMS Table S4.docx]

**Supplement Table 4: Enrichment analyses of adipokines**

**A: Annotation of SP+/SP- proteins**

The results for the enriched GO category and corresponding GO ID are listed. C: the number of reference genes in the category; O: the number of genes in the gene set and also in the category; E: the expected number in the category ; R: ratio of enrichment; rawP: p value from hypergeometric test; adjP: p value adjusted by the multiple test adjustment. Analyses were performed with http://bioinfo.vanderbilt.edu/webgestalt. Leading genes are highlighted (bold).

| **Cell modification**  **Leading genes C57Bl6 alb-SREBP-1c** | **Database biological process/ molecular function name** | **stat** | **metabolism**  **Leading genes C57Bl6 alb-SREBP-1c** | **Database biological process/ molecular function name** | **stat** |
| --- | --- | --- | --- | --- | --- |
| Ezr; Lyz2; Cfl1; Ctsd; Parva; Ak3; Vat1; Lama5; Hnrnpab; **Calr**; Il6; Ddx39b; Ap1m1; Hspg2; Actc1; **Vim**; Scp2; Arpc4; Dstn; Lpl; Tppp3; Anxa2; Arpc3; Dpt; Acadl; Phgdh; Fbln5; Erap1; Col4a2; **Atp5b**; Dld; Msn | cellular component organization or biogenesis; IDGO0071840 | C=3588; O=27; E=9.50; R=2.84; rawP=7.58e-08; adjP=4.67e-05 | Cfl1; Ctsd; Pcbp2; Ak3; **Fth1**; Lama5; Cxcl1; **Calr**; Sdpr; Ddx39b; Ykt6; Hspg2; Scp2; **Vim**; Msn; Dld; Arpc4; Dstn; Anxa2; Bcam; Arpc3; Mgll; Acadl; Ivd; Snx2; Sardh; Ezr; Rab11b; Ptrf; Pcbp1; Parva; Suox; Erap1; Vat1; Hnrnpab; Il6; Mpp1; Prelp; Lbp; **Atp5b**; Actc1; Lpl; Tppp3; Uqcrfs1; Prkcdbp; Mmp3; Rpl12; Arhgap1; Cp; Phgdh; Fbln5; Col4a2; Ap1m1; Lyz2; Col6a5; Dpt; Cox6b1 | binding; IDGO0005488 | C=10781; O=51; E=28.76; R=1.77; rawP=7.96e-10; adjP=8.36e-08 |
|  | cellular component organization; IDGO0016043 | C=3466; O=26; E=9.18; R=2.83; rawP=1.66e-07; adjP=5.11e-05 |  | cellular process; IDGO0009987 | C=12976; O=51; E=34.36; R=1.48; rawP=1.71e-06; adjP=0.0002 |
|  | cellular component organization at cellular level; IDGO0071842 | C=2756; O=22; E=7.30; R=3.01; rawP=8.54e-07; adjP=0.0002 |  | coenzyme binding; IDGO0050662 | C=180; O=7; E=0.48; R=14.58; rawP=5.00e-07; adjP=1.75e-05 |
|  | cellular component organization or biogenesis at cellular level; IDGO0071841 | C=2866; O=22; E=7.59; R=2.90; rawP=1.67e-06; adjP=0.0002 |  | cofactor binding; IDGO0048037 | C=254; O=7; E=0.68; R=10.33; rawP=4.95e-06; adjP=8.66e-05 |
|  | actin cytoskeleton organization; IDGO0030036 | C=370; O=8; E=0.98; R=8.17; rawP=5.64e-06; adjP=0.0006 |  | ferroxidase activity; IDGO0004322 | C=4; O=2; E=0.01; R=187.42; rawP=4.18e-05; adjP=0.0004 |
|  | actin filament organization; IDGO0007015 | C=194; O=6; E=0.51; R=11.68; rawP=1.23e-05; adjP=0.0009 |  | flavin adenine dinucleotide binding; IDGO0050660 | C=68; O=4; E=0.18; R=22.05; rawP=3.27e-05; adjP=0.0004 |
|  | anatomical structure morphogenesis; IDGO0009653 | C=1821; O=16; E=4.82; R=3.32; rawP=1.34e-05; adjP=0.0009 |  | oxidoreductase activity; IDGO0016491 | C=737; O=11; E=1.97; R=5.59; rawP=3.36e-06; adjP=8.25e-05 |
|  | actin filament-based process; IDGO0030029 | C=413; O=8; E=1.09; R=7.32; rawP=1.25e-05; adjP=0.0009 |  | oxidoreductase activity, oxidizing metal ions, oxygen as acceptor; IDGO0016724 | C=4; O=2; E=0.01; R=187.42; rawP=4.18e-05; adjP=0.0004 |
|  | cytoskeleton organization; IDGO0007010 | C=716; O=10; E=1.90; R=5.27; rawP=1.65e-05; adjP=0.0010 |  | protein binding; IDGO0005515 | C=6181; O=37; E=16.49; R=2.24; rawP=2.23e-08; adjP=1.17e-06 |
|  | cytoskeletal protein binding; IDGO0008092 | C=603; O=10; E=1.61; R=6.22; rawP=3.93e-06; adjP=8.25e-05 |  |  |  |
|  | actin binding; IDGO0003779 | C=322; O=7; E=0.86; R=8.15; rawP=2.30e-05; adjP=0.0003 |  |  |  |
| **Cell modification Leading genes obob alb-SREBP-1c Gene Symbol** | **Database biological process/ molecular function name** | **stat** | **metabolism**  **Leading genes obob alb-SREBP-1c** | **Database biological process/ molecular function name** | **stat** |
|  |  |  | Acadvl; Ldha; Gsto1; Acaa1a; **P4hb**; Crat; Acaa2; Gpx1; Adh1; Aldh1a1; Dld; Cryl1; Got2; Aldh6a1; Fh1; Dlst; Ogdh; **Adipoq**; **Ldhb**; Mgll; Acly; Apoa4; Ahcy; Acadl; Thnsl2; Dcn; Gstm2; Sardh; Atic; Sod1; Acox1; Pdhb; Cd36; Acsl1; Pdha1; Hadh; Gsr; Ephx2; Gstt2; Cs; Lpl; Acadm; Bgn; Gstz1; Gstm1; Decr1; Hibadh; Hsd17b4; Phgdh; Acss2; Lep; Pgcp; Ttr; Car2; **Cyb5**r3; Rab11b; Tubb2a; Actc1; Car5b; Ak3; Txn1; Sord; H6pd; Hprt; Mccc2; Gyk; Pdxk; **Gc**; **Gda**; Fbp1; **Atp6v1b2**; Gpd1; Il6; **Atp5b**; Cbr1; Tpm1; Rbp4; **Fth1**; Acad9; Fuca1; Gstm7; Serpinh1; Art3; Acy3; Prcp; Pla2g7; Hpx; Gyg; Ces1d; Psap; Mmp2; Cox6b1; Cpe; Ctsa; Ctsl; Pecam1; Mmp3; Grpel1; Ctsz; Serpina3c; Aoc3; Fbln5; Ecm1; Ace; Lgmn; Lum; Col4a2; Inmt; Pnpla2; Ptx3; Ephx1; Msra; Psmb6; Capn1; Glrx3; Fbp2; Lcn2; Qsox1; **Serpina1**e; Col3a1; Col1a1; Clu; Aldh7a1; Ces1c; Ddx39b; Pccb; Prdx3; Trf; C4b; C2; Acot9; Dcps; Kng1; Lyz2; Efemp1; Pzp; **Iah1**; Lbp; Plg; Cfd; **Alad**; Eef1d; Pfn1; Psmb4; Ctsd; Rarres2; Ppic; Tgm2; Bpnt1; **Serpina1**d; Blvra; Ube2k; Csf1; Pcca; Tnc; Timp1; Amy1; Blmh; Cbr3; Hmox1; Gm2a; **Serpina1**b; Cyp2e1; Psmb8; Hfe; Eml2; Lactb2; 4931406C07Rik; Tst; Ear1; Hrsp12; Acyp2; **Alb**; Tgfbi; Lama4; Coro1b; Cdh16; Gpc4; Cdh13; Coro1a; Col18a1; Lgals3bp; Anxa7; Tppp3; **Ogn**; Lama5; Cav2; Arpc3; **Capzb**; Prrc1; Ezr; Ccl9; **Lcp1**; Ap2b1; Lamb2; Col1a2; Retn; Crabp1; Rpl12; Kctd12; **Gsn**; Rab6b; Cotl1; Snx2; Sec23a; Col4a1; Fstl1; Selenbp1; Ccdc80; Prelp | oxoacid metabolic process; IDGO0043436 | C=717; O=52; E=6.94; R=7.49; rawP=9.23e-31; adjP=1.44e-27 |
|  |  |  |  | organic acid metabolic process; IDGO0006082 | C=731; O=52; E=7.07; R=7.35; rawP=2.36e-30; adjP=1.44e-27 |
|  |  |  |  | small molecule metabolic process; IDGO0044281 | C=1821; O=77; E=17.62; R=4.37; rawP=2.68e-30; adjP=1.44e-27 |
|  |  |  |  | carboxylic acid metabolic process; IDGO0019752 | C=675; O=50; E=6.53; R=7.65; rawP=5.63e-30; adjP=2.26e-27 |
|  |  |  |  | metabolic process; IDGO0008152 | C=8894; O=165; E=86.08; R=1.92; rawP=1.39e-28; adjP=4.47e-26 |
|  |  |  |  | oxidation-reduction process; IDGO0055114 | C=919; O=55; E=8.89; R=6.18; rawP=2.29e-28; adjP=6.14e-26 |
|  |  |  |  | catabolic process; IDGO0009056 | C=1645; O=71; E=15.92; R=4.46; rawP=3.13e-28; adjP=7.19e-26 |
|  |  |  |  | single-organism metabolic process; IDGO0044710 | C=8262; O=153; E=79.96; R=1.91; rawP=1.57e-24; adjP=3.16e-22 |
|  |  |  |  | organic substance catabolic process; IDGO1901575 | C=1515; O=62; E=14.66; R=4.23; rawP=3.64e-23; adjP=6.51e-21 |
|  |  |  |  | cellular catabolic process; IDGO0044248 | C=1393; O=59; E=13.48; R=4.38; rawP=1.00e-22; adjP=1.61e-20 |
|  |  |  |  | catalytic activity; IDGO0003824 | C=5105; O=131; E=49.78; R=2.63; rawP=7.15e-33; adjP=1.89e-30 |
|  |  |  |  | oxidoreductase activity; IDGO0016491 | C=737; O=45; E=7.19; R=6.26; rawP=2.50e-23; adjP=3.30e-21 |
|  |  |  |  | cofactor binding; IDGO0048037 | C=254; O=29; E=2.48; R=11.71; rawP=1.23e-22; adjP=1.08e-20 |
|  |  |  |  | binding; IDGO0005488 | C=10781; O=172; E=105.13; R=1.64; rawP=1.18e-21; adjP=7.79e-20 |
|  |  |  |  | coenzyme binding; IDGO0050662 | C=180; O=23; E=1.76; R=13.10; rawP=2.91e-19; adjP=1.54e-17 |
|  |  |  |  | protein binding; IDGO0005515 | C=6181; O=119; E=60.27; R=1.97; rawP=2.20e-17; adjP=9.68e-16 |
|  |  |  |  | identical protein binding; IDGO0042802 | C=864; O=40; E=8.43; R=4.75; rawP=1.57e-16; adjP=5.92e-15 |
|  |  |  |  | NAD binding; IDGO0051287 | C=50; O=12; E=0.49; R=24.61; rawP=4.74e-14; adjP=1.56e-12 |
|  |  |  |  | hydrolase activity; IDGO0016787 | C=2189; O=55; E=21.35; R=2.58; rawP=2.92e-11; adjP=8.57e-10 |
|  |  |  |  | protein homodimerization activity; IDGO0042803 | C=581; O=26; E=5.67; R=4.59; rawP=1.07e-10; adjP=2.82e-09 |
| **Leading genes C57Bl6**  **obob Gene Symbol** | **Database biological process/ molecular function name** | **stat** | **metabolism**  **Leading genes C57Bl6**  **obob** | **Database biological process/ molecular function name** | **stat** |
| Lama4; Tgfbi; Car2; Cdh16; Serpinh1; Ap1m1; Cdh13; Hspg2; Scp2; Cryl1; Got2; Bcam; Ndufv2; Coro1a; Col18a1; Lgals3bp; Pla2g7; Hpx; Cxcl3; Thnsl2; Gyg; Sardh; Pcbp1; Col6a5; Ces1d; Ppp2r4; Psap; Anxa7; Aldh1a7; Mmp2; Ephx2; Gstt2; Ctsl; Kpna3; Mmp3; Gstm1; **Ogn**; Aoc3; Fbln5; Pcbp2; Ace; Gsto1; Ak3; Txn1; Lama5; Lum; Sord; Col4a2; H6pd; Ppib; Hprt; Ptx3; Arpc4; Dlst; Cav2; Arpc3; Gyk; Ahcy; Ephx1; **Capzb**; **Alb**; Fbp1; Capn1; Cd36; Gpd1; Vat1; **Lcp1**; Acsl1; Gsr; Nid2; Qsox1; Cbr1; Lcn2; Col1a2; Lamb2; Fam82a2; Retn; Bgn; Col3a1; Decr1; Ldhc; Ube2n; Kctd12; Col1a1; Clu; Rbp4; Pgcp; Cfl1; Cct8; Ttr; Cxcl1; Ak2; Ykt6; **Adipoq**; Tst; **Gsn**; Nrp1; Acot9; Rragc; **Ldhb**; Apoa4; Rab6b; Acadl; Dcps; Gstm2; Snx2; Kng1; Lyz2; Fbln1; Sod1; Efemp1; Ptrf; **Actg1**; Isyna1; Tubb2a; Mpp1; Tagln; Cs; Serpinf1; Cfd; Sec23a; Uqcrfs1; Nid1; **Alad**; Cst3; Hsp90b1; Dpt; Hibadh; Pfn1; Eif3k; Twf1; Acadvl; Ldha; Apoh; Ppic; Tgm2; Bpnt1; Erp29; Sdpr; Acaa2; Aldh1a1; Adh1; **Vim**; Msn; Col4a1; Blvra; Ube2k; Napa; Anxa2; Csf1; Prdx5; **Selenbp1**; Coro1c; Pdxk; Dcn; Timp1; **Gda**; Atic; Parva; Acox1; Ccdc80; **Atp6v1b2**; Il6; Col6a1; Pdia4; Ipo5; Cbr3; Hmox1; Tpm1; **Serpina1**b; Gm2a; Cyp2e1; Ltbp4; Echs1; Arhgap1; Psmb8 | cellular process; IDGO0009987 | C=12976; O=176; E=122.62; R=1.44; rawP=5.70e-16; adjP=1.22e-13 | Acadvl; Ldha; Gsto1; Txn1; Acad9; Sord; H6pd; Acaa2; Aldh1a1; Adh1; Cryl1; Blvra; Dlst; **Cyb5**r3; Ndufv2; **Adipoq**; Prdx5; Pla2g7; **Ldhb**; Apoa4; Acadl; Gyg; Sardh; Sod1; Acox1; Suox; Cd36; Gpd1; Vat1; Aldh1a7; Gsr; Pdia4; Cbr1; Qsox1; Cbr3; Cs; Hmox1; Uqcrfs1; Cyp2e1; Echs1; Decr1; Ldhc; Cp; Aoc3; Hibadh; Aldh7a1; Ak3; Ttr; Car2; Ak2; Hprt; Scp2; Got2; Gyk; Rragc; Ahcy; Pdxk; Thnsl2; Dcn; **Gc**; **Gda**; Gstm2; Atic; Fbp1; **Atp6v1b2**; Ppp2r4; Isyna1; Il6; Tubb2a; Acsl1; Ephx2; Gstt2; Tpm1; Bgn; Arhgap1; Gstm1; Pgcp; Rbp4; Fuca1; Gstm7; Serpinh1; Hpx; Pcbp1; Ces1d; Psap; Mmp2; Ctsl; Mmp3; Fbln5; Ctsc; Pcbp2; Ace; Cstb; Lum; Col4a2; Inmt; Ppib; Ptx3; Ephx1; Capn1; Fbp2; Lcn2; **Serpina1**e; Col3a1; Ube2n; Col1a1; Clu; Cfl1; Cct8; Ces1c; Ykt6; C4b; C2; Nrp1; Acot9; Dcps; Kng1; Lyz2; Fbln1; Efemp1; Ptrf; Erap1; Pzp; **Iah1**; Cfd; **Alad**; Cst3; Hsp90b1; Eif3k; Pfn1; Twf1; Apoh; Ppic; Tgm2; Sdpr; Bpnt1; **Serpina1**d; Ube2k; Csf1; Anxa2; Timp1; Amy1; Blmh; **Serpina1**b; Gm2a; Ltbp4; Psmb8; Hfe; Cxcl1; Cdh13; Coro1a; **Gsn**; Cxcl3; Anxa7; Mpp1; Col4a1; Ccl9; Col6a1; Lamb2; Col1a2; Retn; Lama4; Tgfbi; Cdh16; Eml2; Gpc4; Hspg2; Bcam; Col18a1; Lgals3bp; S100a11; Kpna3; **Ogn**; Lama5; Arpc4; Cav2; Arpc3; **Capzb**; **Alb**; **Lcp1**; Nid2; Anxa4; Kctd12; Des; Tst; Rab6b; Ear1; Snx2; **Actg1**; Sec23a; Nid1; Prkcdbp; Vim; Msn; Fstl1; Napa; **Selenbp1**; Coro1c; Parva; Ccdc80; Ipo5; Hrsp12; Acyp2 | small molecule metabolic process; IDGO0044281 | C=1821; O=63; E=17.21; R=3.66; rawP=2.32e-20; adjP=1.73e-17 |
|  |  |  |  | oxidation-reduction process; IDGO0055114 | C=919; O=46; E=8.68; R=5.30; rawP=6.61e-21; adjP=9.88e-18 |
|  |  |  |  | metabolic process; IDGO0008152 | C=8894; O=148; E=84.05; R=1.76; rawP=1.58e-19; adjP=7.87e-17 |
|  |  |  |  | response to chemical stimulus; IDGO0042221 | C=2020; O=64; E=19.09; R=3.35; rawP=9.63e-19; adjP=3.60e-16 |
|  |  |  |  | catabolic process; IDGO0009056 | C=1645; O=57; E=15.55; R=3.67; rawP=2.40e-18; adjP=7.17e-16 |
|  |  |  |  | single-organism metabolic process; IDGO0044710 | C=8262; O=136; E=78.08; R=1.74; rawP=2.51e-16; adjP=6.25e-14 |
|  |  |  |  | oxoacid metabolic process; IDGO0043436 | C=717; O=35; E=6.78; R=5.17; rawP=1.22e-15; adjP=2.28e-13 |
|  |  |  |  | organic acid metabolic process; IDGO0006082 | C=731; O=35; E=6.91; R=5.07; rawP=2.19e-15; adjP=3.64e-13 |
|  |  |  |  | carboxylic acid metabolic process; IDGO0019752 | C=675; O=33; E=6.38; R=5.17; rawP=8.41e-15; adjP=1.26e-12 |
|  |  |  |  | binding; IDGO0005488 | C=10781; O=176; E=101.66; R=1.73; rawP=6.39e-28; adjP=1.71e-25 |
|  |  |  |  | protein binding; IDGO0005515 | C=6181; O=129; E=58.28; R=2.21; rawP=6.70e-25; adjP=8.94e-23 |
|  |  |  |  | catalytic activity; IDGO0003824 | C=5105; O=107; E=48.14; R=2.22; rawP=3.93e-19; adjP=3.50e-17 |
|  |  |  |  | cofactor binding; IDGO0048037 | C=254; O=25; E=2.40; R=10.44; rawP=2.03e-18; adjP=1.36e-16 |
|  |  |  |  | oxidoreductase activity; IDGO0016491 | C=737; O=37; E=6.95; R=5.32; rawP=6.20e-17; adjP=3.31e-15 |
|  |  |  |  | coenzyme binding; IDGO0050662 | C=180; O=20; E=1.70; R=11.78; rawP=5.67e-16; adjP=2.52e-14 |
|  |  |  |  | ion binding; IDGO0043167 | C=5208; O=91; E=49.11; R=1.85; rawP=9.54e-11; adjP=3.18e-09 |
|  |  |  |  | NAD binding; IDGO0051287 | C=50; O=9; E=0.47; R=19.09; rawP=8.86e-10; adjP=2.63e-08 |
|  |  |  |  | identical protein binding; IDGO0042802 | C=864; O=38; E=8.15; R=4.66; rawP=1.66e-15; adjP=6.33e-14 |
|  |  |  |  | protein homodimerization activity; IDGO0042803 | C=581; O=24; E=5.48; R=4.38; rawP=1.46e-09; adjP=3.90e-08 |

____________________________________________________________________________________________________________________

**B: Annotation of NP-proteins**

| **Cell modification**  **Leading genes C57Bl6 alb-SREBP-1c** | **Database biological process/ molecular function name** | **stat** | **metabolism**  **Leading genes C57Bl6 alb-SREBP-1c** | **Database biological process/ molecular function name** | **stat** |
| --- | --- | --- | --- | --- | --- |
| Akap12, Ap2a2, Sorbs1, Ehd2, Ehd1, Mapre1, Eif5a, Nedd4, Glud1, Actr2, Picalm, Oxct1, Tln2, **Anxa3**, Sncg, Pdzk1, Actr3, Prkaca, Anxa1, Spr, Capza2, Fermt2, Arpc2, Aldob, Fhl1, Csrp1, Rdx, Nsfl1c, Hnrnpk, Psmd11, Abhd5, Naca, Pdcd6ip, Hnrnpd, Syncrip, **Ckb**, Eif2s3x, Eef1a1, Lipe, Eif2s1, Psme2, Psme1, Stard10, S100a10 | cellular localization, GO:0051641 | C=1600; O=19; E=3.29; R=5.78; rawP=8.93e-11; adjP=4.68e-08 | Ap2a2, Sorbs1, Capza2, Arpc2, **Ckb**, Mapre1, Pdcd6ip, Csrp1, Actr2, Glud1, Rdx, Picalm,  Hnrnpk, Psme1, Prkaca, Anxa1, Hnrnpd, Akap12, Abhd5, Ehd2, Naca, Ehd1, Eef1a1, Nedd4, Eif5a, Aldob, Fhl1, Oxct1, Tln2, Gstm5, Nsfl1c, Sncg, Lipe, Pdzk1, Actr3, Psme2, Syncrip, Eif2s3x, Eif2s1, Fermt2, Hspa12a, **Anxa3**, S100a10 | protein binding,  GO:0005515 | C=6181; O=36; E=12.79; R=2.81; rawP=9.86e-13; adjP=8.87e-11 |
|  | cellular component organization, GO:0016043 | C=3466; O=25; E=7.12; R=3.51; rawP=1.07e-09; adjP=2.80e-07 |  | binding, GO:0005488 | C=10781; O=43; E=22.31; R=1.93; rawP=2.02e-11; adjP=9.09e-10 |
|  | cellular component organization or biogenesis, GO:0071840 | C=3588; O=25; E=7.37; R=3.39; rawP=2.25e-09; adjP=3.93e-07 |  | phospholipase A2 inhibitor activity, GO:0019834 | C=3; O=2; E=0.01; R=322.09; rawP=1.26e-05; adjP=0.0003 |
|  | macromolecule localization, GO:0033036 | C=1599; O=17; E=3.28; R=5.18; rawP=6.71e-09; adjP=8.79e-07 |  | phospholipase inhibitor activity, GO:0004859 | C=7; O=2; E=0.01; R=138.04; rawP=8.74e-05; adjP=0.0009 |
|  | localization, GO:0051179 | C=3990; O=25; E=8.20; R=3.05; rawP=2.12e-08; adjP=2.22e-06 |  | lipase inhibitor activity, GO:0055102 | C=11; O=2; E=0.02; R=87.84; rawP=0.0002; adjP=0.0016 |
|  | protein localization, GO:0008104 | C=1380; O=15; E=2.83; R=5.29; rawP=5.18e-08; adjP=4.52e-06 |  |  |  |
|  | regulation of cellular component organization, GO:0051128 | C=1200; O=14; E=2.47; R=5.68; rawP=6.85e-08; adjP=5.04e-06 |  |  |  |
|  | regulation of biological quality, GO:0065008 | C=2136; O=18; E=4.39; R=4.10; rawP=7.69e-08; adjP=5.04e-06 |  |  |  |
|  | organic substance transport, GO:0071702 | C=1684; O=16; E=3.46; R=4.62; rawP=1.05e-07; adjP=5.50e-06 |  |  |  |
|  | cellular protein complex assembly, GO:0043623 | C=283; O=8; E=0.58; R=13.76; rawP=1.00e-07; adjP=5.50e-06 |  |  |  |
|  | cytoskeletal protein binding, GO:0008092 | C=603; O=9; E=1.25; R=7.21; rawP=3.32e-06; adjP=9.96e-05 |  |  |  |
|  | :RNA binding, GO:0003723 | C=744; O=9; E=1.54; R=5.84; rawP=1.80e-05; adjP=0.0003 |  |  |  |
|  | translation factor activity, nucleic acid binding, GO:0008135 | C=88; O=4; E=0.18; R=21.96; rawP=3.29e-05; adjP=0.0005 |  |  |  |
|  | :actin binding,  GO:0003779 | C=322; O=6; E=0.67; R=9.00; rawP=5.04e-05; adjP=0.0006 |  |  |  |
|  | endopeptidase activator activity, GO:0061133 | C=6; O=2; E=0.01; R=161.04; rawP=6.25e-05; adjP=0.0007 |  |  |  |
|  | non-membrane-bounded organelle, GO:0043228 | C=2869; O=21; E=5.96; R=3.53; rawP=5.65e-08; adjP=6.04e-07 |  |  |  |
|  | intracellular non-membrane-bounded organelle, GO:0043232 | C=2869; O=21; E=5.96; R=3.53; rawP=5.65e-08; adjP=6.04e-07 |  |  |  |
|  | intracellular organelle, GO:0043229 | C=9656; O=38; E=20.05; R=1.90; rawP=6.03e-08; adjP=6.04e-07 |  |  |  |
| **Cell modification Leading genes obob alb-SREBP-1c Gene Symbol** | **Database biological process/ molecular function name** | **stat** | **metabolism**  **Leading genes obob alb-SREBP-1c** | **Database biological process/ molecular function name** | **stat** |
| Sucla2, **Ywhaz**, **Hspd1**, Spr, Acot2, Acaca, **Ckb**, Aldh1l1, Idh3a, Glud1, Atp6v1a, Lap3, Aldoc, **Pebp1**, Mdh2, Pkm, Nme2, Asl, Psma6, Amacr, Anxa1, Dlat, Aco2, **Hebp1**, Sdha, Nln, Isoc2a, Sod2, Hk2, Gsta4, Iars2, Cycs, Nrd1, Glul, Fdps, Me1, Oxct1, Cav1, Aldoa, Mut, Adh5, Lipe, Nme1, Hspa9, Plec, Vapa, Copa, Psmb1, Psma5, Csrp1, Copz1, H2afy, **Abhd14b**, Vps26a, Stard10, Hnrnpk, Arcn1, Taldo1, Copb2, Ehd2, Hip1, Ehd1, Aldob, Stat1, Hnrnpa1, Uso1, Nampt, Vil1, Sncg, Nsfl1c, Grb2, S100a10, Krt8, Adk, Nap1l1, Ap2a1, Ada, **Cope**, Rpl5, Nsdhl, Rps3, Rdx, Myh11, Snx5, Ap3b1, **Tpi1**, Psma4, Capg, Copb1, Hnrnpd, Psmc5, **Capza**1, **Iqgap1**, Cryz, Sgce, Pa2g4, Pgd, **Psma1**, Rtn3, Rtn4, Tpp2, Mvp, Tln2, Slc9a3r1, Pls3, Myo1c, Tkt, Ptpn6, Psmb3, Hnrnpc, Got1 | mitochondrion; GO:0005739 | C=1576; O=44; E=10.10; R=4.36; rawP=1.95e-17; adjP=5.10e-16 | Arg1, Pck1, Sucla2, Spr, Nsdhl, Acot2, Acaca, Dak, Aldh1l1, Idh3a, Glud1, Atp6v1a, Fabp5, Mdh2, Akr1b8, Pkm, **Tpi1**, Nme2, Asl, Csad, Amacr, Aacs, Anxa1, Dlat, Aco2, Sdha, Taldo1, Psmc5, Abhd5, Sod2, Psat1, Gsta4, Iars2, Aldob, Glul, Pgd, Fdps, Me1, Oxct1, Cav1, Nampt, Aldoa, Mut, Grhpr, Adh5, Lipe, Tkt, Adk, Got1, Nme1, Ada, Pygl, Serpinb1a, Psmb1, Ywhah, Psma5, Aldoc, Plin1, Pygb, Psma6, Psma4, Hk2, Cycs, Nrd1, **Psma1**, Psmb3, **Hspd1**, **Ckb**, H2afy, Ywhaq, Hnrnpk, Galm, Isoc2a, Hip1, Stat1, Hnrnpa1, Vil1, Grb2, Nap1l1, **Cndp2**, Hspa9, Ptgr1, Rpl5, Rps3, Gpt, **Eno1**, Lap3, **Pebp1**, Myh11, Gsta3, **Ppa1**, **Hebp1**, Nln, Hnrnpd, Eef1g, I**qgap1**, Cryz, Pa2g4, Tpp2, Slc9a3r1, Gstm5, Ptpn6, Hnrnpc, Ehd2, Ehd1, Myo1c, **Ywhaz**, Plec, Anxa6, Vapa, Copa, Csrp1, Vps26a, Copb2, Uso1, Sncg, Nsfl1c, Pdzk1, S100a10, Krt8, Ap2a1, **Anxa5**, Rdx, Snx5, Ap3b1, **Capza1**, Sgce, Rtn3, Rtn4, Tln2, Pls3 | small molecule metabolic process; GO:0044281 | C=1821; O=51; E=11.47; R=4.45; rawP=6.66e-21; adjP=7.60e-18 |
|  | intracellular organelle; GO:0043229 | C=9656; O=111; E=61.88; R=1.79; rawP=3.39e-17; adjP=7.75e-16 |  | carboxylic acid metabolic process; GO:0019752 | C=675; O=31; E=4.25; R=7.29; rawP=2.23e-18; adjP=1.27e-15 |
|  |  |  |  | oxoacid metabolic process; GO:0043436 | C=717; O=31; E=4.52; R=6.86; rawP=1.23e-17; adjP=4.68e-15 |
|  |  |  |  | organic acid metabolic process; GO:0006082 | C=731; O=31; E=4.61; R=6.73; rawP=2.12e-17; adjP=6.05e-15 |
|  |  |  |  | organic substance catabolic process; GO:1901575 | C=1515; O=41; E=9.54; R=4.30; rawP=4.77e-16; adjP=9.07e-14 |
|  |  |  |  | metabolic process; GO:0008152 | C=8894; O=103; E=56.03; R=1.84; rawP=4.11e-16; adjP=9.07e-14 |
|  |  |  |  | catabolic process; GO:0009056 | C=1645; O=42; E=10.36; R=4.05; rawP=1.45e-15; adjP=2.36e-13 |
|  |  |  |  | dicarboxylic acid metabolic process; GO:0043648 | C=69; O=12; E=0.43; R=27.61; rawP=1.56e-14; adjP=2.22e-12 |
|  |  |  |  | single-organism metabolic process; GO:0044710 | C=8262; O=96; E=52.05; R=1.84; rawP=2.86e-14; adjP=3.63e-12 |
|  |  |  |  | organic substance metabolic process; GO:0071704 | C=7922; O=93; E=49.91; R=1.86; rawP=8.00e-14; adjP=9.13e-12 |
|  |  |  |  | catalytic activity; GO:0003824 | C=5105; O=81; E=32.64; R=2.48; rawP=1.29e-18; adjP=1.26e-16 |
|  |  |  |  | carboxylic acid binding; GO:0031406 | C=189; O=14; E=1.21; R=11.59; rawP=1.97e-11; adjP=7.68e-10 |
|  |  |  |  | lyase activity; GO:0016829 | C=157; O=12; E=1.00; R=11.95; rawP=3.98e-10; adjP=1.29e-08 |
|  |  |  |  | cofactor binding; GO:0048037 | C=254; O=14; E=1.62; R=8.62; rawP=9.88e-10; adjP=2.75e-08 |
|  |  |  |  | binding; GO:0005488 | C=10781; O=119; E=68.93; R=1.73; rawP=4.25e-19; adjP=8.29e-17 |
|  |  |  |  | protein binding; GO:0005515 | C=6181; O=80; E=39.52; R=2.02; rawP=6.08e-13; adjP=3.95e-11 |
|  |  |  |  | small molecule binding; GO:0036094 | C=2437; O=46; E=15.58; R=2.95; rawP=4.89e-12; adjP=2.38e-10 |
|  |  |  |  | threonine-type endopeptidase activity; GO:0004298 | C=21; O=6; E=0.13; R=44.69; rawP=3.07e-09; adjP=6.65e-08 |
|  |  |  |  | threonine-type peptidase activity; GO:0070003 | C=21; O=6; E=0.13; R=44.69; rawP=3.07e-09; adjP=6.65e-08 |
|  |  |  |  | anion binding; GO:0043168 | C=2256; O=39; E=14.42; R=2.70; rawP=4.51e-09; adjP=8.79e-08 |
| **Leading genes C57Bl6, obob Gene Symbol** | **Database biological process/ molecular function name** | **stat** | **metabolism , Leading genes C57Bl6, obob** | **Database biological process/ molecular function name** | **stat** |
| Copb2, Akap12, Ap2a2, Sorbs1, Sec31a, Naca, Ap2m1, Pdcd6ip, Eif5a, Copz1, Vps29, Uso1, Picalm, Vps35, Ywhaq, Ap3b1, Arcn1, Prkaca, Copb1, Anxa1, Ptpn11, Vps26b, Hspa9, Syncrip, Rpl5, Anxa6, Capza2, Arpc2, Mapre1, Ndufs1, Set, Aldh1l1, Atp6v1a, Myl12b, H2afy, Eif4g1, Ncl, Eif2a, Psme1, Eif2s1, Psma4, Tuba1b, Dlat, Dctn1, Eif3c, Psmc5, Eef1g, **Capza1**, Txnl1, **Iqgap1**, Fermt2, Psmd2, Sgce, Pa2g4, Rtn4, Cct6a, Mvp, Aldoa, Clic1, Grb2, Ptpn6, Actr3, Krt8, Eef2, Psmd11, UserID, Plec, Oplah, Cat, Actr2, Fam129b, Asl, Aacs, Sod2, Psat1, Fscn1, Hip1, Cycs, Lgals1, Stat1, Me1, Tln1, Txndc17, Vil1, Mut, **Anxa3**, Sncg, Grhpr, S100a10, Sucla2, Nsdhl, Anxa5, Pygl, Acot2, Idh3a, Ddt, **Pebp1**, Fabp5, Akr1b8, Pkm, **Tpi1**, Csad, Pygb, Capg, **Hebp1**, Gsta4, Pgd, Rtn3, Tln2, Cap1, Got1, Bcat2, **Abhd14b**, Samhd1, Lap3, Tpp2 | establishment of protein localization; GO:0045184 | C=1080; O=23; E=6.06; R=3.79; rawP=3.07e-08; adjP=2.76e-06 | Sucla2, Nsdhl, Acot2, Oplah, Ndufs1, Aldh1l1, Idh3a, Atp6v1a, Cat, Fabp5, Akr1b8, Pkm, **Tpi1**, Asl, Csad, Aacs, Tuba1b, Dlat, Anxa1, Akap12, Psmc5, Sod2, Psat1, Txnl1, Gsta4, Eif5a, Pgd, Me1, Aldoa, Mut, Grhpr, Got1, Eef2, Bcat2, Sorbs1, Pygl, Cycs, Pygb, Prkaca, Syncrip, Ap2a2, Anxa6, Plec, Arpc2, Mapre1, Set, Actr2, H2afy, Eif4g1, Fam129b, Samhd1, Ywhaq, Galm, Dctn1, Copb2, Fscn1, Naca, Hip1, Psmd2, Lgals1, Stat1, Uso1, Hspa12a, Tln1, Vps35, Vil1, **Anxa3**, Sncg, Grb2, S100a10, Krt8, Psme2, Ptpn11, Hspa9, Rpl5, Capza2, **Anxa5**, Pdcd6ip, Gpt, Myl12b, Lap3, Vps29, Picalm, **Pebp1**, Plin1, Ncl, Ap3b1, Eif2a, Psme1, Eif2s1, **Hebp1**, Eif3c, Eef1g, **Capza1**, **Iqgap1**, Sec31a, Fermt2, Ap2m1, Sgce, Pa2g4, Rtn3, Rtn4, Cct6a, Tln2, Tpp2, Cap1, Gstm5, Efhd2, Ptpn6, Actr3 | small molecule metabolic process; GO:0044281 | C=1821; O=34; E=10.23; R=3.33; rawP=2.26e-10; adjP=6.78e-08 |
|  | macromolecular complex; GO:0032991 | C=3542; O=57; E=20.14; R=2.83; rawP=8.68e-15; adjP=1.95e-13 |  | binding; GO:0005488 | C=10781; O=104; E=61.49; R=1.69; rawP=1.46e-15; adjP=2.61e-13 |
|  | protein complex; GO:0043234 | C=2978; O=51; E=16.93; R=3.01; rawP=4.46e-14; adjP=8.75e-13 |  | protein binding; GO:0005515 | C=6181; O=68; E=35.25; R=1.93; rawP=5.85e-10; adjP=5.24e-08 |
|  | cellular process; GO:0009987 | C=12976; O=103; E=72.86; R=1.41; rawP=3.86e-09; adjP=5.79e-07 |  | pyridoxal phosphate binding; GO:0030170 | C=51; O=6; E=0.29; R=20.63; rawP=4.45e-07; adjP=1.33e-05 |
|  | cellular component biogenesis; GO:0044085 | C=1465; O=28; E=8.23; R=3.40; rawP=7.81e-09; adjP=1.00e-06 |  | translation factor activity, nucleic acid binding; GO:0008135 | C=88; O=7; E=0.50; R=13.95; rawP=7.17e-07; adjP=1.83e-05 |
|  | cellular component assembly; GO:0022607 | C=1335; O=26; E=7.50; R=3.47; rawP=2.05e-08; adjP=2.05e-06 |  | carboxylic acid metabolic process; GO:0019752 | C=675; O=21; E=3.79; R=5.54; rawP=1.75e-10; adjP=6.78e-08 |
|  | actin binding; GO:0003779 | C=322; O=12; E=1.84; R=6.53; rawP=3.29e-07; adjP=1.33e-05 |  | generation of precursor metabolites and energy; GO:0006091 | C=291; O=15; E=1.63; R=9.18; rawP=9.58e-11; adjP=6.78e-08 |
|  | cytoskeletal protein binding; GO:0008092 | C=603; O=15; E=3.44; R=4.36; rawP=1.83e-06; adjP=4.09e-05 |  | oxoacid metabolic process; GO:0043436 | C=717; O=21; E=4.03; R=5.22; rawP=5.24e-10; adjP=1.18e-07 |
|  | ribosome binding; GO:0043022 | C=26; O=4; E=0.15; R=26.97; rawP=1.37e-05; adjP=0.0002 |  | organic acid metabolic process; GO:0006082 | C=731; O=21; E=4.10; R=5.12; rawP=7.43e-10; adjP=1.34e-07 |
|  | intracellular organelle; GO:0043229 | C=9656; O=92; E=54.91; R=1.68; rawP=1.81e-11; adjP=3.16e-10 |  | vitamin B6 binding; GO:0070279 | C=51; O=6; E=0.29; R=20.63; rawP=4.45e-07; adjP=1.33e-05 |
|  | organelle; GO:0043226 | C=9679; O=92; E=55.04; R=1.67; rawP=2.13e-11; adjP=3.34e-10 |  | cofactor binding; GO:0048037 | C=254; O=11; E=1.45; R=7.59; rawP=2.36e-07; adjP=1.33e-05 |

____________________________________________________________________________________________________________________
