# Supplementary material for: Adipokinome Signatures in Obese Mouse Models Reflect Adipose Tissue Health and Are Associated with Serum Lipid Composition
Source: Int J Mol Sci. 2019 May 24;20(10):2559. doi: 10.3390/ijms20102559 (PMC6567124; doi:10.3390/ijms20102559)
Supplement: Supplementary file 1 [file ijms-20-02559-s001.zip › IJMS Table S6A.docx]

**Supplement Table 6A:** **Abundance of FA metabolism related proteins in adipokines correlated to liver-to-adipose-tissue DNL-ratio.**

Proteins with correlation to DNL-ratio were subjected to IPA^®^ Core analyses. Upstream regulating molecules or master regulators of signaling networks involved in fibrosis were screened for genes influenced by cC16:1 or cC18:1 (Ingenuity®, Qiagen).

| **Master Regulator/ Upstream Regulator** | **p-value of overlap** | **Participating regulators** | **Target molecules in dataset** |
| --- | --- | --- | --- |
| **C57Bl6** |  |  |  |
| ADIPOQ | 4.48E-07 | ADIPOQ, AMPK, CREB1, CTNNB1, estrogen receptor, IRS1, Jnk, MKNK1, MTOR, NFE2L2, NFkB (complex), NLRP3, P38 MAPK, PGR, PPARG, RELA, RHOA, TCF7L2, TP53 | ABAT, AKR1A1, ATL3, CYP2E1, DPYSL3, EPB41L2, GSR, GSTM1, GSTM3, KRT8, LAP3, LDHB, Otub1, PDXK, PICALM, PLA2G7, PLIN1, PSMD2, RPL12, SERPIND1, THOP1, UBE2N, YWHAH |
| AGPAT2 | 3.96E-02 | AGPAT2 | PLIN1 |
| AKT1 | 2.52E-08 | AKT1, AR, ATF6, BCL10, BRCA1, EGFR, ERK1/2, FOXO1, HMOX1, INPP5D, MAPK1, MKNK1, N-cor, NFkB (complex), NLRP3, P38 MAPK, PGR, REL, TP53, TP63, TP73, TSC2 | PLIN1 |
| ATF6 | 8.57E-07 | Akt, ATF6, ERK1/2, estrogen receptor, IKBKB, MTOR, NFkB (complex), NLRP3, P38 MAPK, RB1, TFEB, TP53, TSC2 | ABAT, ACAD9, ATL3, ATP6V1B2, CALR, COTL1, DPYSL3, EPB41L2, GDI1, GSR, GSTM1, GSTM3, KRT8, LAP3, LCP1, PCBP2, PDIA4, PDIA6, PLA2G7, PLIN1, PLS3, PSMD2, THOP1, UBE2N, VIL1, YWHAH |
| ATM | 3.54E-04 | ATM, CREB1, IKBKB, IKBKG, MAPK1, TP53, TRIM28 | ABAT, AKR1A1, ATL3, ATP6V1B2, CALR, DLAT, GM2A, GSR, KRT8, LDHB, Otub1, PDIA4, PDIA6, PLA2G7, PSMD2, RPL12, THOP1, UBE2N, YWHAH |
| CD36 | 7.22E-07 | Akt, ATF6, BAK1, BAX, CAV1, CD36, CFTR, ERK1/2, ERN1, MKNK1, MMP9, MTOR, P38 MAPK, PGR, Pka, PPARG, RB1, RHO, TGFB1, TP63, TSC2 | ABAT, ALAD, ATL3, GM2A, GSR, KRT8, LAP3, PDIA6, PDXK, PSMD2, THOP1, YWHAH |
| CD44 | 3.47E-06 | CASP8, CAV1, CD44, CREB1, CTNNB1, EGFR, ESR1, MAPK3, MKNK1, NFE2L2, NLRP3, P38 MAPK, PPARG, PRKCE, REL, SRC, SYK, TCF7L2, TGFB1, TGM2, TP53, TP63, TRAF2 | AKR1A1, ATP6V1B2, CALR, CRABP1, CYP2E1, DLAT, DPYSL3, EPB41L2, GSR, GSTM3, Otub1, PDIA4, PDIA6, PLA2G7, PLIN1, PTRF, RPL12, SELENBP1, VIL1 |
| CD74 | 3.99E-05 | Akt, ATF6, CD74, IKBKB, MKNK1, MTOR, NFkB (complex), NLRP3, P38 MAPK, PGR, PPARG, REL, SYK, TP63, TSC2 | ABAT, ACAD9, ATL3, COTL1, CYP2E1, DPYSL3, EPB41L2, GSR, GSTM1, NLN, PDXK, PICALM, PLA2G7, PLIN1, PSMD2, PTRF, PYGL, SELENBP1, SERPIND1, THOP1, UBE2N, VIL1, YWHAH |
| CETP | 4.19E-02 | CETP | ACAD9, AKR1A1, ATP6V1B2, COTL1, DPYSL3, EPB41L2, GM2A, GSTM3, LDHB, Otub1, PDIA4, PLA2G7, PLIN1, RPL12, UBE2N, VIL1 |
| ERN1 | 3.37E-06 | CHUK, CTNNB1, ERK1/2, ERN1, estrogen receptor, GSK3B, IKBKB, Jnk, MTOR, MYC, NFE2L2, NFkB (complex), NLRP3, P38 MAPK, TFEB, TP53 | PLIN1 |
| Esrra | 6.13E-05 | AMPK, ERK1/2, ERN1, Esrra, MAPK1, P38 MAPK, PRKAA1, PRKAA2, RELA, TP53 | PLIN1 |
| Esrra | 1.41E-02 | Esrra | ABAT, AKR1A1, ATL3, ATP6V1B2, CALR, CYP2E1, GM2A, GSTM1, KRT8, LAP3, LDHB, Otub1, PDIA4, PLA2G7, PSMD2, RPL12, SERPIND1, THOP1, UBE2N, YWHAH |
| FABP5 | 8.76E-05 | Akt, ERK, FABP5, MTOR, NFE2L2, NOS2, P38 MAPK, PPARG, TSC2 | ABAT, ATL3, CALR, CYP2E1, DLAT, GSR, KRT8, LAP3, LDHB, PDIA6, PLA2G7, PSMD2, PYGL, THOP1, YWHAH |
| FBXW7 | 8.01E-06 | AR, ERK1/2, Esrra, FBXW7, MAPK1, MTOR, MYC, NCOA3, NOTCH1, PPARGC1A, TP53 | DLAT, PYGL |
| FGF21 | 6.48E-05 | CREB1, ERK1/2, FGF21, IKBKB, MAPK1, NFE2L2, P38 MAPK, Pkc(s), PPARG, PRKCE, PRKCQ, TRAF2 | DLAT, PYGL |
| FOXO4 | 3.97E-05 | 26s Proteasome, Akt, AMPK, BAX, CDKN1A, FOXO4, MAPK1, P38 MAPK, PPARG, SIRT1, TP53, TSC2, XBP1 | AKR1A1, ATP6V1B2, CYP2E1, DLAT, GSR, GSTM1, LDHB, Otub1, PDIA4, PDIA6, PLA2G7, PLIN1, RPL12, SERPIND1 |
| GH1 | 3.77E-02 | GH1 | ABAT, AKR1A1, ATL3, CALR, DLAT, GDI1, GSR, GSTM1, KRT8, LAP3, LDHB, Otub1, PDIA6, PLS3, PSMD2, PYGL, RPL12, THOP1, YWHAH |
| Ins1 | 4.01E-05 | AKT1, CHUK, IKBKB, Ins1, Mapk, MKNK1, N-cor, NOS2, P38 MAPK, PI3K (complex), RB1, TP53, TSC2 | AKR1A1, CALR, DLAT, GM2A, GSR, GSTM1, KRT8, LAP3, PDIA4, PDIA6, PDXK, PLA2G7, PLIN1, PYGL, SERPIND1 |
| IRS1 | 1.63E-06 | AR, ATF6, BAK1, BAX, CHUK, CREB1, ERK1/2, FMR1, FOXO1, IKBKB, IRS1, MAP2K1/2, Mapk, MAPK1, MKNK1, MTOR, NOS2, P38 MAPK, PGR, PI3K (complex), PIK3R1, PPARG, RICTOR, RPS6KB1, TP63 | ABAT, ATL3, ATP6V1B2, CYP2E1, GSR, KRT8, LAP3, LDHB, PDIA4, PDIA6, PLA2G7, PLIN1, PSMD2, Rrbp1, THOP1, YWHAH |
| IRS2 | 2.32E-06 | AKT1, AR, ATF6, CREB1, CTNNB1, ERK1/2, FOXO1, GSK3B, HOXA10, IRS2, JAK2, MKNK1, MTOR, NOS2, P38 MAPK, PGR, PI3K (complex), PIK3R1, TP53 | AKR1A1, CYP2E1 |
| LIPE | 1.60E-06 | AKT1, ATF6, CAV1, CHUK, DGAT1, ERK1/2, H6PD, IKBKB, LIPE, MTOR, N-cor, NOS2, P38 MAPK, PGR, PI3K (complex), RB1, SRC (family), TP53, TSC2 | ABAT, ATL3, ATP6V1B2, CYP2E1, DLAT, DPYSL3, EPB41L2, GM2A, GSR, KRT8, PDIA6, PLA2G7, PLIN1, PSMD2, THOP1, YWHAH |
| MIF | 3.01E-10 | Akt, CAV1, CREB1, CTNNB1, E2f, ERK1/2, HRAS, IKBKB, MAPK1, MIF, MTOR, NFE2L2, NFkB (complex), NLRP3, PGR, PRKCD, REL, SRC, SYK, TP53, TP73, TRAF2, TSC2 | AKR1A1, CALR, CYP2E1, DAG1, DPYSL3, EPB41L2, GDI1, GM2A, GSTM1, GSTM3, LAP3, Otub1, PDIA4, PDIA6, PDXK, PLA2G7, PLIN1, PLS3, PSMD2, RPL12, VIL1 |
| NOS2 | 3.20E-08 | Akt, AR, BAX, CHUK, CREB1, estrogen receptor, FOXO1, GSK3B, IKBKB, IRS1, IRS2, ITGB2, Jnk, MMP9, MTOR, NFE2L2, NFkB (complex), NLRP3, NOS2, P38 MAPK, TFEB, TGFB1, TP53, TRAF2, TSC2 | ABAT, AKR1A1, ATL3, CALR, CYP2E1, DPYSL3, EPB41L2, GDI1, GSR, GSTM1, GSTM3, KRT8, Otub1, PDIA4, PDXK, PLA2G7, PLS3, PSMD2, RPL12, THOP1, YWHAH |
| NR1I2 | 6.26E-07 | BAK1, BAX, CD36, ERK1/2, IKBKB, MKNK1, MTOR, NR1I2, P38 MAPK, PPARG, TP63 | ABAT, AKR1A1, ATL3, ATP6V1B2, CALR, CYP2E1, DLAT, GM2A, GSR, GSTM3, Otub1, PDIA4, PDIA6, PLA2G7, PLIN1, PSMD2, PTRF, RPL12, THOP1, YWHAH |
| NR1I2 | 1.46E-04 | NR1I2 | ABAT, ACAD9, AKR1A1, ATL3, ATP6V1B2, CALR, COTL1, CYP2E1, GM2A, GSR, GSTM1, GSTM3, KRT8, LAP3, LCP1, LDHB, Otub1, PDIA4, PDIA6, PDXK, PSMD2, PTRF, PYGL, RPL12, SERPIND1, THOP1, UBE2N, YWHAH |
| NR1I2 | 2.44E-04 | NR1I2 | ABAT, AKR1A1, ATL3, ATP6V1B2, CYP2E1, GDI1, GM2A, GSTM1, KRT8, LDHB, Otub1, PDIA4, PDIA6, PDXK, PLA2G7, PLS3, PSMD2, PYGL, RPL12, SELENBP1, SERPIND1, THOP1, UBE2N, YWHAH |
| PPARA | 1.85E-03 | PPARA | AKR1A1, CALR, DLAT, DPYSL3, EPB41L2, GM2A, GSTM1, GSTM3, LDHB, Otub1, PDIA4, PDIA6, PLA2G7, PLIN1, RPL12, SERPIND1, VIL1 |
| PPARD | 3.18E-07 | AKT1, AR, BAK1, BAX, CASP3, CREB1, CTNNB1, ERK1/2, FOXO1, GSK3B, MAP2K1/2, MKNK1, MTOR, Pkc(s), PPARD, PRKAA, PRKCA, RAF1, STAT3, TCF7L2, TP53, TP63 | GSTM1, GSTM3, PDIA4, SERPIND1 |
| PPARG | 8.83E-07 | AKT1, APP, Creb, CREB1, CTNNB1, IRS1, MKNK1, MMP9, MTOR, NFE2L2, NFkB (complex), NFKBIA, NLRP3, P38 MAPK, PGR, PPARG, STAT3, TGFB1, TP63, TP73, TSC2 | GSTM1, GSTM3, PDIA4, SERPIND1 |
| PPARG | 1.16E-02 | PPARG | GSTM3, KRT8, PLA2G7, PLIN1, SELENBP1 |
| PTEN | 3.27E-04 | CREB1, CTNNB1, ERK1/2, estrogen receptor, MTOR, P38 MAPK, PTEN, TSC2 | ABAT, AKR1A1, ATL3, CALR, CYP2E1, DPYSL3, EPB41L2, GDI1, GSR, GSTM1, KRT8, LAP3, LDHB, Otub1, PDIA6, PDXK, PICALM, PLS3, PSMD2, RPL12, THOP1, VIL1, YWHAH |
| SIRT1 | 2.07E-06 | Akt, AMPK, CHUK, IKBKB, NFE2L2, NFkB (complex), NFKB1, NLRP3, PGR, PI3K (complex), PPARG, SERPINE1, SIRT1, TGFB1, TP53, TP73, TSC2, XBP1 | AKR1A1, ATP6V1B2, CYP2E1, DLAT, DPYSL3, EPB41L2, GSR, GSTM1, GSTM3, LCP1, Otub1, PDIA4, PDIA6, PDXK, PLA2G7, PLIN1, RPL12, SELENBP1, SERPIND1, UBE2N, VIL1 |
| SIRT1 | 5.06E-05 | AMPK, PPARG, SIRT1, TP53, XBP1 | DLAT, HEBP1, PLIN1, PYGL |
| SREBF1 | 4.66E-02 |  | AKR1A1, ATP6V1B2, CALR, CYP2E1, DLAT, KRT8, LAP3, LDHB, Otub1, PDXK, PLA2G7, RPL12 |
| TNFSF10 | 3.81E-08 | Akt, AMPK, BAK1, BAX, CASP8, CAV1, CREB1, ERK1/2, ERN1, estrogen receptor, IKBKB, Jnk, MAP3K7, MAPK1, MKNK1, MTOR, NFkB (complex), NLRP3, P38 MAPK, PGR, Pkc(s), PPARG, PRKCE, RELA, SRC, TAB2, TNFSF10, TP53, TP63, TRAF2, TSC2 | DLAT, LDHB, VIL1 |
| USP7 | 7.50E-03 | AR, PPARG, USP7 | ABAT, ATL3, ATP6V1B2, CYP2E1, DLAT, GM2A, GSR, GSTM1, GSTM3, KRT8, LCP1, LDHB, PDIA4, PDIA6, PLIN1, PSMD2, Rrbp1, SELENBP1, SERPIND1, THOP1, UBE2N, YWHAH |
| **Alb-SREBP-1c** |  |  |  |
| **Master Regulator** | **p-value of overlap** | **Participating regulators** | **Target molecules in dataset** |
| ABHD5 | 2.09E-05 | ABHD5, AMPK, MTORC1 | FDPS, IDH3A, LDHB, PKM |
| ADIPOQ | 2.72E-07 | ADIPOQ, Akt, AMPK, CTNNB1, estrogen receptor, IGF1R, Jnk, MKNK1, MTOR, MYC, NFE2L2, NFkB (complex), NOS2, P38 MAPK, PI3K (complex), PRKAA1, PRKAA2, RELA, RHOA, STK11, TCF7L2, TP53, USP8 | ACACA, ACTC1, ACTN4, ECH1, EEF1D, FBP2, FDPS, GSTM1, HNRNPD, IDH3A, ITIH4, ITIH5, LDHB, MAPK14, MSN, PAFAH1B2, PKM, RPLP0, SERPINH1, SNX5, SORBS1, TOP2B |
| ADIPOR1 | 2.31E-04 | ADIPOR1, AMPK, PRKAA2 | IDH3A, ITIH5, LDHB, TGM2 |
| AGPAT2 | 1.19E-03 | AGPAT2, ERK1/2, MTORC1, Pld | ACACA, FDPS, ICAM1, PKM |
| CCKAR | 3.40E-06 | Akt, CCKAR, PI3K (family), RB1, SREBF1, SREBF2, STAT1, TP53, TSC2, USP8 | ACACA, ACTC1, ACTN4, ECH1, FDPS, HNRNPD, LDHB, PAFAH1B2, PGD, PKM, PSME1, SERPINH1, SNX5, SORBS1, TOP2B, Tpm1 |
| CCKBR | 5.59E-07 | ADCY, Akt, AMPK, CCKBR, HOXA10, JAK2, PI3K (family), RB1, SREBF1, SREBF2, STAT3, TP53, USP8 | ACACA, ACTC1, ACTN4, COL15A1, ECH1, FDPS, HNRNPD, ICAM1, IDH3A, PAFAH1B2, PGD, PKM, SERPINH1, SNX5, SORBS1, TOP2B, Tpm1 |
| CD44 | 1.76E-08 | CD44, CTNNB1, EGFR, ERK, ESR1, ESR2, FAS, GSK3B, INSR, ITGB2, MAPK1, MAPK3, MKNK1, MYC, NFE2L2, NOS2, P38 MAPK, PRKAA1, PRKCE, PSEN1, Rac, REL, SRC, STAT1, SYK, TCF7L2, TGFB1, TGM2, TP53 | ACTC1, ACTN4, COL15A1, CTSB, ECH1, EEF1D, FBLN5, FBP2, GNB1, GSTM1, HNRNPD, IDH3A, ITIH4, ITIH5, NQO2, PAFAH1B2, PLEC, POR, PSME1, RPLP0, SERPINH1, SNX5, SORBS1, TGM2, TOP2B |
| CEBPB | 3.21E-06 | ACLY, ATM, CASP1, CEBPB, INSR, NFkB (complex), PI3K (complex), TP53 | ACACA, ACTN4, CTSB, ECH1, FDPS, ICAM1, IDH3A, PAFAH1B2, SERPINH1, SNX5, SORBS1, TGM2, TOP2B, Tpm1 |
| CYP7A1 | 3.51E-04 | CYP7A1 | ACACA, FDPS |
| ERN1 | 2.75E-06 | CEBPA, CTNNB1, ERN1, estrogen receptor, GSK3B, IGF1R, Jnk, MTOR, MTORC1, MYC, NFE2L2, NFkB (complex), PSEN1, STAT1, TP53 | ACACA, ACTC1, ACTN4, ECH1, FDPS, GNB1, GSTM1, ICAM1, LDHB, MAPK14, MSN, PAFAH1B2, PGD, PSME1, RPLP0, SNX5, SORBS1, TGM2, TOP2B |
| ESRRA | 4.27E-02 | ESRRA | LDHB, PKM |
| ESRRA | 1.49E-02 | ESRRA | ECH1, IDH3A |
| FBXW7 | 3.11E-07 | AR, ESR1, FBXW7, MTOR, MYC, NCOA3, NOTCH1, PPARGC1A, RB1, SRC, TP53 | ACACA, ACTC1, ACTN4, COL15A1, ECH1, FDPS, GSTM1, HNRNPD, ICAM1, IDH3A, LDHB, NQO2, PAFAH1B2, PGD, PKM, SERPINH1, SNX5, SORBS1, TOP2B, Tpm1 |
| GH1 | 3.17E-02 | GH1 | ACACA, FDPS |
| INSIG2 | 4.71E-04 | INSIG2 | ACACA, FDPS |
| INSR | 9.52E-06 | INSR | ACTN4, ALAD, CTSB, FDPS, GNB1, IDH3A, IQGAP1 |
| Irs4 | 4.39E-06 | AKT1, AMPK, ERK1/2, IRS1, IRS2, Irs4, NOS2, PI3K (complex), PIK3R1, PRKAA1, RB1, STAT1, TP53 | ACACA, ACTC1, ACTN4, ECH1, ICAM1, IDH3A, ITIH4, ITIH5, PAFAH1B2, PGD, PKM, PSME1, SERPINH1, SNX5, SORBS1, TGM2, TOP2B |
| LEP | 1.71E-06 | AHR, AMPK, Creb, CTNNB1, ESR1, ESR2, GSK3B, Jnk, LEP, Mapk, MAPK14, MKNK1, MTOR, NFE2L2, NOS2, PIK3R1, PRKAA2, PSEN1, PTEN, RICTOR, RPS6KB1 | ACACA, ACTC1, CTSB, ECH1, EEF1D, FBLN5, FBP2, GNB1, GSTM1, HNRNPD, ICAM1, ITIH4, ITIH5, LDHB, PKM, PLEC, PSMC5, RPLP0, SERPINH1, SUCLG2, Tpm1 |
| LEP | 1.30E-03 | LEP | ACACA, ECH1, FBP2, ICAM1 |
| LIPE | 1.34E-06 | ACSL1, AKT1, AMPK, DGAT1, ERK1/2, ESR1, LIPE, MTOR, NOS2, PGR, PI3K (complex), PRKAA1, RB1, SRC (family), STAT1, TP53 | ACTC1, ACTN4, ECH1, FBP2, HNRNPD, ICAM1, IDH3A, ITIH4, ITIH5, NQO2, PAFAH1B2, PGD, PKM, PSME1, SERPINH1, SNX5, SORBS1, TGM2, TOP2B |
| LPIN1 | 4.27E-06 | CEBPA, ERK1/2, HIF1A, INSR, Jnk, JUN, LPIN1, MKNK1, MTOR, MTORC1, NFE2L2, P38 MAPK, PGR, Pkc(s), PPARA, PRKAA1, PRKCE, RB1, SRC, STAT1 | ACACA, ACTC1, CTSB, ECH1, EEF1D, FDPS, GSTM1, ICAM1, IDH3A, ITIH5, LDHB, NQO2, PGD, PKM, PSME1, RPLP0, SERPINH1, TGM2 |
| LPIN1 | 7.63E-04 | LPIN1 | ACACA, FDPS |
| NOS2 | 1.81E-08 | Akt, AR, estrogen receptor, FOXO1, GSK3B, IGF1R, IRS1, IRS2, Jnk, MMP9, MTOR, NFE2L2, NFkB (complex), NOS2, PSEN1, Rap1, SRC, TGFB1, TP53, USP8 | ACACA, ACTC1, ACTN4, ECH1, FBLN5, FDPS, GNB1, GSTM1, HNRNPD, ICAM1, ITIH4, LDHB, MAPK14, MSN, NQO2, PAFAH1B2, PKM, RPLP0, SERPINH1, SNX5, SORBS1, TGM2, TOP2B |
| NOS2 | 5.70E-05 | IRS1, IRS2, Jnk, MMP9, MTOR, NFkB (complex), NOS2 | ACACA, ACTC1, FDPS, ICAM1, ITIH4, LDHB, PKM, SERPINH1, TGM2 |
| NR1H2 | 1.30E-02 | NR1H2 | ACACA, FDPS |
| NR1H3 | 1.36E-02 | NR1H3 | ACACA, FDPS |
| NR1H4 | 4.26E-07 | AKT1, ESR1, ESR2, IGF1R, INSR, IRS1, KRAS, NOS2, NR0B2, NR1H4, PI3K (complex), PIK3R1, RB1, TP53 | ACACA, ACTC1, ACTN4, ECH1, FDPS, HNRNPD, IDH3A, ITIH4, MAPK14, PAFAH1B2, PGD, PKM, PLEC, SERPINH1, SNX5, SORBS1, TGM2, TOP2B, Tpm1 |
| NR1I2 | 4.86E-04 | CD36, NR1I2, P38 MAPK | ACACA, GSTM1, ICAM1, PGD, TGM2 |
| NR1I2 | 1.83E-03 | NR1I2 | GSTM1, PGD, TGM2 |
| ODC1 | 8.37E-07 | BID, Ck2, ERK, Mapk, MKNK1, ODC1, PGR, PTEN, RB1 | ACACA, ACTC1, CTSB, EEF1D, IDH3A, LDHB, NQO2, PGD, SERPINH1, SUCLG2, TGM2, Tpm1 |
| PDK4 | 4.10E-05 | AMPK, MTORC1, PDK4 | FDPS, IDH3A, LDHB, PKM |
| PDK4 | 1.08E-02 | PDK4 | PKM |
| PLIN1 | 4.45E-03 | LIPE, PLIN1 | ACACA, FBP2 |
| PPARA | 8.36E-03 | PPARA | ACACA, ECH1, FDPS, POR |
| PPARD | 4.58E-02 | PPARD | ECH1, LDHB |
| PPARG | 8.36E-03 | PPARG | ACACA, FDPS, ICAM1, SORBS1 |
| PPARGC1A | 4.95E-02 | PPARGC1A | ACACA, IDH3A |
| PRKAA2 | 2.09E-02 | PRKAA2 | ITIH5, TGM2 |
| PTEN | 5.19E-04 | PTEN | ACACA, IDH3A, LDHB, SUCLG2 |
| SCD | 1.18E-05 | AMPK, INSR, NOS2, P38 MAPK, SCD, STAT1 | ACACA, ACTC1, FDPS, ICAM1, IDH3A, ITIH4, LDHB, PSME1, SERPINH1 |
| SIRT1 | 2.87E-07 | AMPK, ERK1/2, estrogen receptor, IRS2, MTORC1, NFE2L2, NFkB (complex), NOS2, PGR, PI3K (complex), PPARG, PRKAA1, RELA, SERPINE1, SIRT1, STAT1, TGFB1, TP53, XBP1 | ACTN4, ECH1, FBLN5, FDPS, GSTM1, IDH3A, ITIH4, ITIH5, LDHB, MSN, NQO2, PAFAH1B2, PKM, PSME1, RPLP0, SERPINH1, SNX5, SORBS1, TGM2, TOP2B, Tpm1, TXNDC5 |
| SIRT1 | 1.57E-05 | AMPK, MTORC1, NFkB (complex), PI3K (complex), PPARG, RELA, SIRT1, TP53, XBP1 | ACTN4, ECH1, FDPS, IDH3A, LDHB, PAFAH1B2, PKM, SERPINH1, SNX5, SORBS1, TGM2, TOP2B, Tpm1, TXNDC5 |
| SMPD1 | 2.35E-02 | SMPD1 | CTSB |
| SREBF1 | 3.80E-03 | SREBF1 | ACACA, FDPS, RPLP0 |
| SREBF2 | 5.66E-03 | SREBF2 | ACACA, FDPS |
| Srebp | 8.61E-03 | Srebp (complex) | PGD |
| STAT5A | 3.94E-07 | CHEK2, estrogen receptor, IGF1R, MAPK9, MTOR, PGR, PIK3R1, STAT5A, TP53, TRIM28 | ACACA, ACTN4, ALAD, CTSB, ECH1, ICAM1, LDHB, MAPK14, MSN, NQO2, PAFAH1B2, PKM, SERPINH1, SNX5, SORBS1, TGM2, TOP2B, Tpm1 |
| STAT5A | 5.57E-06 | estrogen receptor, STAT5A, TP53 | ACTN4, CTSB, ECH1, ICAM1, MSN, PAFAH1B2, SERPINH1, SNX5, SORBS1, TGM2, TOP2B, Tpm1 |
| **obob** |  |  |  |
| **Master Regulator** | **p-value of overlap** | **Participating regulators** | **Target molecules in dataset** |
| ADIPOQ | 1.05E-06 | ADIPOQ, Akt, AMPK, CTNNB1, EIF4E, HIF1A, IGF1R, Jnk, KRAS, MKNK1, MTOR, NFE2L2, NFkB (complex), P38 MAPK, PADI2, PI3K (complex), RB1, RELA, RHOA, STK11, TCF7L2, TP53 | ACACA, ALDH9A1, Ces1b/Ces1c, ERAP1, FAH, FBP1, GBE1, GPC4, GPS1, IDH3A, MAPK14, MDH1, NAE1, NME1, PARK7, PDHB, PRDX4, RAB6B, THOP1 |
| ADIPOQ | 8.41E-03 | ADIPOQ | ACACA, PDHB |
| AGPAT2 | 3.04E-02 | AGPAT2 | ACACA |
| APOB | 4.79E-02 | APOB | ACACA |
| ATM | 5.20E-04 | ATM, IKBKB, JUN, MAPK1, NFkB (complex), RELA, SOD2, TP53 | ALDH9A1, Ces1b/Ces1c, ERAP1, GPT, LAMA4, LMNB1, NME1, TGFBI, THOP1, YWHAZ |
| CEBPB | 2.39E-07 | ACLY, ATM, BAX, CASP1, CDK2, CEBPB, EIF4E, HIF1A, IGF1R, IKBKB, INSR, JUN, KRAS, NFkB (complex), PI3K (complex), PRKCE, SMARCA4, SOD2, TP53 | ACACA, ALDH9A1, Ces1b/Ces1c, CTSS, CYCS, ERAP1, FAH, FBP1, GPS1, GPT, IDH3A, LMNB1, MAPK14, NAE1, NME1, PDHB, PRDX4, TGFBI, THOP1, YWHAZ |
| CEBPB | 3.54E-04 | ACLY, CASP1, CEBPB, INSR, NFkB (complex), TP53 | ACACA, ALDH9A1, Ces1b/Ces1c, CYCS, ERAP1, IDH3A, NME1, PDHB, TGFBI, THOP1 |
| CEBPD | 2.96E-04 | BAX, BID, CASP3, CEBPD, HIF1A, JUN, RB1, TP53 | ALDH9A1, Ces1b/Ces1c, CTSS, GPT, MDH1, NAE1, NME1, PARK7, TGFBI, THOP1 |
| CYP7A1 | 2.51E-02 | ACACA |  |
| DGAT1 | 3.43E-06 | Akt, BAX, DGAT1, HIF1A, IKBKB, INSR, IRS1, JUN, KRAS, MAPK8, MTOR, PADI2, Pkc(s), PRKCB, RB1 | ACACA, CTSS, CYCS, FAH, GPT, IDH3A, LMNB1, MDH1, NAE1, NME1, PARK7, PDHB, PRDX4, RAB6B |
| ELOVL5 | 2.51E-02 | ELOVL5 | ACACA |
| ERN1 | 9.50E-03 | ERN1 | ACACA, GPT |
| Esrra | 2.47E-03 | AMPK, Esrra, RELA | ERAP1, FBP1, IDH3A, PDHB |
| Esrra | 8.41E-03 | Esrra | FBP1, IDH3A |
| estrogen receptor | 4.66E-05 | BID, EIF4E, ERK, estrogen receptor, Hsp27, IKBKB, IRS1, KRAS, Mapk, MAPK1, MKNK1, NOS2, PI3K (complex), RB1, TP53 | ACACA, ALDH9A1, Ces1b/Ces1c, CTSS, CYCS, FAH, GPC4, GPS1, LAMA4, LMNB1, NME1, PARK7, PRDX4, TGFBI, THOP1 |
| FBXW7 | 1.90E-04 | AMPK, Cyclin E, ESR1, Esrra, FBXW7, MAPK1, MYC, NCOA3, NOTCH1, PPARGC1A, RB1, SMARCA4, TP53 | ACACA, ALDH9A1, Ces1b/Ces1c, CTSS, CYCS, FBP1, GPT, IDH3A, LAMA4, NME1, PARK7, PDHB, TGFBI, THOP1 |
| FTO | 1.59E-04 | EIF4E, FTO, GSK3B, HIF1A, IGF1R, IKBKB, INSR, JUN, KRAS, Map3k7, MAPK1, MKNK1, MTOR, MYC, NFE2L2, P38 MAPK, PADI2, Pkc(s), PLCB3, PRKCA, PRKCD, PRKCE, PRKCQ | FAH, GBE1, GPC4, GPS1, GPT, IDH3A, LAMA4, LMNB1, MAPK14, MDH1, NAE1, PDHB, PRDX4, RAB6B, TGFBI |
| GH1 | 1.35E-04 | CD38, EIF4E, ERBB2, ERK, ERN1, GH1, HIF1A, IGF1R, JAK1, JUN, KRAS, MAPK1, MAPK3, MKNK1, MYC, NFE2L2, PI3K (complex), Pka, PLCB1, RARA, RB1, RHO, Rock, RXRA | ACACA, AK3, CTSS, CYCS, ERAP1, FAH, GBE1, GPC4, GPS1, GPT, LAMA4, MAPK14, NAE1, PARK7, TGFBI |
| GH1 | 1.65E-03 | GH1 | ACACA, GPT, YWHAZ |
| IL1B | 4.51E-05 | EIF4E, ERK, GSK3B, HIF1A, IGF1R, IKBKB, IL1B, INSR, IRS1, JAK1, JUN, KRAS, Mapk, MAPK1, MAPK12, MAPK8, MKNK1, MMP9, NFkB (complex), NOS2, OGG1, P38 MAPK, PADI2, SOD2 | ACACA, CTSS, CYCS, FAH, GPC4, GPS1, GPT, IDH3A, LAMA4, LMNB1, MAPK14, NAE1, PDHB, PRDX4, RAB6B, TGFBI, YWHAZ |
| IL1B | 2.42E-04 | IGF1R, IKBKB, IL1B, INSR, IRS1, JUN, MAPK1, NOS2, SOD2 | ACACA, CTSS, CYCS, GPT, IDH3A, LAMA4, LMNB1, MAPK14, PDHB, TGFBI, YWHAZ |
| Ins1 | 7.54E-07 | AKT1, AMPK, BID, EIF4E, IKBKB, Ins1, KRAS, Mapk, MKNK1, NOS2, PI3K (complex), RB1, TP53 | ALDH9A1, Ces1b/Ces1c, CTSS, CYCS, FAH, GPC4, GPS1, IDH3A, LMNB1, MDH1, NME1, PARK7, PDHB, PRDX4, TGFBI, THOP1 |
| INSIG2 | 2.69E-02 | INSIG2 | ACACA |
| INSR | 3.92E-03 | INSR | CYCS, IDH3A, PDHB |
| IRS1 | 1.19E-03 | IRS1 | ACACA, LAMA4, TGFBI |
| Irs4 | 3.93E-05 | AKT1, AMPK, ERK1/2, IKBKB, IRS1, IRS2, Irs4, KRAS, NOS2, NR3C2, PI3K (complex), RB1, TP53 | ACACA, ALDH9A1, Ces1b/Ces1c, CYCS, FAH, IDH3A, LMNB1, MDH1, NME1, PARK7, PDHB, PRDX4, TGFBI, THOP1 |
| LEPR | 6.61E-06 | AMPK, BAX, CEBPA, EIF4E, ERN1, IKBKB, JUN, KRAS, LEPR, MKNK1, MTOR, NCF1, NOS2, P38 MAPK, PADI2, PI3K (complex), RB1 | ACACA, CTSS, CYCS, FAH, GPC4, GPS1, GPT, IDH3A, LMNB1, MDH1, NME1, PARK7, PDHB, PRDX4, RAB6B |
| LPAR1 | 8.21E-05 | AKT1, BAX, EIF4E, ERK, HIF1A, INSR, JUN, LPAR1, MAPK1, MAPK8, MTOR, N-cor, NFkB (complex), NOS2, PLC, RAC1, RELA, RXRA, TP53 | ACACA, ALDH9A1, Ces1b/Ces1c, CTSS, CYCS, ERAP1, GPS1, GPT, LAMA4, MDH1, NAE1, NME1, TGFBI, THOP1 |
| LPIN1 | 1.29E-04 | BAX, CEBPA, EIF4E, ERK1/2, HIF1A, IKBKB, INSR, Jnk, JUN, LPIN1, MKNK1, MTOR, NFE2L2, NR3C2, P38 MAPK, PPARG, PRKCE, RB1 | ACACA, CTSS, CYCS, GBE1, GPC4, GPS1, GPT, IDH3A, LMNB1, MDH1, NAE1, NME1, PARK7, PDHB |
| Mup1 (includes others) | 1.62E-02 | Mup1 (includes others) | CYCS |
| NR1H3 | 1.48E-04 | AKT1, AMPK, ERK1/2, GSK3B, HIF1A, IGF1R, MAPK1, MTOR, MYC, NR1H3, NR3C2, TP53 | ACACA, ALDH9A1, Ces1b/Ces1c, CYCS, FBP1, GPT, IDH3A, LAMA4, MAPK14, MDH1, NAE1, NME1, PDHB, THOP1 |
| NR1H3 | 7.78E-03 | NR1H3 | ACACA, FBP1 |
| NR1H4 | 3.33E-06 | AKT1, ESR1, IGF1R, IKBKB, INSR, KRAS, NR0B2, NR1H4, PI3K (complex), RB1, TP53 | ACACA, ALDH9A1, Ces1b/Ces1c, CTSS, CYCS, FAH, IDH3A, LMNB1, MAPK14, MDH1, NME1, PARK7, PDHB, PRDX4, TGFBI, THOP1 |
| ODC1 | 5.26E-05 | BID, Ck2, EIF4E, Mapk, MKNK1, ODC1, PTEN, RB1, ZFP36 | ACACA, CTSS, GPC4, GPS1, IDH3A, MDH1, NME1, PARK7, PDHB |
| PLIN1 | 1.80E-02 | PLIN1 | ACACA |
| PPARA | 2.95E-02 | PPARA | ACACA, GPT, HPX |
| PPARD | 3.33E-02 | PPARD | ALDH9A1, FBP1 |
| PPARG | 4.49E-03 | PPARG | ACACA, GPT, MDH1, PDHB |
| PPARGC1A | 2.69E-04 | AMPK, Esrra, PPARGC1A | ACACA, CYCS, FBP1, IDH3A, PDHB |
| PPARGC1A | 2.54E-03 | PPARGC1A | ACACA, CYCS, IDH3A |
| PPARGC1B | 1.44E-03 | PPARGC1B | ACACA, CYCS |
| PTEN | 2.64E-04 | PTEN | ACACA, IDH3A, MDH1, PDHB |
| RBL1 | 6.49E-04 | Akt, CDK2, EIF4E, ERK, ESR1, FOXO3, HIF1A, IKBKB, MTOR, NOS2, RAF1, RB1, RBL1, RXRA, SMARCA4, SREBF2 | ACACA, CTSS, CYCS, FBP1, GPS1, GPT, LMNB1, MDH1, NAE1, NME1, PARK7 |
| SIT1 | 1.59E-04 | BAX, CEBPA, EIF4E, ERK1/2, HIF1A, IKBKB, INSR, IRS1, JUN, MAPK8, MKNK1, MTOR, NFkB (complex), NR3C2, P38 MAPK, PADI2, PPARG, RELA, RFWD2, SIT1 | ACACA, CTSS, CYCS, ERAP1, GPC4, GPS1, GPT, IDH3A, LMNB1, MDH1, NAE1, PDHB, RAB6B |
