# Supplementary material for: Adipokinome Signatures in Obese Mouse Models Reflect Adipose Tissue Health and Are Associated with Serum Lipid Composition
Source: Int J Mol Sci. 2019 May 24;20(10):2559. doi: 10.3390/ijms20102559 (PMC6567124; doi:10.3390/ijms20102559)
Supplement: Supplementary file 1 [file ijms-20-02559-s001.zip › IJMS Table S6B.docx]

**Supplement Table 6B:** **Abundance of fibrosis related proteins in adipokines correlated to liver-to-adipose-tissue DNL-ratio.**

Proteins with correlation to DNL-ratio were subjected to IPA^®^ Core analyses. Upstream regulating molecules or master regulators of signaling networks involved in fibrosis were screened for genes involved in fibrosis (Ingenuity®, Qiagen).

| **C57Bl6** |  |  |  |
| --- | --- | --- | --- |
| **Master Regulator/ Upstream Regulator** | **p-value of overlap** | **Participating regulators** | **Target molecules in dataset** |
| ADIPOQ | 4.48E-07 | ADIPOQ, AMPK, CREB1, CTNNB1, estrogen receptor, IRS1, Jnk, MKNK1, MTOR, NFE2L2, NFkB (complex), NLRP3, P38 MAPK, PGR, PPARG, RELA, RHOA, TCF7L2, TP53 | ABAT, AKR1A1, ATL3, CYP2E1, DPYSL3, EPB41L2, GSR, GSTM1, GSTM3, KRT8, LAP3, LDHB, Otub1, PDXK, PICALM, PLA2G7, PLIN1, PSMD2, RPL12, SERPIND1, THOP1, UBE2N, YWHAH |
| ADORA2A | 3.16E-02 | ADORA2A | ATP6V1B2, GDI1 |
| ADORA2A | 7.51E-07 | ADORA2A, ATF6, CAV1, Creb, CREB1, estrogen receptor, Mapk, MAPK1, MAPK3, MKNK1, NFE2L2, NFkB (complex), NLRP3, NTRK1, P38 MAPK, PGR, Pka, PPARG, RHO, TP53, TP63 | ABAT, ATL3, ATP6V1B2, CRABP1, DPYSL3, EPB41L2, GDI1, GSTM1, GSTM3, KRT8, LAP3, PDIA4, PDXK, PLA2G7, PLIN1, PSMD2, PTRF, SERPIND1, THOP1, UBE2N, VIL1, YWHAH |
| ADORA2B | 4.23E-06 | ADORA2B, ATF6, BAK1, BAX, CREB1, estrogen receptor, MKNK1, NFkB (complex), NLRP3, P38 MAPK, PGR, PPARG, PTEN, TP53, TP63 | ABAT, ATL3, DLAT, DPYSL3, EPB41L2, GSR, GSTM3, KRT8, LDHB, PDIA4, PDIA6, PDXK, PLA2G7, PLIN1, PSMD2, THOP1, UBE2N, VIL1, YWHAH |
| AGT | 4.05E-05 | AGT, AGTR1, BAX, CREB1, ERK1/2, estrogen receptor, IKBKB, MAPK8, NFkB (complex), NLRP3, P38 MAPK, REL, SYK, TP53, XDH | ABAT, ACAD9, ATL3, CALR, COTL1, CYP2E1, GM2A, GSR, KRT8, PDIA6, PDXK, PLA2G7, PSMD2, THOP1, UBE2N, YWHAH |
| AGTR1 | 1.62E-05 | AGTR1, Akt, ERK1/2, estrogen receptor, IKBKB, Jnk, Mapk, MKNK1, NFE2L2, NFkB (complex), NLRP3, P38 MAPK, Pkc(s), PLCB1, RB1, RELA, TP53, TSC2 | ABAT, AKR1A1, ATL3, ATP6V1B2, CALR, DLAT, DPYSL3, EPB41L2, GM2A, GSTM1, KRT8, LDHB, PDIA4, PLA2G7, PSMD2, SERPIND1, THOP1, UBE2N, YWHAH |
| Agtr1b | 1.24E-05 | Agtr1b, Mapk, MAPK1, MAPK14, MAPK3, MKNK1, NFE2L2, RB1, RELA, TP53 | ABAT, AKR1A1, ATL3, CALR, DLAT, DPYSL3, EPB41L2, GSR, GSTM1, KRT8, LAP3, PDIA4, PDIA6, PSMD2, SERPIND1, THOP1, YWHAH |
| AKT1 | 2.52E-08 | AKT1, AR, ATF6, BCL10, BRCA1, EGFR, ERK1/2, FOXO1, HMOX1, INPP5D, MAPK1, MKNK1, N-cor, NFkB (complex), NLRP3, P38 MAPK, PGR, REL, TP53, TP63, TP73, TSC2 | ABAT, ACAD9, ATL3, ATP6V1B2, CALR, COTL1, DPYSL3, EPB41L2, GDI1, GSR, GSTM1, GSTM3, KRT8, LAP3, LCP1, PCBP2, PDIA4, PDIA6, PLA2G7, PLIN1, PLS3, PSMD2, THOP1, UBE2N, VIL1, YWHAH |
| ALOX5 | 6.20E-08 | ALOX5, ATF6, EIF2AK3, ERK, ERK1/2, ERN1, IKBKB, MKNK1, MTOR, MYC, NFE2L2, NOS2, P38 MAPK, PGR, PPARG, SYK/ZAP, TP53, TP63 | ABAT, AKR1A1, ATL3, CALR, CYP2E1, DLAT, DPYSL3, EPB41L2, GM2A, GSTM1, GSTM3, KRT8, LDHB, Otub1, PDIA4, PLA2G7, PLIN1, PSMD2, RPL12, SERPIND1, THOP1, VIL1, YWHAH |
| APC | 2.68E-04 | APC, CTNNB1, MTOR | AKR1A1, CYP2E1, LAP3, LDHB, Otub1, RPL12 |
| AR | 4.94E-02 | AR | GDI1, GSTM1, PLS3 |
| ATF3 | 4.17E-06 | Akt, ATF3, ERK, IKBKB, Jnk, MKNK1, MTOR, NFE2L2, NOS2, P38 MAPK, PGR, PPARG, RB1, TP53, TP63, TSC2 | ABAT, ATL3, ATP6V1B2, CYP2E1, DLAT, DPYSL3, EPB41L2, GM2A, GSTM1, GSTM3, KRT8, Otub1, PLA2G7, PLIN1, PSMD2, RPL12, SERPIND1, THOP1, VIL1, YWHAH |
| BARD1 | 2.29E-05 | BARD1, CREB1, ERK1/2, ESR1, ESR2, MAPK14, MTOR, PGR, TP53, TP73 | ABAT, AKR1A1, ATL3, CALR, GM2A, GSR, GSTM3, KRT8, LCP1, LDHB, Otub1, PDIA6, PDXK, PSMD2, RPL12, THOP1, YWHAH |
| BCL6 | 6.92E-07 | AR, BCL6, CDKN2A, ERK1/2, IKBKB, Jnk, MAPK14, MKNK1, NFE2L2, NFkB (complex), NLRP3, P38 MAPK, PGR, PPARG, TP53, TP63, TP73 | ABAT, ATL3, CALR, DLAT, DPYSL3, EPB41L2, GDI1, GM2A, GSR, GSTM1, GSTM3, KRT8, LCP1, PDIA6, PLA2G7, PLIN1, PLS3, PSMD2, SERPIND1, THOP1, UBE2N, VIL1, YWHAH |
| BID | 1.49E-04 | BAK1, BID, CASP8, CHEK1, E2F3, ERK1/2, NLRP3, TP53, TRAF2 | ABAT, ATL3, CALR, DAG1, GSR, KRT8, PDIA6, PSMD2, PYGL, THOP1, UBE2N, YWHAH |
| BUB1B | 4.80E-06 | AR, BUB1B, CDK1, MAPK14, MTOR, PGR, TP53, TP73 | ABAT, AKR1A1, ATL3, CALR, GDI1, GSR, GSTM1, GSTM3, KRT8, LCP1, LDHB, Otub1, PDIA6, PLS3, PSMD2, RPL12, THOP1, YWHAH |
| C5 | 1.12E-06 | Akt, AMPK, ATF6, C5, Creb, ERK, ERK1/2, estrogen receptor, IKBKB, MAP2K1, MKNK1, MMP9, MTOR, NFkB (complex), NLRP3, P38 MAPK, PGR, PPARG, TGFB1, TP53, TP63, TSC2 | ABAT, AKR1A1, ATL3, ATP6V1B2, CALR, DPYSL3, EPB41L2, GM2A, GSR, GSTM3, KRT8, LDHB, Otub1, PDIA4, PLA2G7, PLIN1, PSMD2, RPL12, SELENBP1, THOP1, UBE2N, VIL1, YWHAH |
| CAPNS1 | 3.20E-05 | Akt, CAPNS1, ERK1/2, estrogen receptor, IKBKB, Jnk, NFE2L2, NFkB (complex), NLRP3, P38 MAPK, RB1, TP53, TSC2 | ABAT, AKR1A1, ATL3, ATP6V1B2, CALR, DLAT, GM2A, GSTM1, KRT8, LDHB, PDIA4, PLA2G7, PSMD2, SERPIND1, THOP1, UBE2N, YWHAH |
| CASP1 | 1.32E-09 | ATF6, BAX, CASP1, estrogen receptor, IKBKB, MKNK1, MPO, MTOR, NFkB (complex), NLRP3, P38 MAPK, PGR, PPARG, SERPINE1, TP53, TP63, TRAF2 | ABAT, AKR1A1, ATL3, CALR, DLAT, DPYSL3, EPB41L2, GM2A, GSR, GSTM3, KRT8, LDHB, Otub1, PDIA4, PDIA6, PLA2G7, PLIN1, PSMD2, PYGL, RPL12, THOP1, UBE2N, VIL1, YWHAH |
| CAV1 | 1.86E-02 | CAV1 | KRT8, PTRF |
| CAV1 | 2.62E-05 | AMPK, CAV1, CHUK, ERK1/2, IKBKB, MAPK1, RB1, TP53 | ABAT, ATL3, CALR, DLAT, GM2A, GSR, KRT8, LAP3, LDHB, PDIA6, PSMD2, PTRF, THOP1, YWHAH |
| CBL | 6.73E-07 | AKT1, BAK1, CBL, CREB1, CTNNB1, EGFR, ERK, HGS, IGF1R, MAPK1, MAPK14, MAPK3, MTOR, NFE2L2, NOS2, PGR, PI3K (complex), PRKAA, STAT3, TFEB, TGFB1, TGM2, TP53, TP63, TSC2 | ABAT, AKR1A1, ATL3, ATP6V1B2, CALR, CYP2E1, GSR, GSTM1, GSTM3, KRT8, LAP3, LDHB, NLN, Otub1, PDIA6, PDXK, PSMD2, RPL12, SELENBP1, SERPIND1, THOP1, VIL1, YWHAH |
| CD28 | 1.70E-05 | AKT1, AR, CAV1, CD28, CDK1, FMR1, INPP5D, Jnk, JUNB, KDM5A, KDM5B, MAPK1, MAPK14, MKNK1, MTOR, N-cor, NFE2L2, P38 MAPK, PI3K (complex), PPARG, PRKACA, RB1, REL, RICTOR, RPS6KB1, SP1, STAT3, TCR, TFEB, TGM2, TP63, TSC2 | ACAD9, AKR1A1, ATP6V1B2, COTL1, DAG1, DLAT, DPYSL3, EPB41L2, GDI1, LAP3, LDHB, NLN, Otub1, PDIA4, PLA2G7, PLIN1, PLS3, PTRF, RPL12, SERPIND1, VIL1 |
| CD36 | 7.22E-07 | Akt, ATF6, BAK1, BAX, CAV1, CD36, CFTR, ERK1/2, ERN1, MKNK1, MMP9, MTOR, P38 MAPK, PGR, Pka, PPARG, RB1, RHO, TGFB1, TP63, TSC2 | AKR1A1, ATP6V1B2, CALR, CRABP1, CYP2E1, DLAT, DPYSL3, EPB41L2, GSR, GSTM3, Otub1, PDIA4, PDIA6, PLA2G7, PLIN1, PTRF, RPL12, SELENBP1, VIL1 |
| CD44 | 3.47E-06 | CASP8, CAV1, CD44, CREB1, CTNNB1, EGFR, ESR1, MAPK3, MKNK1, NFE2L2, NLRP3, P38 MAPK, PPARG, PRKCE, REL, SRC, SYK, TCF7L2, TGFB1, TGM2, TP53, TP63, TRAF2 | ABAT, ACAD9, ATL3, COTL1, CYP2E1, DPYSL3, EPB41L2, GSR, GSTM1, NLN, PDXK, PICALM, PLA2G7, PLIN1, PSMD2, PTRF, PYGL, SELENBP1, SERPIND1, THOP1, UBE2N, VIL1, YWHAH |
| CD74 | 3.99E-05 | Akt, ATF6, CD74, IKBKB, MKNK1, MTOR, NFkB (complex), NLRP3, P38 MAPK, PGR, PPARG, REL, SYK, TP63, TSC2 | ACAD9, AKR1A1, ATP6V1B2, COTL1, DPYSL3, EPB41L2, GM2A, GSTM3, LDHB, Otub1, PDIA4, PLA2G7, PLIN1, RPL12, UBE2N, VIL1 |
| CDH1 | 3.26E-08 | CAV1, CDH1, CREB1, CTNNB1, EGFR, ERK, estrogen receptor, IKBKB, Mapk, MKNK1, MTOR, MYC, NFkB (complex), NLRP3, P38 MAPK, PGR, SRC, TCF7L2, TP53 | ABAT, AKR1A1, ATL3, CYP2E1, DPYSL3, EPB41L2, GM2A, GSR, GSTM3, KRT8, LAP3, LDHB, Otub1, PDIA6, PDXK, PICALM, PLA2G7, PSMD2, PTRF, RPL12, THOP1, UBE2N, YWHAH |
| CREB1 | 3.95E-04 | CREB1, P38 MAPK, RHOA, TP53 | ABAT, ATL3, GSR, KRT8, PDIA6, PDXK, PLA2G7, PSMD2, THOP1, YWHAH |
| CXCL12 | 2.98E-07 | Akt, ATF6, CHUK, CXCL12, ERK, ERK1/2, MAPK1, Mek, MKNK1, MTOR, NFKBIA, P38 MAPK, PGR, PI3K (family), PPARG, PTK2, REL, RHOA, ROCK1, SYK, TCF7L2, TP53, TP63, TSC2 | ABAT, ACAD9, AKR1A1, ATL3, ATP6V1B2, CALR, COTL1, DPYSL3, EPB41L2, GM2A, GSR, GSTM3, KRT8, LAP3, LDHB, Otub1, PDIA4, PICALM, PLA2G7, PLIN1, PSMD2, RPL12, THOP1, VIL1, YWHAH |
| CYP2E1 | 1.82E-08 | ATF6, BAK1, BAX, CYP2E1, ERK1/2, estrogen receptor, IKBKB, MKNK1, MTOR, NFkB (complex), NLRP3, P38 MAPK, PGR, PPARG, TP53, TP63 | ABAT, AKR1A1, ATL3, CALR, DLAT, DPYSL3, EPB41L2, GM2A, GSR, GSTM3, KRT8, LDHB, Otub1, PDIA4, PDIA6, PLA2G7, PLIN1, PSMD2, RPL12, THOP1, UBE2N, VIL1, YWHAH |
| DCN | 8.53E-06 | Akt, BAX, CASP3, CAV1, CTNNB1, DCN, ERBB2, ERBB3, IGF1R, IKBKB, JUNB, Mapk, MKNK1, MTOR, P38 MAPK, PDPK1, PGR, RICTOR, TP53 | ABAT, AKR1A1, ATL3, CYP2E1, DPYSL3, EPB41L2, GM2A, GSR, GSTM3, KRT8, LDHB, Otub1, PDIA6, PLA2G7, PLIN1, PSMD2, PTRF, RPL12, THOP1, YWHAH |
| DES | 2.49E-06 | Akt, CHUK, DES, ERK1/2, estrogen receptor, MTOR, NFkB (complex), NFKBIA, NLRP3, P38 MAPK, RB1, TP53, TSC2 | ABAT, AKR1A1, ATL3, ATP6V1B2, CALR, DLAT, GM2A, GSR, KRT8, LDHB, Otub1, PDIA6, PLA2G7, PSMD2, RPL12, THOP1, UBE2N, YWHAH |
| ELANE | 9.48E-06 | CHUK, CYP1A1, ELANE, ERK1/2, estrogen receptor, IKBKB, IL1B, MAPK1, MAPK3, NFE2L2, NFkB (complex), NLRP3, P38 MAPK, RELA, TP53 | ABAT, AKR1A1, ATL3, CALR, CYP2E1, GM2A, GSR, GSTM1, GSTM3, KRT8, LAP3, LCP1, PDIA4, PDIA6, PLA2G7, PSMD2, SERPIND1, THOP1, UBE2N, YWHAH |
| Esrra | 6.13E-05 | AMPK, ERK1/2, ERN1, Esrra, MAPK1, P38 MAPK, PRKAA1, PRKAA2, RELA, TP53 | ABAT, ATL3, CALR, CYP2E1, DLAT, GSR, KRT8, LAP3, LDHB, PDIA6, PLA2G7, PSMD2, PYGL, THOP1, YWHAH |
| Esrra | 1.41E-02 | Esrra | DLAT, PYGL |
| FASLG | 1.00E-07 | ATF6, BAK1, BAX, CASP3, CASP8, CAV1, ERK1/2, FASLG, FYN, IKBKB, MAPK1, MAPK8, MKNK1, MTOR, NFkB (complex), NLRP3, NOS2, P38 MAPK, PGR, PPARG, PTK2B, SRC, TP53, TP63 | ABAT, AKR1A1, ATL3, CALR, CYP2E1, DPYSL3, EPB41L2, GM2A, GSR, GSTM3, LAP3, LDHB, Otub1, PDIA4, PDIA6, PLA2G7, PLIN1, PSMD2, PTRF, RPL12, THOP1, UBE2N, VIL1, YWHAH |
| FCGR2A | 1.42E-07 | Akt, BAX, CASP1, CREB1, ERK, FCGR2A, IKBKB, INPP5D, MKNK1, MTOR, NFkB (complex), NLRP3, Pkc(s), PPARA, RB1, REL, SYK, TP53, TSC2 | ABAT, ACAD9, AKR1A1, ATL3, ATP6V1B2, COTL1, DLAT, DPYSL3, EPB41L2, GM2A, GSR, GSTM3, KRT8, Otub1, PDIA6, PDXK, PLIN1, PSMD2, RPL12, SELENBP1, THOP1, UBE2N, YWHAH |
| FGFR2 | 4.02E-06 | CHUK, CREB1, CTNNB1, EGFR, ERBB2, ERBB3, estrogen receptor, FGFR2, IKBKB, Mapk, MKNK1, MTOR, NFkB (complex), NLRP3, P38 MAPK, PGR, Ras, RB1, RICTOR, TP53, TSC2 | ABAT, AKR1A1, ATL3, CYP2E1, DLAT, DPYSL3, EPB41L2, GM2A, GSR, GSTM3, KRT8, LDHB, Otub1, PDIA6, PDXK, PLA2G7, RPL12, THOP1, UBE2N, YWHAH |
| FN1 | 3.89E-07 | Akt, BAX, calpain, CAV1, CREB1, CTNNB1, ERK1/2, estrogen receptor, FN1, IGF1R, IKBKB, ILK, IRS1, ITGAV, ITGB3, JUNB, Mapk, MKNK1, MTOR, MYC, NFkB (complex), NLRP3, PTK2, RB1, SRC, TCF7L2, TGFB1, TP53, TSC2 | ABAT, AKR1A1, ATL3, ATP6V1B2, CALR, CYP2E1, DLAT, DPYSL3, EPB41L2, GM2A, GSR, KRT8, LDHB, Otub1, PDIA6, PDXK, PICALM, PLIN1, PSMD2, PTRF, RPL12, SELENBP1, THOP1, UBE2N, YWHAH |
| HCK | 7.56E-10 | Akt, ATF6, BAK1, BAX, BTK, CHUK, ERK, estrogen receptor, HCK, IKBKB, IKBKG, INPP5D, MAPK1, MAPK3, MAPK8, MKNK1, MTOR, NFE2L2, NFkB (complex), NLRP3, NOS2, P38 MAPK, PGR, PI3K (complex), PPARG, REL, SYK, TP53, TP63, TP73, TSC2 | ABAT, ACAD9, AKR1A1, ATL3, ATP6V1B2, COTL1, CYP2E1, DPYSL3, EPB41L2, GM2A, GSR, GSTM1, GSTM3, KRT8, LAP3, LCP1, Otub1, PDIA4, PDIA6, PLA2G7, PLIN1, PSMD2, RPL12, SERPIND1, THOP1, UBE2N, VIL1, YWHAH |
| HMGB1 | 5.26E-07 | ATF6, CAV1, HIF1A, HMGB1, IKBKB, MAPK14, MKNK1, MTOR, P38 MAPK, PGR, PPARG, TP53, TP63 | ABAT, AKR1A1, ATL3, CALR, DPYSL3, EPB41L2, GM2A, GSR, GSTM3, KRT8, LDHB, Otub1, PDIA4, PLA2G7, PLIN1, PSMD2, PTRF, RPL12, THOP1, VIL1, YWHAH |
| HNF4A | 3.74E-02 | HNF4A | CYP2E1, KRT8, PYGL |
| Hsp70 | 8.49E-06 | Akt, BAX, CDK5, ERK1/2, estrogen receptor, Hsp70, IKBKB, MTOR, NFkB (complex), NLRP3, P38 MAPK, RB1, TP53, TRAF2, TSC2 | ABAT, AKR1A1, ATL3, ATP6V1B2, CALR, DLAT, GM2A, GSR, KRT8, Otub1, PDIA6, PLA2G7, PSMD2, PYGL, RPL12, THOP1, UBE2N, YWHAH |
| HSPB1 | 8.48E-07 | Akt, BAX, CASP3, CAV1, CREB1, CTNNB1, HSPB1, IKBKB, Jnk, MAP3K5, MAPK1, MAPK14, MTOR, NFE2L2, PRKCD, PRKD1, PTEN, RB1, TP53, TRAF2, TSC2 | ABAT, ATL3, ATP6V1B2, CYP2E1, DLAT, GM2A, GSR, GSTM1, KRT8, LAP3, LDHB, Otub1, PDIA6, PDXK, PSMD2, PTRF, PYGL, RPL12, SERPIND1, THOP1, YWHAH |
| HSPB6 | 3.55E-08 | ATF6, BAK1, BAX, HSPB6, IKBKB, MAP3K5, MKNK1, MTOR, P38 MAPK, PGR, PPARG, TP53, TP63 | ABAT, AKR1A1, ATL3, DLAT, DPYSL3, EPB41L2, GM2A, GSR, GSTM3, KRT8, LDHB, Otub1, PDIA4, PDIA6, PLA2G7, PLIN1, PSMD2, RPL12, THOP1, VIL1, YWHAH |
| IGF1R | 9.15E-08 | AR, ATM, CASP8, CAV1, CDK1, CHEK1, CHEK2, E2F3, ERK1/2, IGF1R, JAK1, Jnk, JUNB, MKNK1, MYC, NFE2L2, NLRP3, NR3C1, PGR, RB1, STAT3, TP53, TP63, TP73, TRIM28 | ABAT, ALAD, ATL3, CALR, DAG1, DLAT, DPYSL3, EPB41L2, GDI1, GSR, GSTM1, GSTM3, KRT8, LCP1, LDHB, PDIA4, PDIA6, PLIN1, PLS3, PSMD2, PTRF, SERPIND1, THOP1, UBE2N, VIL1, YWHAH |
| IGHM | 1.94E-06 | AKT1, BCR (complex), CHUK, CREB1, IGHM, IKBKB, INPP5D, MKNK1, N-cor, NOS2, PI3K (complex), RB1, REL, SYK, TP53, TSC2 | ABAT, ACAD9, ATL3, ATP6V1B2, COTL1, CYP2E1, DLAT, DPYSL3, EPB41L2, GM2A, GSR, KRT8, PDIA6, PDXK, PLIN1, PSMD2, THOP1, YWHAH |
| IKBKB | 1.16E-11 | ATF6, BCL10, CREB1, CTNNB1, IKBKB, IRS1, Jnk, MKNK1, MMP9, MTOR, NFE2L2, NFkB (complex), NLRP3, P38 MAPK, PPARG, REL, TCF7L2, TFEB, TGFB1, TP53, TP73 | ABAT, ACAD9, AKR1A1, ATL3, ATP6V1B2, COTL1, CYP2E1, DLAT, DPYSL3, EPB41L2, GM2A, GSR, GSTM1, KRT8, LAP3, LCP1, LDHB, Otub1, PDIA4, PDIA6, PDXK, PICALM, PLA2G7, PLIN1, PSMD2, RPL12, SELENBP1, SERPIND1, THOP1, UBE2N, YWHAH |
| IKBKB | 4.24E-07 | CTNNB1, IKBKB, MTOR, P38 MAPK, TP53, TP73 | ABAT, AKR1A1, ATL3, CYP2E1, GM2A, GSR, KRT8, LAP3, LCP1, LDHB, Otub1, PDIA6, PLA2G7, PSMD2, RPL12, THOP1, YWHAH |
| IKBKE | 5.85E-07 | Akt, AR, CHUK, CTNNB1, ERK1/2, FOXO1, GSK3B, IKBKE, MTOR, P38 MAPK, PGR, RB1, TFEB, TP53, TP73, TSC2 | ABAT, AKR1A1, ATL3, ATP6V1B2, CALR, CYP2E1, DLAT, GDI1, GM2A, GSR, GSTM1, GSTM3, KRT8, LCP1, LDHB, Otub1, PDIA6, PLA2G7, PLS3, PSMD2, RPL12, THOP1, YWHAH |
| IKBKE | 3.48E-05 | ERK1/2, IKBKE, MTOR, TP53 | ABAT, AKR1A1, ATL3, CALR, GSR, KRT8, LDHB, Otub1, PDIA6, PSMD2, RPL12, THOP1, YWHAH |
| IL12 (complex) | 5.43E-07 | ERK1/2, estrogen receptor, IKBKB, IL12 (complex), MAPK1, MAPK3, MMP9, MTOR, NFE2L2, NFkB (complex), NLRP3, P38 MAPK, PLAU, SOCS1, TGFB1, TP53 | ABAT, ATL3, CALR, GM2A, GSR, GSTM1, KRT8, LAP3, LDHB, Otub1, PDIA4, PDIA6, PLA2G7, PSMD2, RPL12, SELENBP1, SERPIND1, THOP1, UBE2N, YWHAH |
| IL13 | 9.13E-07 | AKT1, ATF6, CAV1, CREB1, ERK1/2, estrogen receptor, HCK, IL13, JAK1, MKNK1, MMP9, NFE2L2, NFkB (complex), P38 MAPK, PGR, PPARG, PRKCD, RB1, SRC (family), STAT3, TGFB1, TP53, TP63, TP73, TRAF2, TSC2 | ABAT, ATL3, ATP6V1B2, CALR, DLAT, DPYSL3, EPB41L2, GSTM1, GSTM3, KRT8, LCP1, PDIA4, PDXK, PLA2G7, PSMD2, PTRF, PYGL, SELENBP1, SERPIND1, THOP1, VIL1, YWHAH |
| IL17A | 2.77E-02 | IL17A | COTL1, SERPIND1 |
| IL17RA | 1.05E-07 | ATF6, CEBPB, IKBKB, IL17RA, MKNK1, MTOR, P38 MAPK, PGR, PPARG, TP53, TP63 | ABAT, AKR1A1, ATL3, DLAT, DPYSL3, EPB41L2, GM2A, GSR, GSTM3, KRT8, LDHB, Otub1, PDIA4, PLA2G7, PLIN1, PSMD2, RPL12, THOP1, VIL1, YWHAH |
| IL-1R | 2.48E-07 | ATF6, BAK1, BAX, CHUK, CTNNB1, ERK1/2, estrogen receptor, GSK3B, IKBKB, IL-1R, MAPK1, MAPK3, MAPK8, MKNK1, NFE2L2, NFkB (complex), NLRP3, P38 MAPK, PGR, PPARG, TFEB, TP53, TP63 | ABAT, ATL3, ATP6V1B2, CALR, CYP2E1, DLAT, DPYSL3, EPB41L2, GM2A, GSR, GSTM1, GSTM3, KRT8, LAP3, PDIA4, PDIA6, PLA2G7, PLIN1, PSMD2, SERPIND1, THOP1, UBE2N, VIL1, YWHAH |
| IL1R1 | 1.19E-10 | ATF6, BAK1, BAX, CAV1, estrogen receptor, IKBKB, IL1R1, MAPK1, MAPK14, MAPK3, MAPK8, MKNK1, MTOR, NFE2L2, NFkB (complex), NLRP3, P38 MAPK, PGR, PPARG, REL, RELA, TP53, TP63, TRAF3 | ABAT, ACAD9, AKR1A1, ATL3, CALR, COTL1, DPYSL3, EPB41L2, GM2A, GSR, GSTM1, GSTM3, KRT8, LAP3, Otub1, PDIA4, PDIA6, PLA2G7, PLIN1, PSMD2, PTRF, PYGL, RPL12, SERPIND1, THOP1, UBE2N, VIL1, YWHAH |
| IL1RN | 6.29E-06 | BAX, CASP3, CASP7, CREB1, CYP1A1, IFNG, IKBKB, IL1RN, MAPK1, NFkB (complex), NLRP3, P38 MAPK, PKC alpha/beta, Pkc(s), PPARA, PRKD1, PTEN, SMPD1, TP53, TP73 | ABAT, ATL3, CYP2E1, DLAT, GM2A, GSR, GSTM3, KRT8, LAP3, LCP1, LDHB, PDIA6, PDXK, PLA2G7, PLIN1, PSMD2, SELENBP1, THOP1, UBE2N, YWHAH |
| IL25 | 1.28E-06 | ERK1/2, estrogen receptor, IKBKB, IL25, Jnk, MKNK1, MTOR, NFE2L2, NFkB (complex), NLRP3, P38 MAPK, PGR, PPARG, TP53, TP63 | ABAT, ATL3, CALR, DLAT, DPYSL3, EPB41L2, GM2A, GSTM1, GSTM3, KRT8, LDHB, Otub1, PLA2G7, PLIN1, PSMD2, RPL12, SERPIND1, THOP1, UBE2N, VIL1, YWHAH |
| INHBA | 5.81E-07 | Akt, ATF6, CTNNB1, ERK1/2, GSK3B, INHBA, MKNK1, MTOR, MYC, P38 MAPK, PGR, PPARG, RB1, TP53, TP63, TSC2 | ABAT, AKR1A1, ATL3, CALR, CYP2E1, DLAT, DPYSL3, EPB41L2, GSR, GSTM3, KRT8, LAP3, LDHB, Otub1, PDIA4, PLA2G7, PLIN1, PSMD2, RPL12, THOP1, VIL1, YWHAH |
| Ins1 | 4.01E-05 | AKT1, CHUK, IKBKB, Ins1, Mapk, MKNK1, N-cor, NOS2, P38 MAPK, PI3K (complex), RB1, TP53, TSC2 | ABAT, ATL3, ATP6V1B2, CYP2E1, DLAT, DPYSL3, EPB41L2, GM2A, GSR, KRT8, PDIA6, PLA2G7, PLIN1, PSMD2, THOP1, YWHAH |
| Integrin | 3.46E-08 | ATF6, CAV1, CHUK, Creb, CTNNB1, ERK1/2, FYN, IKBKB, Integrin, MKNK1, MTOR, MYC, P38 MAPK, PGR, PI3K (complex), Pkc(s), PPARG, PTK2, RHOA, SRC, TCF7L2, TP53, TP63, TSC2 | ABAT, AKR1A1, ATL3, ATP6V1B2, CALR, CYP2E1, DPYSL3, EPB41L2, GM2A, GSR, GSTM3, KRT8, LAP3, LDHB, Otub1, PDIA4, PICALM, PLA2G7, PLIN1, PSMD2, PTRF, RPL12, THOP1, VIL1, YWHAH |
| IRAK2 | 1.72E-08 | AMPK, ATF6, BAK1, BAX, estrogen receptor, IKBKB, IRAK2, MAP2K1, MAPK1, MKNK1, MTOR, NFkB (complex), NLRP3, P38 MAPK, PGR, PPARG, TP53, TP63 | ABAT, AKR1A1, ATL3, DLAT, DPYSL3, EPB41L2, GM2A, GSR, GSTM3, KRT8, LAP3, LDHB, Otub1, PDIA4, PDIA6, PLA2G7, PLIN1, PSMD2, RPL12, THOP1, UBE2N, VIL1, YWHAH |
| ITGAV | 2.79E-08 | Akt, AR, CDK1, CTNNB1, ERK1/2, ITGAV, MTOR, PGR, PPARG, RB1, REL, SYK, TGFB1, TGM2, TP53, TP73, TSC2, VAV3 | ABAT, ACAD9, AKR1A1, ATL3, ATP6V1B2, CALR, COTL1, CYP2E1, DLAT, GDI1, GM2A, GSR, GSTM1, GSTM3, KRT8, LCP1, NLN, Otub1, PDIA6, PLIN1, PLS3, PSMD2, RPL12, SELENBP1, THOP1, YWHAH |
| ITGB3 | 1.61E-07 | AR, CAV1, CDK1, CREB1, CTNNB1, ERK1/2, GSK3B, IGF1R, ITGB3, JUNB, KDR, NFkB (complex), NLRP3, PGR, RB1, REL, SRC, SYK, TCF7L2, TFEB, TP53, TP73, VAV3 | ABAT, ACAD9, ATL3, ATP6V1B2, CALR, COTL1, CYP2E1, DLAT, GDI1, GSR, GSTM1, GSTM3, KRT8, LAP3, LCP1, PDIA6, PDXK, PICALM, PLIN1, PLS3, PSMD2, PTRF, THOP1, UBE2N, YWHAH |
| JAK2 | 1.87E-07 | ATF6, CAV1, CHUK, ERK1/2, HOXA10, IKBKB, JAK2, KDM5B, MAPK1, MKNK1, MTOR, NFKB1, P38 MAPK, PGR, PPARG, RHOA, SP1, SRC (family), TP53, TP63 | ABAT, AKR1A1, ATL3, CALR, CYP2E1, DPYSL3, EPB41L2, GM2A, GSR, GSTM3, LAP3, LDHB, Otub1, PDIA4, PLA2G7, PLIN1, PLS3, PSMD2, PTRF, RPL12, THOP1, VIL1, YWHAH |
| KIT | 3.69E-06 | AKT1, ATF6, ERK1/2, IKBKB, KIT, MAPK1, MKNK1, MTOR, P38 MAPK, PGR, PPARG, TP53, TP63, TSC2 | ABAT, AKR1A1, ATL3, ATP6V1B2, CALR, DLAT, DPYSL3, EPB41L2, GM2A, GSR, GSTM3, KRT8, LAP3, Otub1, PDIA4, PLA2G7, PSMD2, RPL12, THOP1, VIL1, YWHAH |
| Klrk1 | 1.09E-06 | Akt, CTNNB1, ERK, ERK1/2, estrogen receptor, IKBKB, JINK1/2, Klrk1, Map3k7, MTOR, NFkB (complex), NLRP3, NOS2, P38 MAPK, RB1, TP53, TSC2 | ABAT, AKR1A1, ATL3, ATP6V1B2, CALR, CYP2E1, DLAT, GM2A, GSR, KRT8, LAP3, LDHB, Otub1, PDIA6, PLA2G7, PSMD2, RPL12, THOP1, UBE2N, YWHAH |
| KRAS | 5.67E-08 | Akt, AR, ATM, BAK1, BAX, CREB1, ERK, IKBKB, IKBKG, KRAS, Mek, MTOR, P38 MAPK, PGR, RB1, STK4, TCF7L2, TP53, TRIM28, TSC2 | ABAT, AKR1A1, ALAD, ATL3, ATP6V1B2, DLAT, GDI1, GM2A, GSR, GSTM1, GSTM3, KRT8, LDHB, Otub1, PDIA6, PDXK, PICALM, PLA2G7, PLS3, PSMD2, RPL12, THOP1, YWHAH |
| LGALS1 | 1.92E-05 | Akt, ATF6, CASP8, CHUK, ERK1/2, IKBKB, ITGB3, KDM5B, LGALS1, Mek, MKNK1, NLRP3, P38 MAPK, PPARG, Ras, REL, SP1, SYK, TCF7L2, TP53, TP63, TSC2 | ABAT, ACAD9, ATL3, ATP6V1B2, CALR, COTL1, DPYSL3, EPB41L2, GM2A, GSR, PDIA4, PICALM, PLA2G7, PLIN1, PLS3, PSMD2, THOP1, UBE2N, VIL1, YWHAH |
| LMNA | 1.41E-05 | Akt, IKBKB, LMNA, MTOR, NFkB (complex), NLRP3, P38 MAPK, PGR, RB1, TP53, TP73, TSC2 | ABAT, AKR1A1, ATL3, ATP6V1B2, DLAT, GM2A, GSR, GSTM3, KRT8, LCP1, LDHB, Otub1, PDIA6, PLA2G7, PSMD2, RPL12, THOP1, UBE2N, YWHAH |
| LYN | 4.22E-09 | Akt, BAX, BTK, CD44, CREB1, CTNNB1, ERK1/2, estrogen receptor, Fcer1, INPP5D, IRS1, Jnk, LYN, MAPK8, MKNK1, MTOR, NFE2L2, NFkB (complex), NLRP3, Pkc(s), RB1, REL, SYK, TGFB1, TP53, TSC2 | ABAT, ACAD9, AKR1A1, ATL3, ATP6V1B2, CALR, COTL1, CYP2E1, DLAT, DPYSL3, EPB41L2, GSR, GSTM1, KRT8, Otub1, PDIA4, PDIA6, PDXK, PSMD2, RPL12, SELENBP1, SERPIND1, THOP1, UBE2N, YWHAH |
| MAP3K5 | 1.58E-06 | CTNNB1, IKBKB, JINK1/2, Jnk, MAP3K5, MKNK1, NFE2L2, NOS2, NOS3, P38 MAPK, PGR, PPARG, TP53, TP63, TP73 | ABAT, ATL3, CYP2E1, DLAT, DPYSL3, EPB41L2, GM2A, GSR, GSTM1, GSTM3, KRT8, LAP3, LCP1, PDIA6, PLA2G7, PLIN1, PSMD2, SERPIND1, THOP1, VIL1, YWHAH |
| MAPK1 | 1.37E-06 | ATF6, CAV1, CREB1, EGFR, estrogen receptor, IKBKB, MAPK1, MAPK14, MTOR, NFkB (complex), NLRP3, PGR, TP53 | ABAT, AKR1A1, ATL3, CALR, GM2A, GSR, GSTM3, KRT8, LAP3, LDHB, Otub1, PDIA4, PDIA6, PDXK, PSMD2, PTRF, RPL12, THOP1, UBE2N, YWHAH |
| MAPK11 | 2.81E-02 | MAPK11 | CALR |
| MAPK14 | 2.79E-06 | Akt, AR, ATF6, ATR, CAV1, CBL, CDK1, CDK2, Creb, CREB1, CTNNB1, EGFR, ERK, Jnk, KDM5B, MAPK14, MKNK1, MTOR, NFE2L2, NFkB (complex), NLRP3, NOS2, P38 MAPK, PGR, PPARG, RB1, RPS6KA5, SP1, TFEB, TGFB1, TSC2 | AKR1A1, ATP6V1B2, CALR, CYP2E1, DLAT, DPYSL3, EPB41L2, GDI1, GSTM3, LDHB, Otub1, PDIA4, PDXK, PLA2G7, PLIN1, PLS3, PTRF, RPL12, SELENBP1, SERPIND1, UBE2N |
| Mek | 1.53E-05 | Akt, AXL, BAK1, BAX, BCL2L11, Creb, CREB1, CTNNB1, CXCR4, ELAVL1, ERK, ERK1/2, JUNB, KDR, Mapk, Mek, MKNK1, MTOR, MTORC1, NFkB (complex), NLRP3, P38 MAPK, PGR, PPARG, RB1, TCF7L2, TRAF2, TSC2, XBP1 | AKR1A1, ATP6V1B2, CALR, CYP2E1, DLAT, DPYSL3, EPB41L2, GSTM3, LAP3, Otub1, PDIA4, PDIA6, PDXK, PICALM, PLA2G7, PLIN1, PYGL, RPL12, Rrbp1, UBE2N |
| MIF | 3.01E-10 | Akt, CAV1, CREB1, CTNNB1, E2f, ERK1/2, HRAS, IKBKB, MAPK1, MIF, MTOR, NFE2L2, NFkB (complex), NLRP3, PGR, PRKCD, REL, SRC, SYK, TP53, TP73, TRAF2, TSC2 | ABAT, ACAD9, AKR1A1, ATL3, ATP6V1B2, CALR, COTL1, CYP2E1, GM2A, GSR, GSTM1, GSTM3, KRT8, LAP3, LCP1, LDHB, Otub1, PDIA4, PDIA6, PDXK, PSMD2, PTRF, PYGL, RPL12, SERPIND1, THOP1, UBE2N, YWHAH |
| MLIP | 1.71E-05 | Akt, BAK1, CREB1, CTNNB1, ERK1/2, MLIP, MTOR, PRKAA, PTEN, TP53, TSC2 | ABAT, AKR1A1, ATL3, ATP6V1B2, CALR, CYP2E1, DLAT, GSR, LAP3, LDHB, Otub1, PDIA6, PDXK, PSMD2, RPL12, THOP1, YWHAH |
| Mt1 | 4.87E-02 | Mt1 | GSTM3 |
| Mt2 | 4.65E-02 | Mt2 | GSTM3 |
| MTOR | 1.29E-06 | Akt, CREB1, Gsk3, IKBKB, IRS1, MTOR, PDPK1, PPARG, RB1, RHOA, SIRT1, TFEB, TP53, XBP1 | ABAT, AKR1A1, ATL3, ATP6V1B2, DLAT, GM2A, GSR, KRT8, LDHB, Otub1, PDIA4, PDIA6, PDXK, PLIN1, PSMD2, RPL12, Rrbp1, THOP1, YWHAH |
| MTOR | 5.17E-04 | MTOR | AKR1A1, LDHB, Otub1, RPL12 |
| MYD88 | 1.06E-05 | Akt, CHUK, ERK1/2, estrogen receptor, IKBKB, Jnk, MAP3K8, Mapk, MKNK1, MTOR, MYD88, NFkB (complex), NLRP3, NOS2, P38 MAPK, PGR, PI3K (complex), PPARG, STAT3, TFEB, TP53, TP63, TSC2 | ABAT, AKR1A1, ATL3, ATP6V1B2, CALR, CYP2E1, DPYSL3, EPB41L2, GM2A, GSTM3, KRT8, LDHB, Otub1, PLA2G7, PLIN1, PSMD2, RPL12, THOP1, UBE2N, VIL1, YWHAH |
| NFE2L2 | 8.66E-10 | 26s Proteasome, BRCA1, Creb, CREB1, CTNNB1, HMOX1, MKNK1, MTOR, NFE2L2, P38 MAPK, PGR, Pkc(s), PPARA, PPARG, RELA, TP53, TP63 | ABAT, AKR1A1, ATL3, CYP2E1, DLAT, DPYSL3, EPB41L2, GSR, GSTM1, GSTM3, KRT8, LDHB, Otub1, PCBP2, PDIA4, PDIA6, PDXK, PLA2G7, PLIN1, PSMD2, RPL12, SELENBP1, SERPIND1, THOP1, VIL1, YWHAH |
| NFE2L2 | 1.17E-04 | NFE2L2 | AKR1A1, GSR, GSTM1, PDIA4, PDIA6, SERPIND1 |
| NOS2 | 3.20E-08 | Akt, AR, BAX, CHUK, CREB1, estrogen receptor, FOXO1, GSK3B, IKBKB, IRS1, IRS2, ITGB2, Jnk, MMP9, MTOR, NFE2L2, NFkB (complex), NLRP3, NOS2, P38 MAPK, TFEB, TGFB1, TP53, TRAF2, TSC2 | ABAT, AKR1A1, ATL3, ATP6V1B2, CYP2E1, GDI1, GM2A, GSTM1, KRT8, LDHB, Otub1, PDIA4, PDIA6, PDXK, PLA2G7, PLS3, PSMD2, PYGL, RPL12, SELENBP1, SERPIND1, THOP1, UBE2N, YWHAH |
| NOS3 | 2.95E-08 | AMPK, BAX, CHUK, estrogen receptor, IKBKB, MAP2K1/2, MAPK3, MKNK1, MMP9, MTOR, NFE2L2, NFkB (complex), NLRP3, NOS2, NOS3, RB1, STAT3, TERT, TGFB1, TP53, TP63 | ABAT, AKR1A1, ATL3, CYP2E1, DLAT, DPYSL3, EPB41L2, GM2A, GSR, GSTM1, KRT8, LDHB, Otub1, PDIA4, PDIA6, PSMD2, RPL12, SELENBP1, SERPIND1, THOP1, UBE2N, VIL1, YWHAH |
| NOX4 | 9.88E-08 | Akt, AKT1, BAK1, BAX, CAV1, CHUK, ERK, ERK1/2, IKBKB, MAPK1, MAPK3, MTOR, N-cor, NFE2L2, NOS2, NOX4, P38 MAPK, PDPK1, RB1, SRC, STAT3, TP53, TP63, TRAF2, TSC2 | ABAT, ATL3, ATP6V1B2, CALR, CYP2E1, DLAT, GM2A, GSTM1, KRT8, LAP3, Otub1, PDIA4, PDIA6, PLA2G7, PLIN1, PSMD2, PTRF, PYGL, RPL12, SERPIND1, THOP1, VIL1, YWHAH |
| NPC1 | 5.11E-05 | ATF6, BAK1, BAX, IKBKB, MAPK1, MKNK1, MTOR, NPC1, NTRK2, P38 MAPK, PGR, PPARG, TP63 | AKR1A1, DLAT, DPYSL3, EPB41L2, GM2A, GSTM3, LAP3, LDHB, Otub1, PDIA4, PDIA6, PLA2G7, PLIN1, RPL12, VIL1 |
| NTRK1 | 5.07E-06 | Akt, ATF6, BAK1, BAX, CREB1, IKBKB, MAPK1, MAPK3, MKNK1, MTOR, NFE2L2, NTRK1, P38 MAPK, PGR, PPARG, TP63, TSC2 | AKR1A1, ATP6V1B2, DPYSL3, EPB41L2, GM2A, GSR, GSTM1, GSTM3, LAP3, LDHB, Otub1, PDIA4, PDIA6, PDXK, PLA2G7, PLIN1, RPL12, SERPIND1, VIL1 |
| OSM | 4.45E-09 | AKT1, ATF6, CAV1, CDK2, CHUK, HOXA10, JAK1, JAK2, MAPK1, MAPK14, MKNK1, MTOR, N-cor, OSM, PGR, RB1, SHC1, STAT1, STAT3, TP53, TP63, TSC2 | ABAT, AKR1A1, ATL3, ATP6V1B2, CALR, CYP2E1, DLAT, DPYSL3, EPB41L2, GM2A, GSR, GSTM3, KRT8, LAP3, Otub1, PDIA4, PDIA6, PLIN1, PSMD2, PTRF, RPL12, THOP1, VIL1, YWHAH |
| P38 MAPK | 1.15E-07 | Akt, AR, ATF6, BAK1, BAX, CDK1, CREB1, EGFR, Hsp27, IKBKB, MKNK1, MMP9, MTOR, NFE2L2, NFkB (complex), NLRP3, P38 MAPK, PGR, PPARG, TFEB, TGFB1, TP63, TRAF2, TSC2 | AKR1A1, ATP6V1B2, DLAT, DPYSL3, EPB41L2, GDI1, GM2A, GSTM1, GSTM3, LDHB, Otub1, PDIA4, PDIA6, PDXK, PLA2G7, PLIN1, PLS3, PYGL, RPL12, SELENBP1, SERPIND1, UBE2N, VIL1 |
| P38 MAPK | 6.34E-05 | ATF6, BAK1, BAX, IKBKB, MKNK1, MTOR, P38 MAPK, PGR, PPARG, TP63 | AKR1A1, DLAT, DPYSL3, EPB41L2, GM2A, GSTM3, LDHB, Otub1, PDIA4, PDIA6, PLA2G7, PLIN1, RPL12, VIL1 |
| PBK | 1.03E-06 | BAK1, BAX, CAV1, DUSP1, H2AFX, IKBKB, MAPK1, MAPK14, MAPK8, MKNK1, P38 MAPK, PBK, PGR, PPARG, RB1, TP53, TP63 | ABAT, ATL3, CALR, DLAT, DPYSL3, EPB41L2, GM2A, GSR, GSTM3, KRT8, LAP3, PDIA6, PLA2G7, PLIN1, PSMD2, PTRF, THOP1, VIL1, YWHAH |
| Pdgf (complex) | 2.08E-06 | AKT1, CHUK, HOXA10, IKBKB, JAK2, MTOR, N-cor, NFKBIA, NOS2, P38 MAPK, Pdgf (complex), PI3K (complex), RB1, TP53, TSC2 | ABAT, AKR1A1, ATL3, ATP6V1B2, CYP2E1, DLAT, GM2A, GSR, KRT8, Otub1, PDIA6, PLA2G7, PLIN1, PSMD2, RPL12, THOP1, YWHAH |
| PFN1 | 2.76E-05 | ATF6, BAK1, BAX, IKBKB, MKNK1, MTOR, NOS2, NOS3, P38 MAPK, PFN1, PGR, PPARG, TP63 | AKR1A1, CYP2E1, DLAT, DPYSL3, EPB41L2, GM2A, GSTM3, Otub1, PDIA4, PDIA6, PLA2G7, PLIN1, RPL12, VIL1 |
| PIK3CA | 5.28E-06 | Akt, AR, BAK1, CHUK, CREB1, CTNNB1, ERK1/2, FOXO1, GSK3B, IRS1, MTOR, MYC, PIK3CA, PRKAA, RB1, TP53, TSC2 | ABAT, AKR1A1, ATL3, CALR, CYP2E1, DLAT, GDI1, GM2A, GSR, GSTM1, KRT8, LDHB, Otub1, PDIA6, PDXK, PLS3, PSMD2, RPL12, THOP1, YWHAH |
| PKD1 | 9.17E-03 | AMPK, CTNNB1, PKD1 | CYP2E1, LAP3, LDHB, PICALM, PTRF |
| PLAT | 3.86E-05 | ATF6, BAK1, BAX, CASP3, CASP8, ERK1/2, IKBKB, MKNK1, MTOR, NLRP3, P38 MAPK, PGR, PLAT, PPARG, PRKACA, TFEB, TGM2, TP63 | AKR1A1, ATP6V1B2, CALR, DPYSL3, EPB41L2, GM2A, GSTM3, LDHB, NLN, Otub1, PDIA4, PDIA6, PLA2G7, PLIN1, RPL12, UBE2N, VIL1 |
| PLAU | 3.02E-07 | CREB1, CTNNB1, EGFR, ERK1/2, MMP9, MTOR, P38 MAPK, PGR, PLAU, PLG, PPARG, PTK2, RB1, STAT3, TGFB1, TGM2, TP53, TP63, TSC2 | ABAT, AKR1A1, ATL3, ATP6V1B2, CALR, CYP2E1, DLAT, GSR, GSTM3, KRT8, LDHB, NLN, Otub1, PDIA6, PDXK, PLA2G7, PLIN1, PSMD2, RPL12, SELENBP1, THOP1, VIL1, YWHAH |
| PPARA | 1.85E-03 | PPARA | GSTM3, KRT8, PLA2G7, PLIN1, SELENBP1 |
| PPARD | 3.18E-07 | AKT1, AR, BAK1, BAX, CASP3, CREB1, CTNNB1, ERK1/2, FOXO1, GSK3B, MAP2K1/2, MKNK1, MTOR, Pkc(s), PPARD, PRKAA, PRKCA, RAF1, STAT3, TCF7L2, TP53, TP63 | ABAT, AKR1A1, ATL3, CALR, CYP2E1, DPYSL3, EPB41L2, GDI1, GSR, GSTM1, KRT8, LAP3, LDHB, Otub1, PDIA6, PDXK, PICALM, PLS3, PSMD2, RPL12, THOP1, VIL1, YWHAH |
| PPARG | 1.16E-02 | PPARG | DLAT, HEBP1, PLIN1, PYGL |
| PPARG | 8.83E-07 | AKT1, APP, Creb, CREB1, CTNNB1, IRS1, MKNK1, MMP9, MTOR, NFE2L2, NFkB (complex), NFKBIA, NLRP3, P38 MAPK, PGR, PPARG, STAT3, TGFB1, TP63, TP73, TSC2 | AKR1A1, ATP6V1B2, CYP2E1, DLAT, DPYSL3, EPB41L2, GSR, GSTM1, GSTM3, LCP1, Otub1, PDIA4, PDIA6, PDXK, PLA2G7, PLIN1, RPL12, SELENBP1, SERPIND1, UBE2N, VIL1 |
| Prl3d1 (includes others) | 8.33E-05 | CHUK, CREB1, CTNNB1, ERK1/2, MAPK1, NFE2L2, P38 MAPK, PRKCD, Prl3d1 (includes others), RB1, STAT3, TP63, TRAF2 | AKR1A1, CALR, CYP2E1, DLAT, GM2A, GSR, GSTM1, LAP3, PDIA4, PDIA6, PDXK, PLA2G7, PYGL, SERPIND1, VIL1 |
| PRMT1 | 8.61E-06 | ATF6, DSP, HNF4A, IKBKB, MKNK1, NFkB (complex), NLRP3, P38 MAPK, PGR, PPARG, PRMT1, TP53, TP63, TP73 | ABAT, ATL3, DLAT, DPYSL3, EPB41L2, GM2A, GSR, GSTM3, KRT8, LCP1, PDIA4, PDIA6, PLA2G7, PLIN1, PSMD2, PYGL, THOP1, UBE2N, VIL1, YWHAH |
| PROC | 7.89E-08 | ATF6, BAK1, BAX, CASP3, CASP8, CREB1, EGFR, estrogen receptor, IKBKB, MAPK1, MKNK1, MTOR, NFkB (complex), NLRP3, P38 MAPK, PPARG, PROC, RB1, TP53, TP63 | ABAT, AKR1A1, ATL3, DLAT, DPYSL3, EPB41L2, GM2A, GSR, KRT8, LAP3, LDHB, Otub1, PDIA4, PDIA6, PDXK, PLA2G7, PLIN1, PSMD2, RPL12, THOP1, UBE2N, VIL1, YWHAH |
| PSEN2 | 2.55E-05 | AKT1, ERK1/2, MAPK1, MTOR, N-cor, P38 MAPK, PGR, PLA2G6, PSEN2, SERCA, TP53, TP73, TSC2 | ABAT, AKR1A1, ATL3, ATP6V1B2, CALR, GSR, GSTM3, KRT8, LAP3, LCP1, Otub1, PDIA6, PLA2G7, PLIN1, PSMD2, RPL12, THOP1, YWHAH |
| PTEN | 1.58E-02 | PTEN | DLAT, LDHB, VIL1 |
| PTEN | 3.27E-04 | CREB1, CTNNB1, ERK1/2, estrogen receptor, MTOR, P38 MAPK, PTEN, TSC2 | AKR1A1, ATP6V1B2, CALR, CYP2E1, DLAT, KRT8, LAP3, LDHB, Otub1, PDXK, PLA2G7, RPL12 |
| PTGIS | 4.74E-03 | PTGIS | CYP2E1 |
| REN | 1.24E-05 | CREB1, ERK, ERK1/2, estrogen receptor, IKBKB, MTOR, NFkB (complex), NLRP3, REN, TP53, XDH | ABAT, AKR1A1, ATL3, CALR, CYP2E1, GM2A, GSR, KRT8, LDHB, Otub1, PDIA6, PDXK, PSMD2, RPL12, THOP1, UBE2N, YWHAH |
| REN | 4.65E-02 | REN | CYP2E1 |
| RGS6 | 1.72E-07 | ATM, CREB1, IKBKB, IKBKG, MAPK1, MAPK14, MDM2, MTOR, PGR, RB1, RGS6, TP53, TP73, TRIM28 | ABAT, AKR1A1, ALAD, ATL3, CALR, DLAT, GM2A, GSR, GSTM3, KRT8, LAP3, LCP1, LDHB, Otub1, PDIA6, PDXK, PSMD2, RPL12, THOP1, YWHAH |
| RHOA | 2.90E-08 | CTNNB1, IKBKB, Integrin, Jnk, MKNK1, MTOR, NFE2L2, P38 MAPK, PPARG, RHOA, ROCK1, TP53, TP63, TP73, TSC2 | ABAT, ATL3, ATP6V1B2, CYP2E1, DLAT, DPYSL3, EPB41L2, GM2A, GSR, GSTM1, KRT8, LAP3, LCP1, LDHB, Otub1, PDIA6, PLA2G7, PLIN1, PSMD2, RPL12, SERPIND1, THOP1, VIL1, YWHAH |
| RHOA | 1.22E-03 | P38 MAPK, RHOA, TP53 | ABAT, ATL3, GSR, KRT8, PDIA6, PLA2G7, PSMD2, THOP1, YWHAH |
| RORC | 4.04E-02 | RORC | CYP2E1, SELENBP1 |
| SELE | 3.32E-11 | AR, ATF6, ERK, ERK1/2, Hsp27, IKBKB, MAPK1, MKNK1, MTOR, NOS2, P38 MAPK, PGR, PPARG, SELE, TP53, TP63 | ABAT, AKR1A1, ATL3, CALR, CYP2E1, DLAT, DPYSL3, EPB41L2, GDI1, GM2A, GSR, GSTM1, GSTM3, KRT8, LAP3, LDHB, Otub1, PDIA4, PLA2G7, PLIN1, PLS3, PSMD2, RPL12, THOP1, VIL1, YWHAH |
| SERPINE1 | 5.10E-02 | SERPINE1 | CALR |
| SERPINH1 | 1.05E-05 | Akt, ERK1/2, MTOR, NFKBIA, P38 MAPK, PI3K (family), RB1, SERPINH1, TP53, TSC2 | ABAT, AKR1A1, ATL3, ATP6V1B2, CALR, DLAT, GSR, KRT8, LDHB, Otub1, PDIA6, PLA2G7, PSMD2, RPL12, THOP1, YWHAH |
| SGCD | 9.47E-03 | SGCD | DAG1 |
| SMAD7 | 7.77E-02 | SMAD7 | KRT8 |
| SOCS3 | 4.43E-07 | Akt, AKT1, CASP8, CAV1, CFTR, ERK1/2, ERN1, HOXA10, JAK1, JAK2, LEPR, MKNK1, MTOR, N-cor, NFKBIA, NLRP3, Pka, RB1, RHO, SOCS3, STAT3, TP53, TP63, TSC2 | ABAT, AKR1A1, ATL3, ATP6V1B2, CALR, CRABP1, CYP2E1, DLAT, DPYSL3, EPB41L2, GSR, KRT8, Otub1, PLIN1, PSMD2, PTRF, RPL12, THOP1, UBE2N, VIL1, YWHAH |
| SOD1 | 7.46E-03 | SOD1 | DPYSL3, GSTM1, GSTM3 |
| SREBF1 | 4.66E-02 | SREBF1 | GSR, PLS3 |
| SYVN1 | 2.33E-05 | HTT, MAPK14, MTOR, PGR, SYVN1, TP53, TP73 | ABAT, AKR1A1, ATL3, CALR, DAG1, GSR, GSTM3, KRT8, LCP1, LDHB, Otub1, PDIA6, PSMD2, PTRF, RPL12, THOP1, YWHAH |
| SYVN1 | 9.43E-05 | SYVN1, TP53 | ABAT, ATL3, DAG1, GSR, KRT8, LDHB, PDIA6, PSMD2, PTRF, THOP1, YWHAH |
| SYVN1 | 4.07E-03 | SYVN1 | DAG1, LDHB, PTRF |
| TBXA2R | 3.36E-04 | Histone h3, NLRP3, P38 MAPK, RHOA, TBXA2R, TP53 | ABAT, ATL3, GSR, KRT8, PDIA6, PLA2G7, PSMD2, THOP1, UBE2N, YWHAH |
| TERC | 4.88E-05 | MAPK14, MTOR, PGR, RB1, TERC, TERT, TP53, TP73 | ABAT, AKR1A1, ATL3, CALR, DLAT, GSR, GSTM3, KRT8, LCP1, LDHB, Otub1, PDIA6, PSMD2, RPL12, THOP1, YWHAH |
| TGFB1 | 2.19E-07 | ABL1, Akt, AKT1, AR, ATM, CAV1, CTNNB1, FMR1, Hsp27, MAP2K1/2, MAPK14, MKNK1, NFkB (complex), NLRP3, P38 MAPK, PPARG, RICTOR, RPS6KB1, SRC, STAT3, TCF7L2, TGFB1, TGM2, TP53, TP63, TRIM28, TSC2 | ABAT, ALAD, ATL3, ATP6V1B2, CALR, CYP2E1, DAG1, DLAT, DPYSL3, EPB41L2, GDI1, GM2A, GSR, KRT8, NLN, PDIA6, PICALM, PLA2G7, PLIN1, PLS3, PSMD2, PTRF, SELENBP1, THOP1, UBE2N, VIL1, YWHAH |
| THBS4 | 5.10E-02 | THBS4 | CALR |
| THBS4 | 7.59E-03 | ATF6, THBS4 | CALR, PDIA4 |
| TIMP1 | 5.35E-06 | ATF6, CAV1, CHUK, IKBKB, MAP2K6, MAPK1, MAPK14, MAPK3, MTOR, NFE2L2, NOS2, P38 MAPK, PI3K (complex), PTK2, RB1, TIMP1, TSC2 | AKR1A1, ATP6V1B2, CALR, CYP2E1, DLAT, GM2A, GSR, GSTM1, LAP3, LDHB, Otub1, PDIA4, PDIA6, PLA2G7, PTRF, RPL12, SERPIND1 |
| TNF | 4.61E-02 | TNF | COTL1, CYP2E1, GM2A, PLIN1, SERPIND1 |
| TNFAIP8L2 | 5.35E-07 | Akt, ERK1/2, IKBKB, Jnk, MKNK1, MTOR, MYC, NFE2L2, P38 MAPK, PGR, PPARG, Rac, RALA, TNFAIP8L2, TP53, TP63, TSC2 | ABAT, ATL3, ATP6V1B2, CALR, DPYSL3, EPB41L2, GM2A, GSR, GSTM1, GSTM3, KRT8, LDHB, Otub1, PLA2G7, PLIN1, PSMD2, RPL12, SERPIND1, THOP1, VIL1, YWHAH |
| TNFRSF17 | 1.90E-06 | ERK1/2, estrogen receptor, IKBKB, Jnk, MKNK1, MTOR, NFE2L2, NFkB (complex), NLRP3, P38 MAPK, PGR, PPARG, TNFRSF17, TP53, TP63 | ABAT, ATL3, CALR, DLAT, DPYSL3, EPB41L2, GM2A, GSTM1, GSTM3, KRT8, LDHB, Otub1, PLA2G7, PLIN1, PSMD2, RPL12, SERPIND1, THOP1, UBE2N, VIL1, YWHAH |
| TNFRSF1A | 3.47E-06 | CASP8, CAV1, CD44, ERK1/2, estrogen receptor, Gsk3, IKBKB, Jnk, MAPK14, MKNK1, NFkB (complex), NFKB1, NLRP3, P38 MAPK, PGR, PPARG, PRKCD, RB1, RELA, TFEB, TGFB1, TNFRSF1A, TP53, TP63, TRAF2 | ABAT, ATL3, ATP6V1B2, CALR, DLAT, DPYSL3, EPB41L2, GM2A, GSTM3, KRT8, PLA2G7, PLIN1, PSMD2, PTRF, PYGL, SELENBP1, THOP1, UBE2N, VIL1, YWHAH |
| TNFRSF1B | 2.70E-06 | CASP8, ERK1/2, estrogen receptor, IKBKB, Jnk, MKNK1, MTOR, NFE2L2, NFkB (complex), NLRP3, P38 MAPK, PGR, PPARG, RELA, TNFRSF1B, TP53, TP63 | ABAT, ATL3, CALR, DLAT, DPYSL3, EPB41L2, GM2A, GSTM1, GSTM3, KRT8, LDHB, Otub1, PLA2G7, PLIN1, PSMD2, RPL12, SERPIND1, THOP1, UBE2N, VIL1, YWHAH |
| TNFRSF25 | 1.85E-09 | ATF6, BAK1, BAX, ERK, ERK1/2, estrogen receptor, IKBKB, MAPK1, MKNK1, MTOR, NFkB (complex), NFKB1, NLRP3, NOS2, P38 MAPK, PGR, PPARG, TNFRSF25, TP53, TP63 | ABAT, AKR1A1, ATL3, CALR, CYP2E1, DLAT, DPYSL3, EPB41L2, GM2A, GSR, GSTM3, KRT8, LAP3, LDHB, Otub1, PDIA4, PDIA6, PLA2G7, PLIN1, PSMD2, RPL12, THOP1, UBE2N, VIL1, YWHAH |
| TNFRSF9 | 2.35E-08 | ATF6, BAK1, BAX, estrogen receptor, IKBKB, MKNK1, MTOR, NFkB (complex), NLRP3, P38 MAPK, PGR, PPARG, TNFRSF9, TP53, TP63 | ABAT, AKR1A1, ATL3, DLAT, DPYSL3, EPB41L2, GM2A, GSR, GSTM3, KRT8, LDHB, Otub1, PDIA4, PDIA6, PLA2G7, PLIN1, PSMD2, RPL12, THOP1, UBE2N, VIL1, YWHAH |
| TNFSF10 | 3.81E-08 | Akt, AMPK, BAK1, BAX, CASP8, CAV1, CREB1, ERK1/2, ERN1, estrogen receptor, IKBKB, Jnk, MAP3K7, MAPK1, MKNK1, MTOR, NFkB (complex), NLRP3, P38 MAPK, PGR, Pkc(s), PPARG, PRKCE, RELA, SRC, TAB2, TNFSF10, TP53, TP63, TRAF2, TSC2 | ABAT, ATL3, ATP6V1B2, CALR, CYP2E1, DPYSL3, EPB41L2, GM2A, GSTM3, KRT8, LAP3, LDHB, Otub1, PDIA6, PDXK, PLA2G7, PLIN1, PSMD2, PTRF, PYGL, RPL12, THOP1, UBE2N, VIL1, YWHAH |
| TNFSF12 | 3.43E-06 | AMPK, AR, CASP8, CHUK, CTNNB1, ERK1/2, ERN1, estrogen receptor, FMR1, FOXO1, GSK3B, IKBKB, Jnk, MAPK1, MKNK1, MTOR, NFkB (complex), NLRP3, P38 MAPK, PGR, PPARG, RPS6KB1, TNFSF12, TP53, TP63 | ABAT, AKR1A1, ATL3, CALR, CYP2E1, DAG1, DPYSL3, EPB41L2, GDI1, GM2A, GSTM3, KRT8, LAP3, LDHB, Otub1, PLA2G7, PLIN1, PLS3, RPL12, THOP1, UBE2N, VIL1, YWHAH |
| TNFSF15 | 1.70E-06 | CHUK, ERK1/2, estrogen receptor, IKBKB, IKBKG, Jnk, MAPK1, MKNK1, MTOR, NFE2L2, NFkB (complex), NFKB1, NLRP3, P38 MAPK, PGR, PPARG, RIPK1, STAT3, TNFSF15, TP53, TP63 | ABAT, ATL3, CALR, DLAT, DPYSL3, EPB41L2, GM2A, GSTM1, GSTM3, KRT8, LAP3, LDHB, Otub1, PLA2G7, PLIN1, PSMD2, RPL12, SERPIND1, THOP1, UBE2N, VIL1, YWHAH |
| TP53 | 5.23E-03 | TP53 | ABAT, ATL3, GSR, KRT8, PDIA6, PSMD2, THOP1, YWHAH |
| TP53 | 1.26E-07 | AR, BBC3, CAV1, CCND1, CDK1, E2F3, FMR1, GRIN1, IGF1R, Jnk, MAPK14, Mek, MKNK1, MTOR, NFE2L2, PGR, PIK3R1, STAT3, TCF7L2, TP53, TP63, TP73, XBP1 | ABAT, AKR1A1, ATL3, CALR, DAG1, DPYSL3, EPB41L2, GDI1, GSR, GSTM1, GSTM3, KRT8, LCP1, LDHB, Otub1, PDIA6, PICALM, PLS3, PSMD2, PTRF, RPL12, Rrbp1, SERPIND1, THOP1, VIL1, YWHAH |
| TP53 | 1.13E-04 | MAPK14, MTOR, PGR, TP53, TP73 | ABAT, AKR1A1, ATL3, CALR, GSR, GSTM3, KRT8, LCP1, LDHB, Otub1, PDIA6, PSMD2, RPL12, THOP1, YWHAH |
| TSLP | 9.30E-06 | ELANE, ERK1/2, IKBKB, IL1B, Jnk, MAPK1, MKNK1, MTOR, NFE2L2, P38 MAPK, PGR, PPARG, TP63, TSLP | CALR, CYP2E1, DLAT, DPYSL3, EPB41L2, GM2A, GSR, GSTM1, GSTM3, LAP3, LCP1, LDHB, Otub1, PLA2G7, PLIN1, RPL12, SERPIND1, VIL1 |
| VAV2 | 2.43E-05 | CHUK, ERK, ERK1/2, MTOR, NOS2, PLCG2, RAC1, RHOA, STAT3, TP53, TP63, VAV2 | ABAT, AKR1A1, ATL3, CALR, CYP2E1, GM2A, GSR, KRT8, LDHB, Otub1, PDIA6, PSMD2, RPL12, THOP1, VIL1, YWHAH |
| Vegf | 1.08E-02 | Vegf | CRABP1, NPL |
| WNT5A | 3.56E-06 | CTNNB1, ERK, IKBKB, Jnk, MTOR, MYC, NFATC2, NFE2L2, P38 MAPK, TCF7L2, TP53, WNT5A | ABAT, ATL3, CYP2E1, GM2A, GSR, GSTM1, KRT8, LAP3, LCP1, LDHB, Otub1, PDIA4, PDIA6, PICALM, PLA2G7, PSMD2, RPL12, SERPIND1, THOP1, YWHAH |
| **Alb-SREBP-1c** |  |  |  |
| **Master Regulator** | **p-value of overlap** | **Participating regulators** | **Target molecules in dataset** |
| ADIPOQ | 2.72E-07 | ADIPOQ, Akt, AMPK, CTNNB1, estrogen receptor, IGF1R, Jnk, MKNK1, MTOR, MYC, NFE2L2, NFkB (complex), NOS2, P38 MAPK, PI3K (complex), PRKAA1, PRKAA2, RELA, RHOA, STK11, TCF7L2, TP53, USP8 | ACACA, ACTC1, ACTN4, ECH1, EEF1D, FBP2, FDPS, GSTM1, HNRNPD, IDH3A, ITIH4, ITIH5, LDHB, MAPK14, MSN, PAFAH1B2, PKM, RPLP0, SERPINH1, SNX5, SORBS1, TOP2B |
| ADORA2A | 2.37E-06 | ADCY, ADORA2A, AMPK, CASP1, CFTR, Creb, estrogen receptor, Mapk, MAPK1, MAPK3, MKNK1, NFE2L2, NFkB (complex), P38 MAPK, PGR, Pka, RARA, RHO, STAT1, TP53 | ACACA, ACTN4, CTSB, ECH1, EEF1D, GNB1, GSTM1, HNRNPD, IDH3A, MSN, NQO2, PAFAH1B2, POR, PSME1, RPLP0, SERPINH1, SNX5, SORBS1, TGM2, TOP2B |
| ADORA2B | 1.52E-07 | ADORA2B, Akt, CEBPA, estrogen receptor, IGF1R, MKNK1, NFkB (complex), P38 MAPK, PGR, PTEN, RB1, SRC, STAT1, TP53, USP8 | ACACA, ACTN4, ECH1, EEF1D, HNRNPD, ICAM1, IDH3A, LDHB, MAPK14, MSN, NQO2, PAFAH1B2, PGD, PSME1, SNX5, SORBS1, SUCLG2, TGM2, TOP2B, Tpm1 |
| ADRB1 | 4.71E-04 | ADRB1 | ACTC1, Tpm1 |
| AGTR1 | 3.23E-06 | AGTR1, Akt, CEBPA, ERK1/2, estrogen receptor, FAS, IRS1, Jnk, Mapk, MKNK1, MTORC1, NFE2L2, NFkB (complex), PRKAA1, RB1, RELA, SREBF2, TP53, TSC2, USP8 | ACACA, ACTC1, ACTN4, COL15A1, ECH1, EEF1D, FDPS, GSTM1, HNRNPD, ICAM1, ITIH5, LDHB, MSN, PAFAH1B2, RPLP0, SNX5, SORBS1, TGM2, TOP2B |
| ATF3 | 7.19E-07 | Akt, ATF3, ERK, FAS, Jnk, MKNK1, MTOR, MTORC1, NFE2L2, NOS2, P38 MAPK, PGR, RB1, STAT1, TP53, USP8 | ACTC1, ACTN4, COL15A1, ECH1, EEF1D, GSTM1, HNRNPD, ICAM1, ITIH4, NQO2, PAFAH1B2, PGD, PKM, PSME1, RPLP0, SNX5, SORBS1, TGM2, TOP2B, Tpm1 |
| BAX | 4.45E-03 | BAX | CTSB, TGM2 |
| BAX | 7.85E-07 | BAX, CASP1, CASP8, FAS, HIF1A, IRF3, Jnk, JUN, MAP3K5, MTORC1, NFE2L2, SREBF1, STAT1, TP53, TRAF2 | ACACA, ACTN4, COL15A1, CTSB, ECH1, FDPS, GSTM1, ICAM1, PAFAH1B2, PKM, PSME1, RPLP0, SERPINH1, SNX5, SORBS1, TGM2, TOP2B, Tpm1 |
| BCL6 | 1.93E-06 | AR, BCL6, CDKN2A, CEBPA, E2F1, estrogen receptor, FAS, IGF1R, Jnk, MKNK1, NFE2L2, NFkB (complex), P38 MAPK, PGR, STAT1, TP53 | ACTN4, COL15A1, CTSB, ECH1, EEF1D, GSTM1, ICAM1, IDH3A, MAPK14, MSN, NQO2, PAFAH1B2, PGD, PSME1, RPLP0, SERPINH1, SNX5, SORBS1, TGM2, TOP2B, Tpm1 |
| BECN1 | 4.85E-02 | BECN1 | VPS29 |
| BID | 3.61E-02 | BID | CTSB |
| C1QTNF9 | 9.09E-06 | 26s Proteasome, ACACB, C1QTNF9, E2F1, ERK, FYN, MTOR, NOS2, PRKAA2 | ACACA, ACTC1, CTSB, FDPS, ICAM1, IDH3A, ITIH4, ITIH5, LDHB, NQO2, PKM, SERPINH1, TGM2 |
| CAPNS1 | 3.16E-07 | Akt, CAPNS1, CEBPA, estrogen receptor, FAS, HIF1A, Jnk, MTORC1, NFE2L2, NFkB (complex), RB1, SREBF2, TP53, TSC2, USP8 | ACACA, ACTC1, ACTN4, COL15A1, ECH1, FDPS, GSTM1, HNRNPD, ICAM1, LDHB, MSN, PAFAH1B2, PKM, RPLP0, SERPINH1, SNX5, SORBS1, TGM2, TOP2B |
| CAV1 | 2.00E-07 | AMPK, c-Src, CAV1, ERK1/2, estrogen receptor, GSK3B, IGF1R, IRS1, Jnk, MAPK3, Mek, NFE2L2, NOS2, NOS3, PGR, PRKAA1, PRKCA, PSEN1, RB1, SHC1, SRC, STAT1, STAT5A, TCF7L2, TP53 | ACACA, ACTC1, ACTN4, ECH1, FBP2, GNB1, GSTM1, IDH3A, ITIH4, ITIH5, LDHB, MAPK14, MSN, NQO2, PAFAH1B2, PGD, PSME1, RPLP0, SERPINH1, SNX5, SORBS1, TOP2B |
| CAV3 | 6.80E-06 | Akt, AKT1, CAV3, CTNNB1, ERK1/2, GATA1, GSK3B, INSR, IRS1, MYC, PRKAA1, PSEN1, RB1, STAT1, TP53, USP8 | ACACA, ACTC1, ACTN4, COL15A1, ECH1, FDPS, GNB1, HNRNPD, ICAM1, IDH3A, ITIH5, PAFAH1B2, PGD, PSME1, SNX5, SORBS1, TOP2B, Tpm1 |
| CBL | 4.05E-07 | AKT1, AMPK, CBL, EGFR, ERK, FAS, IGF1R, MAPK1, MAPK14, MAPK3, MTOR, MYC, NFE2L2, NOS2, PI3K (complex), PRKAA, PRKAA1, RARA, TGFB1, TGM2, TP53 | ACTN4, COL15A1, CTSB, ECH1, FBLN5, FDPS, GSTM1, HNRNPD, IDH3A, ITIH4, ITIH5, LDHB, MAPK14, PAFAH1B2, PKM, POR, RPLP0, SERPINH1, SNX5, SORBS1, TOP2B, Tpm1 |
| CCR5 | 1.76E-06 | BID, CASP1, CASP8, CCR5, CEBPA, CXCR3, estrogen receptor, HIF1A, MAPK14, Mek, MTORC1, NFkB (complex), PLD1, SRC, TP53, ZAP70 | ACACA, ACTN4, CTSB, ECH1, ICAM1, MSN, NQO2, PAFAH1B2, PGD, PKM, SERPINH1, SNX5, SORBS1, TGM2, TOP2B, Tpm1 |
| CD19 | 6.83E-07 | AMPK, BTK, CD19, ERK1/2, FAS, FYN, HIF1A, Jnk, LYN, MAP2K1, MAPK1, MAPK8, MAPK9, MTOR, MTORC1, NFE2L2, NOS2, PRKAA1, RARA, STAT1, TP53 | ACACA, ACTC1, ACTN4, COL15A1, CTSB, ECH1, GSTM1, HNRNPD, ICAM1, ITIH4, ITIH5, LDHB, NQO2, PAFAH1B2, PKM, PSME1, RPLP0, SNX5, SORBS1, TGM2, TOP2B |
| CD44 | 1.76E-08 | CD44, CTNNB1, EGFR, ERK, ESR1, ESR2, FAS, GSK3B, INSR, ITGB2, MAPK1, MAPK3, MKNK1, MYC, NFE2L2, NOS2, P38 MAPK, PRKAA1, PRKCE, PSEN1, Rac, REL, SRC, STAT1, SYK, TCF7L2, TGFB1, TGM2, TP53 | ACTC1, ACTN4, COL15A1, CTSB, ECH1, EEF1D, FBLN5, FBP2, GNB1, GSTM1, HNRNPD, IDH3A, ITIH4, ITIH5, NQO2, PAFAH1B2, PLEC, POR, PSME1, RPLP0, SERPINH1, SNX5, SORBS1, TGM2, TOP2B |
| CEBPB | 3.21E-06 | ACLY, ATM, CASP1, CEBPB, INSR, NFkB (complex), PI3K (complex), TP53 | ACACA, ACTN4, CTSB, ECH1, FDPS, ICAM1, IDH3A, PAFAH1B2, SERPINH1, SNX5, SORBS1, TGM2, TOP2B, Tpm1 |
| CFTR | 1.09E-02 | CFTR | FDPS, POR |
| CFTR | 2.51E-05 | BID, CFTR, Mapk, MKNK1, MTOR, NFKBIA, RB1 | ACTC1, CTSB, EEF1D, FDPS, ICAM1, LDHB, PGD, PKM, POR, SERPINH1, Tpm1 |
| CNR2 | 5.46E-06 | AMPK, CNR2, ERK, ERN1, MAPK1, MAPK14, MTOR, MTORC1, NOS2, RAC2, RB1, RHOA, TP53 | ACACA, ACTC1, ACTN4, CTSB, ECH1, FDPS, ICAM1, IDH3A, ITIH4, LDHB, PAFAH1B2, PGD, SNX5, SORBS1, TGM2, TOP2B |
| CNR2 | 7.98E-04 | AMPK, CNR2, ERK | ICAM1, IDH3A, LDHB, TGM2 |
| COL18A1 | 1.24E-06 | Akt, COL18A1, CTNNB1, estrogen receptor, ETS1, FAS, ID1, IGF1R, Jnk, MAPK1, MTOR, PTEN, RB1, RELA, SRC, STAT1 | ACACA, ACTC1, COL15A1, CTSB, EEF1D, FDPS, ICAM1, IDH3A, LDHB, MAPK14, MSN, NQO2, PGD, PKM, PSME1, SUCLG2, TGM2 |
| COL18A1 | 1.11E-05 | COL18A1, ETS1, Jnk, MAPK1, MTOR, PTEN, RELA | ACACA, CTSB, EEF1D, FDPS, ICAM1, IDH3A, LDHB, PKM, SUCLG2, TGM2 |
| COL4A3 | 4.85E-02 | COL4A3 | MAPK14 |
| CREM | 2.40E-02 | CREM | ACTC1, Tpm1 |
| CTGF | 9.36E-08 | Akt, Creb, CTGF, ERK, IGF1R, IRS1, MAPK14, MTOR, MYC, NOS2, PPARG, PRKAA1, PTK2, RB1, SREBF2, STAT1, TP53, TSC2, USP8 | ACACA, ACTN4, COL15A1, CTSB, ECH1, FDPS, HNRNPD, ICAM1, ITIH4, ITIH5, LDHB, MAPK14, PAFAH1B2, PGD, PKM, PSME1, SNX5, SORBS1, TGM2, TOP2B, Tpm1 |
| CX3CL1 | 3.19E-02 | CX3CL1 | ICAM1 |
| CXCL10 | 1.37E-06 | AKT1, AMPK, CXCL10, ERK1/2, MAPK1, MAPK14, Mek, MTORC1, NOS2, PI3K (complex), PRKAA1, RARA, RB1, Rsk, STAT1, TCF7L2, TP53 | ACTN4, CTSB, ECH1, FBP2, FDPS, HNRNPD, ICAM1, IDH3A, ITIH4, ITIH5, PAFAH1B2, PGD, PKM, PSME1, SERPINH1, SNX5, SORBS1, TGM2, TOP2B, Tpm1 |
| DCN | 4.65E-02 | DCN | ICAM1 |
| DYSF | 1.24E-02 | DYSF | ETF1, IDH3A |
| EGFR | 2.86E-02 | EGFR | ICAM1, TGM2 |
| EGR1 | 1.04E-04 | EGR1, MAPK14, MMP9 | CTSB, FDPS, ICAM1, TGM2, Tpm1 |
| ELANE | 3.19E-02 | ELANE | CTSB |
| ELANE | 6.79E-08 | CEBPA, ELANE, ERK1/2, estrogen receptor, HIF1A, IGF1R, IL1B, INSR, MAPK1, MAPK3, NFE2L2, NFkB (complex), NOS2, PRKAA1, RARA, RELA, STAT1, TP53 | ACTC1, ACTN4, CTSB, ECH1, GSTM1, HNRNPD, ICAM1, IDH3A, ITIH4, ITIH5, MAPK14, MSN, PAFAH1B2, PGD, PKM, PSME1, RPLP0, SERPINH1, SNX5, SORBS1, TGM2, TOP2B |
| ERK | 3.59E-02 | ERK | ICAM1, TGM2 |
| ESRRA | 4.27E-02 | ESRRA | LDHB, PKM |
| Esrra | 1.49E-02 | Esrra | ECH1, IDH3A |
| F3 | 3.19E-02 | F3 | ICAM1 |
| FAS | 1.87E-02 | FAS | COL15A1, ICAM1 |
| FGFR2 | 3.26E-06 | CEBPA, EGFR, ERBB3, ESR1, estrogen receptor, FAS, FGFR2, Mapk, MAPK14, MKNK1, MTOR, NFkB (complex), Ras, RB1, RICTOR, TP53, TSC2 | ACACA, ACTC1, ACTN4, COL15A1, ECH1, EEF1D, FDPS, HNRNPD, LDHB, MSN, PAFAH1B2, PGD, PSMC5, RPLP0, SERPINH1, SNX5, SORBS1, TOP2B, Tpm1 |
| FLT1 | 1.29E-02 | FLT1 | ICAM1 |
| FLT4 | 3.39E-07 | Akt, AMPK, FLT4, MMP9, NFkB (complex), NOS2, NOS3, RB1, RELA, SREBF2, STAT1, TSC2, USP8 | ACACA, ACTC1, CTSB, FDPS, HNRNPD, ICAM1, IDH3A, ITIH4, LDHB, PGD, PSME1, SERPINH1, TGM2, Tpm1 |
| FN1 | 8.70E-07 | Akt, CTNNB1, ERK1/2, estrogen receptor, FN1, HOXA10, IGF1R, ILK, INSR, IRS1, ITGAV, ITGB3, JAK2, Mapk, MAPK1, MKNK1, MTOR, MYC, NFkB (complex), PRKAA1, PTK2, SRC, STAT1, TCF7L2, TGFB1, TP53, TSC2 | ACACA, ACTC1, ACTN4, COL15A1, CTSB, ECH1, EEF1D, FBLN5, FBP2, FDPS, ICAM1, IDH3A, ITIH5, LDHB, MAPK14, MSN, NQO2, PAFAH1B2, PSME1, SNX5, SORBS1, TGM2, TOP2B |
| HBB | 1.71E-02 | HBB | ICAM1 |
| HGF | 3.63E-02 | HGF | ICAM1, MAPK14 |
| HIF1A | 1.89E-06 | CDKN1A, ERK1/2, HIF1A, JUN, MTORC1, RB1, TP53 | ACTC1, ACTN4, CTSB, ECH1, FDPS, ICAM1, PAFAH1B2, PGD, PKM, SERPINH1, SNX5, SORBS1, TOP2B, Tpm1 |
| HNF4A | 1.54E-06 | BRCA1, CEBPA, estrogen receptor, HIF1A, HMOX1, HNF4A, MAPK14, NFkB (complex), STAT1, TP53 | ACACA, ACTN4, CTSB, ECH1, FDPS, HNRNPD, ICAM1, MSN, PAFAH1B2, PGD, PKM, PSME1, SERPINH1, SNX5, SORBS1, TGM2, TOP2B |
| HRAS | 7.26E-08 | ERK, ERK1/2, FAS, HRAS, IRS1, Jnk, MAPK1, Mek, MTOR, MTORC1, NOS2, PGR, PRKAA1, RALA, RARA, RB1, STAT1, TCF7L2, TP53 | ACACA, ACTN4, COL15A1, CTSB, ECH1, FBP2, HNRNPD, ICAM1, ITIH4, ITIH5, LDHB, NQO2, PAFAH1B2, PGD, PKM, PSME1, SERPINH1, SNX5, SORBS1, TGM2, TOP2B, Tpm1 |
| Hsp90 | 3.90E-07 | AMPK, ERBB2, ERK, FAS, Focal adhesion kinase, GSK3B, HSF1, Hsp90, IGF1R, IP6K2, Jnk, MTOR, MYC, NFE2L2, NOS2, PRKAA2, PSEN1, RB1, SRC, STAT1, STK11, TP53 | ACTN4, COL15A1, ECH1, GNB1, GSTM1, ICAM1, ITIH4, ITIH5, LDHB, MAPK14, NQO2, PAFAH1B2, PGD, PSME1, RPLP0, SERPINH1, SNX5, SORBS1, TGM2, TOP2B |
| HSPB1 | 6.42E-07 | Akt, ERK1/2, FAS, HSF1, HSPB1, Jnk, MAP3K5, MAPK14, MTOR, NFE2L2, PRKAA1, PRKCD, PRKD1, PTEN, RB1, SRC, TP53, TSC2 | ACTN4, COL15A1, CTSB, ECH1, FDPS, GSTM1, IDH3A, ITIH5, LDHB, NQO2, PAFAH1B2, PGD, RPLP0, SERPINH1, SNX5, SORBS1, SUCLG2, TGM2, TOP2B, Tpm1 |
| HSPB6 | 6.46E-03 | HSPB6 | ICAM1 |
| IGF1R | 9.64E-06 | IGF1R, IRS1, MYC, PRKAA1, RB1, STAT1, TP53 | ACACA, ACTC1, ACTN4, COL15A1, ECH1, ITIH5, LDHB, MAPK14, PAFAH1B2, PGD, PKM, PSME1, SERPINH1, SNX5, SORBS1, TOP2B |
| IKBKB | 5.41E-09 | CEBPA, CTNNB1, ERK, estrogen receptor, FAS, IGF1R, IKBKB, INSR, IRS1, Jnk, MAPK8, MKNK1, MMP9, MTOR, MTORC1, MYC, NFE2L2, NFkB (complex), NOS2, P38 MAPK, STAT1, STAT3, TCF7L2, TGFB1, TP53 | ACACA, ACTC1, ACTN4, COL15A1, CTSB, ECH1, EEF1D, FBLN5, FBP2, FDPS, GSTM1, ICAM1, IDH3A, ITIH4, LDHB, MAPK14, MSN, PAFAH1B2, PGD, PSME1, RPLP0, SERPINH1, SNX5, SORBS1, TOP2B, Tpm1 |
| IKBKB | 1.58E-05 | CTNNB1, IKBKB, IRS1, MTOR, MTORC1, TP53 | ACACA, ACTC1, ACTN4, CTSB, ECH1, FDPS, ICAM1, LDHB, PAFAH1B2, SERPINH1, SNX5, SORBS1, TOP2B, Tpm1 |
| IKBKE | 3.27E-07 | ERK1/2, IKBKE, MTOR, RELA, STAT1, TP53 | ACTN4, CTSB, ECH1, FDPS, ICAM1, LDHB, PAFAH1B2, PKM, PSME1, SERPINH1, SNX5, SORBS1, TGM2, TOP2B, Tpm1 |
| IL1 | 2.38E-06 | AMPK, CEBPA, CTNNB1, estrogen receptor, HIF1A, IL1, JINK1/2, MAP3K7, Map3k7, MTOR, NFkB (complex), PRKAA1, PRKAA2, PRKCZ, TAB2, TP53, YES1 | ACTC1, ACTN4, ECH1, ICAM1, IDH3A, ITIH5, LDHB, MSN, NQO2, PAFAH1B2, PGD, PKM, SERPINH1, SNX5, SORBS1, TGM2, TOP2B |
| IL10 | 3.68E-10 | Akt, AMPK, CEBPA, estrogen receptor, FAS, IL10, Jnk, MKNK1, MMP9, MTORC1, NFE2L2, NFkB (complex), NOS2, P38 MAPK, PGR, RB1, TGFB1, TP53, USP8 | ACTC1, ACTN4, COL15A1, CTSB, ECH1, EEF1D, FBLN5, FDPS, GSTM1, HNRNPD, ICAM1, IDH3A, ITIH4, LDHB, MSN, NQO2, PAFAH1B2, PGD, PKM, RPLP0, SERPINH1, SNX5, SORBS1, TGM2, TOP2B |
| IL10 | 1.67E-06 | AMPK, IL10, Jnk, MKNK1, MMP9, MTORC1, NFkB (complex), NOS2 | ACTC1, CTSB, EEF1D, FDPS, ICAM1, IDH3A, ITIH4, LDHB, PKM, SERPINH1, TGM2 |
| IL13 | 1.77E-02 | IL13 | CTSB, LTA4H, TGM2 |
| IL18 | 3.11E-06 | AKT1, AMPK, BID, ERK, ERK1/2, ESR2, estrogen receptor, GATA4, IL18, NFATC4, NFE2L2, NFkB (complex), NOS2, PI3K (complex), PRKAA1, PRKCD, RICTOR, RPS6KB1, SRC, STAT1, TP53 | ACTC1, ACTN4, CTSB, ECH1, GSTM1, ICAM1, IDH3A, ITIH4, ITIH5, LDHB, MSN, NQO2, PAFAH1B2, PLEC, PSMC5, PSME1, SNX5, SORBS1, TGM2, TOP2B |
| IL-1R | 1.35E-07 | CEBPA, CTNNB1, ERK1/2, estrogen receptor, GSK3B, IGF1R, IL-1R, INSR, MAPK1, MAPK3, MAPK8, MKNK1, MYC, NFE2L2, NFkB (complex), P38 MAPK, PGR, PRKAA1, PSEN1, RARA, STAT1, TP53 | ACTC1, ACTN4, COL15A1, CTSB, ECH1, EEF1D, GNB1, GSTM1, HNRNPD, ICAM1, IDH3A, ITIH5, MAPK14, MSN, NQO2, PAFAH1B2, PGD, PSME1, RPLP0, SNX5, SORBS1, TGM2, TOP2B |
| IL1RN | 2.23E-07 | CEBPA, estrogen receptor, FAS, HIF1A, IFNG, IL1RN, MAPK14, NFkB (complex), NOS2, PKC alpha/beta, Pkc(s), PPARA, PRKD1, PTEN, SMPD1, SRC, TP53, YES1 | ACTC1, ACTN4, COL15A1, ECH1, FDPS, ICAM1, IDH3A, ITIH4, LDHB, MSN, NQO2, PAFAH1B2, PGD, PKM, PSME1, SNX5, SORBS1, SUCLG2, TGM2, TOP2B, Tpm1 |
| IL2 | 2.92E-07 | Akt, ERK1/2, estrogen receptor, FAS, FYN, IGF1R, IL2, IRS1, IRS2, JAK1, Jnk, Mapk, Mek, MKNK1, MMP9, MTOR, NFE2L2, NOS2, P38 MAPK, PRKAA1, PRKAA2, PTPN11, RB1, RICTOR, RPS6KB1, STAT5A, STAT6, TCF7L2, TGFB1, USP8 | ACACA, ACTC1, COL15A1, CTSB, EEF1D, FBLN5, FBP2, FDPS, GSTM1, HNRNPD, ITIH4, ITIH5, LDHB, MAPK14, MSN, NQO2, PGD, PKM, PSMC5, RPLP0, TGM2, Tpm1 |
| IL22 | 2.26E-06 | Akt, CASP1, estrogen receptor, IL22, MAPK14, RB1, SREBF2, STAT1, STAT3, STAT5A, STAT5a/b, TP53, TSC2, USP8 | ACACA, ACTC1, ACTN4, ECH1, FDPS, HNRNPD, ICAM1, LDHB, MSN, PAFAH1B2, PGD, PSME1, SERPINH1, SNX5, SORBS1, TOP2B, Tpm1 |
| IL25 | 4.41E-06 | CEBPA, estrogen receptor, FAS, HIF1A, IL25, Jnk, MKNK1, MTOR, MTORC1, NFE2L2, NFkB (complex), P38 MAPK, PGR, PPARG, RFWD2, STAT1, TP53 | ACACA, ACTN4, COL15A1, ECH1, EEF1D, GSTM1, ICAM1, LDHB, MSN, NQO2, PAFAH1B2, PGD, PKM, PSME1, RPLP0, SNX5, SORBS1, TGM2, TOP2B |
| INHBA | 1.88E-06 | Akt, CEBPA, ERK1/2, GSK3B, IGF1R, INHBA, IRS1, IRS2, MKNK1, MTOR, MYC, P38 MAPK, PGR, PPARG, PRKAA1, PSEN1, RB1, RFWD2, Smad2/3, SREBF2, TP53, TSC2, USP8 | ACACA, ACTC1, ACTN4, COL15A1, ECH1, EEF1D, GNB1, HNRNPD, ICAM1, ITIH5, LDHB, MAPK14, NQO2, PAFAH1B2, PGD, SNX5, SORBS1, TGM2, TOP2B, Tpm1 |
| Integrin | 2.11E-06 | Cdc42, Creb, CTNNB1, ERK1/2, FYN, HIF1A, IGF1R, INSR, Integrin, JUN, MKNK1, MTOR, MYC, NOS2, P38 MAPK, PI3K (complex), PRKAA1, PTK2, PTPN1, PTPRE, RHOA, SRC, STAT1, TCF7L2, TP53, TSC2 | ACTC1, ACTN4, COL15A1, CTSB, ECH1, EEF1D, FBP2, ICAM1, IDH3A, ITIH4, ITIH5, LDHB, MAPK14, NQO2, PAFAH1B2, PKM, PSME1, SNX5, SORBS1, TGM2, TOP2B |
| Interferon alpha | 2.94E-06 | CBL, CDK2, estrogen receptor, FAS, IGF1R, Interferon alpha, Jnk, MAPK1, MAPKAPK2, MKNK1, MTOR, NFE2L2, NFkB (complex), PGR, Pkc(s), RARA, RB1, SRC, STAT1, STAT3, TGFB1 | ACTC1, COL15A1, CTSB, EEF1D, FBLN5, FDPS, GSTM1, HNRNPD, ICAM1, LDHB, MAPK14, MSN, NQO2, PGD, PKM, PSME1, RPLP0, SERPINH1, TGM2, Tpm1 |
| Interferon alpha | 1.73E-05 | Interferon alpha, Jnk, MAPK1, MAPKAPK2, MKNK1, MTOR, NFkB (complex), Pkc(s), RB1, STAT1, STAT3 | ACTC1, CTSB, EEF1D, FDPS, ICAM1, LDHB, PGD, PKM, PSME1, SERPINH1, TGM2, Tpm1 |
| IRF2 | 6.17E-06 | ERK1/2, IRF2, STAT1, TP53 | ACTN4, CTSB, ECH1, ICAM1, PAFAH1B2, PSME1, SERPINH1, SNX5, SORBS1, TGM2, TOP2B, Tpm1 |
| ITGB3 | 3.84E-07 | AMPK, AR, CDK1, CTNNB1, ERK, ERK1/2, estrogen receptor, GSK3B, IGF1R, ITGB3, LCP2, MYC, NFkB (complex), NOS2, PRKAA1, PSEN1, Rac, SRC, STAT1, TCF7L2, TP53, VAV3 | ACTC1, ACTN4, COL15A1, CTSB, ECH1, FBP2, GNB1, GSTM1, ICAM1, IDH3A, ITIH4, ITIH5, MAPK14, MSN, NQO2, PAFAH1B2, PSME1, SERPINH1, SNX5, SORBS1, TGM2, TOP2B, Tpm1 |
| ITGB3 | 3.78E-06 | CTNNB1, ERK, ERK1/2, IGF1R, ITGB3, NFkB (complex), SRC, TP53 | ACTC1, ACTN4, CTSB, ECH1, ICAM1, MAPK14, NQO2, PAFAH1B2, SERPINH1, SNX5, SORBS1, TGM2, TOP2B, Tpm1 |
| KRAS | 9.72E-07 | Akt, AR, ATM, ERK, KRAS, Mek, MTOR, MTORC1, NOS2, PGR, RB1, SREBF2, STAT1, STK4, TCF7L2, TP53, TRIM28, TSC2, USP8 | ACACA, ACTN4, ALAD, ECH1, FBP2, GSTM1, HNRNPD, ICAM1, ITIH4, LDHB, NQO2, PAFAH1B2, PGD, PKM, PSME1, SERPINH1, SNX5, SORBS1, TOP2B, Tpm1 |
| LEP | 1.71E-06 | AHR, AMPK, Creb, CTNNB1, ESR1, ESR2, GSK3B, Jnk, LEP, Mapk, MAPK14, MKNK1, MTOR, NFE2L2, NOS2, PIK3R1, PRKAA2, PSEN1, PTEN, RICTOR, RPS6KB1 | ACACA, ACTC1, CTSB, ECH1, EEF1D, FBLN5, FBP2, GNB1, GSTM1, HNRNPD, ICAM1, ITIH4, ITIH5, LDHB, PKM, PLEC, PSMC5, RPLP0, SERPINH1, SUCLG2, Tpm1 |
| LEP | 1.30E-03 | LEP | ACACA, ECH1, FBP2, ICAM1 |
| let-7 | 1.27E-06 | CEBPA, ERK1/2, estrogen receptor, GATA1, IRS1, let-7, MMP9, MTOR, NFkB (complex), PRKAA1, TGFB1, TP53 | ACACA, ACTN4, CTSB, ECH1, FBLN5, FDPS, ICAM1, ITIH5, LDHB, MSN, PAFAH1B2, PGD, PKM, SNX5, SORBS1, TGM2, TOP2B, Tpm1 |
| LMNA | 3.81E-05 | LMNA, NFkB (complex), TP53 | ACTN4, CTSB, ECH1, ICAM1, PAFAH1B2, SERPINH1, SNX5, SORBS1, TGM2, TOP2B, Tpm1 |
| MAGI2 | 4.35E-06 | Akt, Cdc42, CTNNB1, estrogen receptor, HIF1A, IGF1R, MAGI2, MTOR, MTORC1, NFkB (complex), PI3K (family), PIK3R1, PTEN, RB1, RELA, SRC, STAT1, STAT6, USP8 | ACACA, ACTC1, CTSB, HNRNPD, IDH3A, LDHB, MAPK14, MSN, NQO2, PGD, PKM, PSME1, SUCLG2, TGM2, Tpm1 |
| MAP3K5 | 1.18E-06 | AMPK, CEBPA, CTNNB1, FAS, IGF1R, INSR, JINK1/2, Jnk, MAP3K5, MAPK8, MKNK1, NCF1, NFE2L2, NOS2, NOS3, P38 MAPK, TP53 | ACTC1, ACTN4, COL15A1, CTSB, ECH1, EEF1D, GSTM1, ICAM1, IDH3A, ITIH4, MAPK14, PAFAH1B2, PGD, RPLP0, SERPINH1, SNX5, SORBS1, TGM2, TOP2B, Tpm1 |
| MAP3K5 | 3.32E-05 | Jnk, MAP3K5, NCF1, NOS3, P38 MAPK, TP53 | ACTN4, CTSB, ECH1, ICAM1, PAFAH1B2, SERPINH1, SNX5, SORBS1, TGM2, TOP2B, Tpm1 |
| MAPK11 | 5.31E-04 | JUN, MAPK11, MTORC1, SRF | ACTC1, FDPS, ICAM1, PKM, Tpm1 |
| MAPK14 | 1.36E-03 | MAPK14 | FDPS, TGM2, Tpm1 |
| MLIP | 8.05E-07 | Akt, estrogen receptor, IGF1R, MLIP, PRKAA, PTEN, RB1, SRC, STAT1, TP53, USP8 | ACACA, ACTN4, ECH1, HNRNPD, IDH3A, LDHB, MAPK14, MSN, NQO2, PAFAH1B2, PGD, PSME1, SERPINH1, SNX5, SORBS1, SUCLG2, TOP2B, Tpm1 |
| MMP9 | 2.90E-03 | MMP9 | CTSB, ICAM1 |
| MTOR | 5.18E-03 | MTOR | FDPS, LDHB, PKM |
| MTOR | 4.06E-08 | Akt, AMPK, Gsk3, IGF1R, IRS1, MTOR, RB1, RHOA, SIRT1, STAT1, TFEB, TP53, USP8, XBP1 | ACACA, ACTN4, CTSB, ECH1, FDPS, HNRNPD, ICAM1, IDH3A, LDHB, MAPK14, PAFAH1B2, PGD, PKM, PSME1, SERPINH1, SNX5, SORBS1, TOP2B, Tpm1, TXNDC5 |
| MTORC1 | 2.17E-03 | MTORC1 | FDPS, PKM |
| MYD88 | 1.75E-06 | Akt, CASP1, CEBPA, ERK1/2, estrogen receptor, FAS, INSR, IRAK1, IRS1, Jnk, Mapk, MAPK8, MKNK1, MTOR, MYD88, NFkB (complex), NOS2, P38 MAPK, PGR, PI3K (complex), PPARG, PRKAA1, RB1, RFWD2, SREBF2, TP53, USP8 | ACACA, ACTC1, ACTN4, COL15A1, ECH1, EEF1D, FDPS, HNRNPD, ICAM1, IDH3A, ITIH4, ITIH5, LDHB, MSN, NQO2, PAFAH1B2, PKM, SNX5, SORBS1, TGM2, TOP2B |
| NCF1 | 2.56E-02 | NCF1 | ICAM1 |
| NCF1 | 1.82E-07 | ATM, CEBPA, ERK, estrogen receptor, FYN, HIF1A, HOXA10, JAK2, JUN, MAP3K5, MTOR, NCF1, NFkB (complex), NOS2, TP53, TRIM28 | ACACA, ACTC1, ACTN4, ALAD, COL15A1, ECH1, FDPS, ICAM1, ITIH4, LDHB, MSN, NQO2, PAFAH1B2, PGD, PKM, SNX5, SORBS1, TGM2, TOP2B, Tpm1 |
| NFE2L2 | 3.52E-02 | NFE2L2 | GSTM1, RPLP0, Tpm1 |
| NFkB (complex) | 8.05E-06 | CEBPA, estrogen receptor, HIF1A, NFkB (complex), TP53 | ACACA, ACTN4, ECH1, ICAM1, MSN, PAFAH1B2, PGD, PKM, SERPINH1, SNX5, SORBS1, TGM2, TOP2B, Tpm1 |
| NOS2 | 5.92E-03 | NOS2 | ACTC1, ITIH4, SERPINH1 |
| NOS2 | 1.81E-08 | Akt, AR, estrogen receptor, FOXO1, GSK3B, IGF1R, IRS1, IRS2, Jnk, MMP9, MTOR, NFE2L2, NFkB (complex), NOS2, PSEN1, Rap1, SRC, TGFB1, TP53, USP8 | ACACA, ACTC1, ACTN4, ECH1, FBLN5, FDPS, GNB1, GSTM1, HNRNPD, ICAM1, ITIH4, LDHB, MAPK14, MSN, NQO2, PAFAH1B2, PKM, RPLP0, SERPINH1, SNX5, SORBS1, TGM2, TOP2B |
| NOS2 | 5.70E-05 | IRS1, IRS2, Jnk, MMP9, MTOR, NFkB (complex), NOS2 | ACACA, ACTC1, FDPS, ICAM1, ITIH4, LDHB, PKM, SERPINH1, TGM2 |
| NOS3 | 3.14E-09 | AMPK, estrogen receptor, MAP2K1/2, MAPK3, MKNK1, MMP9, MTOR, MTORC1, NFE2L2, NFkB (complex), NOS2, NOS3, PRKAA1, RARA, STAT1, TGFB1, TP53 | ACTC1, ACTN4, CTSB, ECH1, EEF1D, FBLN5, FDPS, GSTM1, HNRNPD, ICAM1, IDH3A, ITIH4, ITIH5, LDHB, MSN, PAFAH1B2, PSME1, RPLP0, SERPINH1, SNX5, SORBS1, TGM2, TOP2B |
| NOS3 | 8.94E-06 | AMPK, MMP9, NFkB (complex), NOS2, NOS3, STAT1 | ACTC1, CTSB, ICAM1, IDH3A, ITIH4, LDHB, PSME1, SERPINH1, TGM2 |
| NR3C2 | 1.43E-05 | Creb, CTNNB1, NR3C2, PPARG, RELA, TP53 | ACACA, ACTC1, ACTN4, CTSB, ECH1, FDPS, ICAM1, PAFAH1B2, SERPINH1, SNX5, SORBS1, TGM2, TOP2B, Tpm1 |
| PDCD1 | 7.41E-07 | CEBPA, estrogen receptor, HIF1A, IGF1R, NFkB (complex), PDCD1, PRKAA2, PTPN11, STAT1, TP53, TSC2 | ACTN4, ECH1, ICAM1, ITIH5, LDHB, MAPK14, MSN, PAFAH1B2, PGD, PKM, PSME1, SERPINH1, SNX5, SORBS1, TGM2, TOP2B, Tpm1 |
| Pdgf (complex) | 2.48E-06 | AKT1, AMPK, HOXA10, JAK2, KRAS, MTOR, NFKBIA, NOS2, Pdgf (complex), PI3K (complex), RB1, TP53 | ACTC1, ACTN4, COL15A1, ECH1, FDPS, IDH3A, ITIH4, PAFAH1B2, PGD, PKM, SERPINH1, SNX5, SORBS1, TGM2, TOP2B, Tpm1 |
| PECAM1 | 2.72E-06 | CEBPA, CTNNB1, estrogen receptor, FAS, FCER1G, FYN, HIF1A, Integrin, IRF3, Jnk, MTORC1, NFE2L2, NFkB (complex), PECAM1, SRC, STAT1, STAT3, TP53 | ACTC1, ACTN4, COL15A1, ECH1, FDPS, GSTM1, MSN, NQO2, PAFAH1B2, PGD, PKM, PSME1, RPLP0, SERPINH1, SNX5, SORBS1, TGM2, TOP2B |
| PLAUR | 3.40E-02 | PLAUR | CTSB |
| PPARA | 8.36E-03 | PPARA | ACACA, ECH1, FDPS, POR |
| PPARD | 4.58E-02 | PPARD | ECH1, LDHB |
| PPARG | 8.36E-03 | PPARG | ACACA, FDPS, ICAM1, SORBS1 |
| PPP1CB | 7.82E-06 | AKT1, AMPK, CEBPA, estrogen receptor, HIF1A, N-cor, NFkB (complex), PKC alpha/beta, PPP1CB, SRC, TP53 | ACACA, ACTN4, ECH1, ICAM1, IDH3A, MSN, NQO2, PAFAH1B2, PGD, PKM, SERPINH1, SNX5, SORBS1, TGM2, TOP2B, Tpm1 |
| PRKAA1 | 1.30E-02 | PRKAA1 | ITIH5, TGM2 |
| PRKAA2 | 2.09E-02 | PRKAA2 | ITIH5, TGM2 |
| PSEN1 | 1.09E-03 | PSEN1 | GNB1, PAFAH1B2, PKM, TOP2B, Tpm1 |
| PSEN1 | 1.54E-06 | Akt, CTNNB1, ERK1/2, GSK3B, INSR, MAPK8, MYC, PRKAA1, PSEN1, RB1, SRC, STAT1, TP53, USP8 | ACTC1, ACTN4, COL15A1, CTSB, ECH1, GNB1, HNRNPD, ICAM1, IDH3A, ITIH5, NQO2, PAFAH1B2, PGD, PSME1, SERPINH1, SNX5, SORBS1, TGM2, TOP2B, Tpm1 |
| PSEN1 | 1.88E-06 | ERK1/2, PSEN1, SRC, TP53 | ACTN4, CTSB, ECH1, GNB1, ICAM1, NQO2, PAFAH1B2, SERPINH1, SNX5, SORBS1, TGM2, TOP2B, Tpm1 |
| PSEN2 | 5.86E-06 | ERK1/2, PSEN2, TP53 | ACTN4, CTSB, ECH1, GNB1, ICAM1, PAFAH1B2, SERPINH1, SNX5, SORBS1, TGM2, TOP2B, Tpm1 |
| PTEN | 8.11E-06 | PTEN | ACACA, CTSB, IDH3A, LDHB, MAPK14, SUCLG2 |
| PTEN | 5.19E-04 | PTEN | ACACA, IDH3A, LDHB, SUCLG2 |
| PTGER1 | 1.08E-02 | PTGER1 | ICAM1 |
| PTGS2 | 3.31E-02 | PTGS2 | ICAM1, TGM2 |
| RAPGEF3 | 1.73E-08 | AMPK, Cdc42, ERN1, MTORC1, PIK3CG, RAPGEF3, RB1, RHOA, TP53 | ACACA, ACTC1, ACTN4, CTSB, ECH1, FDPS, ICAM1, IDH3A, LDHB, PAFAH1B2, PGD, PKM, SERPINH1, SNX5, SORBS1, TOP2B, Tpm1 |
| RAPGEF3 | 5.19E-04 | AMPK, Cdc42, PIK3CG, RAPGEF3 | CTSB, ICAM1, IDH3A, LDHB |
| RELA | 1.28E-02 | RELA | ACTN4, ICAM1, TGM2 |
| REN | 4.44E-02 | REN | ICAM1 |
| REN | 2.89E-06 | CEBPA, ERK, estrogen receptor, HIF1A, MTOR, NFkB (complex), NOS2, REN, TP53 | ACACA, ACTC1, ACTN4, ECH1, FDPS, ICAM1, ITIH4, LDHB, MSN, PAFAH1B2, PGD, PKM, SNX5, SORBS1, TGM2, TOP2B, Tpm1 |
| RHOA | 4.64E-07 | CEBPA, CTNNB1, Erm, FAS, IGF1R, Integrin, IRS1, Jnk, MAPK9, MKNK1, MTOR, MTORC1, NFE2L2, P38 MAPK, PIK3R1, Pld, PPARG, RFWD2, RHOA, ROCK1, STAT1, TP53, TSC2 | ACACA, ACTC1, ACTN4, COL15A1, CTSB, ECH1, EEF1D, GSTM1, ICAM1, LDHB, MAPK14, PAFAH1B2, PGD, PKM, PSME1, RPLP0, SERPINH1, SNX5, SORBS1, TOP2B, Tpm1 |
| RNF31 | 2.93E-08 | AHR, AMPK, CEBPA, ESR1, ESR2, estrogen receptor, FAS, IGF1R, IKBKG, Jnk, MAP3K7, MTORC1, NFE2L2, NFkB (complex), RNF31, TP53 | ACTN4, COL15A1, CTSB, ECH1, FBLN5, FDPS, GSTM1, HNRNPD, IDH3A, LDHB, MAPK14, MSN, PAFAH1B2, PGD, PLEC, RPLP0, SERPINH1, SNX5, SORBS1, TOP2B, Tpm1 |
| RUNX1 | 2.59E-06 | CD4, CEBPA, estrogen receptor, FYN, HIF1A, NFkB (complex), RUNX1, TP53 | ACACA, ACTN4, ECH1, ICAM1, MSN, NQO2, PAFAH1B2, PGD, PKM, SERPINH1, SNX5, SORBS1, TGM2, TOP2B, Tpm1 |
| S1PR2 | 2.77E-02 | S1PR2 | ICAM1 |
| SCD | 1.18E-05 | AMPK, INSR, NOS2, P38 MAPK, SCD, STAT1 | ACACA, ACTC1, FDPS, ICAM1, IDH3A, ITIH4, LDHB, PSME1, SERPINH1 |
| SELE | 1.96E-07 | AR, CEBPA, ERK, Hsp27, MKNK1, MTOR, NOS2, P38 MAPK, PGR, PPARG, RFWD2, SELE, STAT1, TP53 | ACACA, ACTC1, ACTN4, ECH1, EEF1D, GSTM1, ICAM1, ITIH4, LDHB, NQO2, PAFAH1B2, PGD, PKM, PSME1, SERPINH1, SNX5, SORBS1, TGM2, TOP2B, Tpm1 |
| SEMA7A | 4.65E-02 | SEMA7A | CTSB |
| SERPINH1 | 1.30E-05 | Akt, MTOR, NFKBIA, PI3K (family), RB1, SERPINH1, SREBF1, SREBF2, STAT1, TP53, TSC2, USP8 | ACACA, ACTC1, ACTN4, ECH1, HNRNPD, ICAM1, LDHB, PAFAH1B2, PGD, PKM, PSME1, SNX5, SORBS1, TOP2B, Tpm1 |
| Smad2/3 | 3.82E-02 | Smad2/3 | TGM2 |
| SMAD4 | 3.73E-02 | SMAD4 | ICAM1, POR |
| SMPD1 | 2.35E-02 | SMPD1 | CTSB |
| SOCS3 | 7.74E-06 | Akt, AKT1, AMPK, CFTR, ERK1/2, HOXA10, IRS1, JAK1, JAK2, LEPR, MKNK1, MTOR, NFKBIA, PIK3R1, Pka, PRKAA1, RB1, RHO, SOCS3, STAT1, TP53, USP8 | ACTC1, ACTN4, COL15A1, ECH1, EEF1D, GNB1, HNRNPD, IDH3A, ITIH5, PAFAH1B2, PGD, PKM, POR, PSME1, SERPINH1, SNX5, SORBS1, TOP2B |
| SOD2 | 3.31E-06 | BID, CEBPA, estrogen receptor, FAS, HIF1A, Jnk, MTORC1, NFE2L2, NFkB (complex), SOD2, TP53 | ACACA, ACTN4, COL15A1, CTSB, ECH1, GSTM1, ICAM1, MSN, PAFAH1B2, PGD, PKM, RPLP0, SERPINH1, SNX5, SORBS1, TGM2, TOP2B |
| SPP1 | 2.01E-02 | SPP1 | PGD, POR |
| SRC | 5.04E-03 | SRC | ICAM1, NQO2 |
| SREBF1 | 3.80E-03 | ACACA, FDPS, RPLP0 | SREBF1, SREBF2 |
| SYVN1 | 3.73E-05 | HTT, SYVN1, TP53 | ACTN4, CTSB, ECH1, LDHB, PAFAH1B2, SERPINH1, SNX5, SORBS1, TGM2, TOP2B, Tpm1 |
| TBXA2R | 6.46E-03 | TBXA2R | ICAM1 |
| TERC | 2.73E-06 | IGF1R, MAPK9, MTOR, PGR, PIK3R1, RB1, TERC, TERT, TP53 | ACACA, ACTC1, ACTN4, CTSB, ECH1, LDHB, MAPK14, NQO2, PAFAH1B2, PGD, PKM, SERPINH1, SNX5, SORBS1, TGM2, TOP2B, Tpm1 |
| TGFB1 | 3.30E-02 | TGFB1 | FBLN5, ICAM1, SERPINH1, TGM2 |
| TGFB1 | 1.72E-06 | ATM, CTNNB1, estrogen receptor, HIF1A, IRS1, MAP2K1/2, MAPK13, MAPK14, MKNK1, NFkB (complex), P38 MAPK, PPARG, PRKAA1, PTEN, RICTOR, RPS6KB1, SRC, TCF7L2, TGFB1, TGM2, TP53, TRIM28 | ACACA, ACTC1, ACTN4, ALAD, ECH1, EEF1D, FBLN5, FBP2, FDPS, ICAM1, ITIH5, MSN, NQO2, PAFAH1B2, PKM, POR, PSMC5, SERPINH1, SNX5, SORBS1, SUCLG2, TGM2, TOP2B, Tpm1 |
| TGFB1 | 1.72E-06 | CTNNB1, HIF1A, IRS1, MAPK14, PPARG, PRKAA1, SRC, TGFB1, TGM2, TP53 | ACACA, ACTC1, ACTN4, ECH1, FBLN5, FDPS, ICAM1, ITIH5, NQO2, PAFAH1B2, PKM, POR, SERPINH1, SNX5, SORBS1, TGM2, TOP2B, Tpm1 |
| THBS1 | 1.54E-07 | CTNNB1, HIF1A, IRS1, MAPK14, MAPK9, PPARG, PRKAA1, RB1, Smad2/3, SRC, TGFB1, TGM2, THBS1, TP53 | ACACA, ACTC1, ACTN4, ECH1, FBLN5, FDPS, ICAM1, ITIH5, NQO2, PAFAH1B2, PGD, PKM, POR, SERPINH1, SNX5, SORBS1, TGM2, TOP2B, Tpm1 |
| THBS4 | 4.65E-02 | THBS4 | ICAM1 |
| THBS4 | 6.31E-03 | ATF6, THBS4 | FDPS, ICAM1 |
| TICAM1 | 6.85E-07 | CASP1, CCL5, ERK, ERK1/2, estrogen receptor, FAS, IGF1R, IL1B, IL6, Jnk, MAPK14, MYC, NFE2L2, NFkB (complex), NOS2, P38 MAPK, PGR, PRKAA1, RELA, SRC, STAT1, TICAM1, TNF, TP53 | ACACA, ACTN4, COL15A1, CTSB, ECH1, FDPS, GSTM1, ICAM1, ITIH4, ITIH5, MAPK14, MSN, NQO2, PAFAH1B2, PKM, PLEC, PSME1, RPLP0, SERPINH1, SNX5, TOP2B, Tpm1 |
| TNF | 3.24E-02 | TNF | CTSB, ICAM1, MAPK14, PKM, TGM2 |
| TNFSF14 | 5.30E-06 | CEBPA, estrogen receptor, HIF1A, INSR, MAPK8, NFkB (complex), TNFSF14, TP53 | ACACA, ACTN4, ECH1, FDPS, ICAM1, IDH3A, MSN, PAFAH1B2, PGD, PKM, SERPINH1, SNX5, SORBS1, TGM2, TOP2B, Tpm1 |
| TNFSF14 | 2.77E-02 | TNFSF14 | ICAM1 |
| TP53 | 2.49E-05 | TP53 | ACTN4, CTSB, ECH1, FDPS, PAFAH1B2, SERPINH1, SNX5, SORBS1, TGM2, TOP2B, Tpm1 |
| TP53 | 1.02E-08 | Akt, AR, BBC3, CDK1, CYP19A1, ERK, estrogen receptor, GSK3B, IGF1R, Jnk, MAPK9, Mek, MKNK1, MTOR, NFE2L2, NFkB (complex), NOS2, PGR, PI3K (complex), PIK3R1, PRKAA1, PRKAA2, PSEN1, PTEN, TCF7L2, TP53, USP8, XBP1 | ACACA, ACTN4, CTSB, ECH1, EEF1D, FBP2, GNB1, GSTM1, HNRNPD, ITIH4, ITIH5, LDHB, MAPK14, MSN, NQO2, PAFAH1B2, PKM, RPLP0, SERPINH1, SNX5, SORBS1, SUCLG2, TGM2, TOP2B, Tpm1, TXNDC5 |
| TP53 | 4.04E-05 | TP53 | ACTN4, CTSB, ECH1, PAFAH1B2, SERPINH1, SNX5, SORBS1, TGM2, TOP2B, Tpm1 |
| TPSD1 | 6.12E-07 | HIF1A, HSF1, INSR, MAPK1, MAPK14, MAPK3, MAPK8, MAPK9, MTOR, NFE2L2, RARA, RB1, TP53, TPSD1 | ACACA, ACTN4, CTSB, ECH1, FDPS, GSTM1, HNRNPD, IDH3A, LDHB, PAFAH1B2, PGD, PKM, RPLP0, SERPINH1, SNX5, SORBS1, TGM2, TOP2B, Tpm1 |
| TRAF3IP2 | 2.90E-06 | CEBPA, estrogen receptor, HIF1A, IRF3, MAPK7, NFkB (complex), PPARG, STAT1, TP53, TRAF3IP2 | ACACA, ACTN4, ECH1, FDPS, ICAM1, MSN, PAFAH1B2, PGD, PKM, PSME1, SERPINH1, SNX5, SORBS1, TGM2, TOP2B, Tpm1 |
| VEGFA | 1.42E-02 | VEGFA | CTSB, ICAM1 |
| **obob** |  |  |  |
| **Master Regulator** | **p-value of overlap** | **Participating regulators** | **Target molecules in dataset** |
| ADIPOQ | 1.05E-06 | ADIPOQ, Akt, AMPK, CTNNB1, EIF4E, HIF1A, IGF1R, Jnk, KRAS, MKNK1, MTOR, NFE2L2, NFkB (complex), P38 MAPK, PADI2, PI3K (complex), RB1, RELA, RHOA, STK11, TCF7L2, TP53 | ACACA, ALDH9A1, Ces1b/Ces1c, ERAP1, FAH, FBP1, GBE1, GPC4, GPS1, IDH3A, MAPK14, MDH1, NAE1, NME1, PARK7, PDHB, PRDX4, RAB6B, THOP1 |
| ADORA2A | 1.12E-05 | ADCY, ADORA2A, AMPK, CASP1, EIF4E, HIF1A, Mapk, MAPK1, MAPK3, MKNK1, NFE2L2, NFkB (complex), NTRK1, P38 MAPK, PADI2, RB1, SOD2, TP53 | ACACA, ALDH9A1, Ces1b/Ces1c, ERAP1, GBE1, GPC4, GPS1, IDH3A, LAMA4, NAE1, NME1, PARK7, PDHB, RAB6B, TGFBI, THOP1, YWHAZ |
| ADORA2B | 5.66E-06 | ADORA2B, Akt, CEBPA, IGF1R, MAPK1, MKNK1, NFkB (complex), P38 MAPK, PADI2, PTEN, RB1, SPHK1, TP53 | ACACA, ALDH9A1, Ces1b/Ces1c, CYCS, ERAP1, GPC4, IDH3A, LAMA4, MAPK14, MDH1, NME1, PARK7, PDHB, RAB6B, TGFBI, THOP1 |
| AGT | 4.35E-06 | AGT, BAX, Cdc42, CEBPA, HIF1A, IKBKB, INSR, JUN, MAPK8, MYD88, NFkB (complex), SYK, TICAM1, TP53 | ACACA, AK3, ALDH9A1, Ces1b/Ces1c, CTSS, CYCS, ERAP1, GPT, IDH3A, LMNB1, NAE1, NME1, PDHB, TGFBI, THOP1 |
| AGTR1 | 7.30E-07 | AGTR1, Akt, BAD, BAX, BID, CD38, CEBPA, EIF4E, ERK1/2, HIF1A, IKBKB, IRS1, Jnk, Mapk, MKNK1, NFE2L2, NFkB (complex), NR3C2, PLCB1, RB1, RELA, SREBF2, TP53 | ACACA, AK3, ALDH9A1, Ces1b/Ces1c, CTSS, CYCS, ERAP1, GBE1, GPC4, GPS1, LMNB1, MDH1, NAE1, PARK7, PDHB, PRDX4, TGFBI, THOP1 |
| AGTR2 | 4.44E-05 | AGTR2, CDK2, IGF1R, IKBKB, INSR, KRAS, MAPK1, MAPK3, NFE2L2, PI3K (complex), PIK3R1, RHOA, SMARCA4, Tgf beta, TP53 | ALDH9A1, Ces1b/Ces1c, CYCS, ERAP1, FAH, FBP1, GBE1, IDH3A, LAMA4, LMNB1, MAPK14, PDHB, PRDX4, TGFBI, THOP1 |
| ATF3 | 1.92E-05 | ATF3, BAD, BAX, CEBPA, EIF4E, ERK, IKBKB, Jnk, JUN, MKNK1, NFE2L2, NOS2, P38 MAPK, PADI2, RB1, RXRA, TP53 | ALDH9A1, Ces1b/Ces1c, CTSS, CYCS, FBP1, GBE1, GPC4, GPS1, GPT, LMNB1, MDH1, NME1, PARK7, RAB6B, TGFBI, THOP1 |
| BAX | 1.60E-04 | BAX, CASP1, CASP8, caspase, HIF1A, IKBKB, Jnk, JUN, MAP3K5, NFE2L2, NFkB (complex), RELA, SREBF1, TICAM1, TP53, TRAF2 | ACACA, AK3, ALDH9A1, Ces1b/Ces1c, CTSS, ERAP1, GBE1, GPT, LMNB1, NAE1, NME1, TGFBI, THOP1 |
| BCL2 | 7.12E-05 | BAX, BCL2, BID, CEBPA, HIF1A, IKBKB, MAPK1, NFkB (complex), RB1, SPHK1, TERT, TP53 | ALDH9A1, Ces1b/Ces1c, CTSS, CYCS, ERAP1, GPT, LAMA4, LMNB1, MDH1, NAE1, PARK7, TGFBI, THOP1 |
| BCL6 | 7.35E-06 | BAX, BCL6, CDKN2A, E2F1, EIF4E, HIF1A, IGF1R, IKBKB, Jnk, KRAS, MKNK1, NFE2L2, NFkB (complex), P38 MAPK, PADI2, SOD2, TP53 | ALDH9A1, Ces1b/Ces1c, CTSS, ERAP1, FAH, GBE1, GPC4, GPS1, IDH3A, LMNB1, MAPK14, NAE1, NME1, PRDX4, RAB6B, TGFBI, THOP1, YWHAZ |
| BID | 2.46E-04 | BID, CASP1, CASP8, caspase, CHEK1, E2F3, NFkB (complex), RELA, TP53 | ACACA, ALDH9A1, Ces1b/Ces1c, CTSS, ERAP1, GPT, LAMA4, NME1, TGFBI, THOP1 |
| CAPNS1 | 6.24E-05 | Akt, BAX, BID, CAPNS1, CASP3, CEBPA, HIF1A, IKBKB, Jnk, JUN, NFE2L2, NFkB (complex), RB1, SREBF2, TP53 | ACACA, ALDH9A1, Ces1b/Ces1c, CTSS, CYCS, ERAP1, GBE1, GPT, LMNB1, MDH1, NAE1, PARK7, TGFBI, THOP1 |
| CASP1 | 6.47E-06 | BAX, BID, CASP1, CASP3, CASP7, CEBPA, EIF4E, HIF1A, IKBKB, JUN, MKNK1, MTOR, NFkB (complex), P38 MAPK, PADI2, PPARG, TP53 | ACACA, ALDH9A1, Ces1b/Ces1c, CTSS, CYCS, ERAP1, GPC4, GPS1, GPT, LMNB1, MDH1, NAE1, NME1, RAB6B, TGFBI, THOP1 |
| CAV1 | 4.05E-06 | AMPK, c-Src, CAV1, Cdc42, EIF4E, GSK3B, HIF1A, IGF1R, IKBKB, IRS1, Jnk, MAPK1, MAPK3, Mek, NFE2L2, RB1, SOD2, TCF7L2, TP53 | ACACA, ALDH9A1, Ces1b/Ces1c, CTSS, FBP1, GBE1, GPS1, IDH3A, LAMA4, LMNB1, MAPK14, MDH1, NAE1, PARK7, PDHB, TGFBI, THOP1, YWHAZ |
| CAV1 | 2.81E-05 | AMPK, CAV1, Cdc42, IKBKB, IRS1, MAPK1, RB1, TP53 | ACACA, ALDH9A1, Ces1b/Ces1c, CTSS, IDH3A, LAMA4, LMNB1, MDH1, PARK7, PDHB, TGFBI, THOP1 |
| CBL | 3.18E-05 | AKT1, AMPK, BTK, CBL, EIF4E, ERK, HIF1A, IGF1R, IKBKB, KRAS, MAPK1, MAPK3, MTOR, NFE2L2, PDPK1, PI3K (complex), PRKAA, TGFB1, TP53 | ALDH9A1, Ces1b/Ces1c, CYCS, ERAP1, FAH, GBE1, GPS1, IDH3A, LAMA4, LMNB1, MAPK14, MDH1, NAE1, PDHB, PRDX4, TGFBI, THOP1 |
| Ccl2 | 3.42E-04 | BAX, Ccl2, CEBPA, EIF4E, ERK, ERK1/2, HIF1A, IKBKB, IRS1, Jnk, JUN, MKNK1, MTOR, NFE2L2, NFkB (complex), NOS2, NR3C2, P38 MAPK, PADI2, PPARG, RELA, RFWD2, RXRA | ACACA, CTSS, CYCS, ERAP1, GBE1, GPC4, GPS1, GPT, LMNB1, MDH1, NAE1, PDHB, RAB6B |
| CCR1 | 3.74E-02 | CCR1 | CTSS |
| CCR5 | 2.45E-05 | BID, CASP1, CASP8, CCR5, CEBPA, CXCR3, HIF1A, IKBKB, MAPK1, NFkB (complex), TP53 | ACACA, ALDH9A1, Ces1b/Ces1c, CTSS, CYCS, ERAP1, GPT, LAMA4, LMNB1, NAE1, NME1, TGFBI, THOP1 |
| CEBPB | 2.39E-07 | ACLY, ATM, BAX, CASP1, CDK2, CEBPB, EIF4E, HIF1A, IGF1R, IKBKB, INSR, JUN, KRAS, NFkB (complex), PI3K (complex), PRKCE, SMARCA4, SOD2, TP53 | ACACA, ALDH9A1, Ces1b/Ces1c, CTSS, CYCS, ERAP1, FAH, FBP1, GPS1, GPT, IDH3A, LMNB1, MAPK14, NAE1, NME1, PDHB, PRDX4, TGFBI, THOP1, YWHAZ |
| CEBPB | 3.54E-04 | ACLY, CASP1, CEBPB, INSR, NFkB (complex), TP53 | ACACA, ALDH9A1, Ces1b/Ces1c, CYCS, ERAP1, IDH3A, NME1, PDHB, TGFBI, THOP1 |
| CNR1 | 1.71E-03 | AMPK, CNR1, JUN, MAPK1 | ACACA, ERAP1, GPT, IDH3A, LAMA4, PDHB |
| COL4A3 | 4.09E-02 | COL4A3 | MAPK14 |
| CXCL10 | 6.97E-06 | AKT1, AMPK, BAX, CBX7, CXCL10, ERK1/2, IKBKB, KRAS, MAPK1, Mek, NOS2, NR3C2, PI3K (complex), RAF1, RARA, RB1, Rsk, TCF7L2, TP53 | ALDH9A1, Ces1b/Ces1c, CTSS, CYCS, ERAP1, FAH, FBP1, IDH3A, LAMA4, LMNB1, MDH1, NME1, PARK7, PDHB, PRDX4, TGFBI, THOP1 |
| CXCL12 | 8.55E-06 | Akt, Cdc42, CEBPA, CHUK, CXCL12, EIF4E, ERBB2, ERK, ERK1/2, FOXO3, HIF1A, IRS1, JUN, KRAS, MAPK1, Mek, MKNK1, MYD88, NOS2, NR3C2, P38 MAPK, PADI2, PI3K (complex), PI3K (family), PPARG, RFWD2, RHOA, ROCK1, SREBF1, SREBF2, SYK, TCF7L2, Tgf beta, TICAM1, TP53 | ACACA, AK3, ALDH9A1, Ces1b/Ces1c, CTSS, CYCS, ERAP1, FAH, FBP1, GPC4, GPS1, GPT, LAMA4, NAE1, PDHB, PRDX4, RAB6B, TGFBI, THOP1 |
| CYP2E1 | 1.13E-05 | BAX, CEBPA, CYP2E1, EIF4E, ERK1/2, HIF1A, IKBKB, IRS1, MKNK1, MTOR, NFkB (complex), NR3C2, P38 MAPK, PADI2, PPARG, RFWD2, TP53 | ACACA, ALDH9A1, Ces1b/Ces1c, CTSS, CYCS, ERAP1, GPC4, GPS1, LMNB1, MDH1, NAE1, NME1, PDHB, RAB6B, TGFBI, THOP1 |
| DYSF | 8.84E-03 | DYSF | CTSS, IDH3A |
| Esrra | 2.47E-03 | AMPK, Esrra, RELA | ERAP1, FBP1, IDH3A, PDHB |
| F2RL1 | 4.14E-04 | CEBPA, ERK1/2, F2RL1, HIF1A, IKBKB, IRS1, JUN, NFkB (complex), NR3C2, TP53 | ACACA, ALDH9A1, Ces1b/Ces1c, CYCS, ERAP1, GPT, LMNB1, NAE1, NME1, PDHB, TGFBI, THOP1 |
| FASLG | 5.40E-06 | BAX, BID, CASP1, CASP3, CASP8, CEBPA, EIF4E, ERK1/2, FASLG, FYN, HIF1A, IKBKB, INSR, IRS1, JUN, MAPK1, MAPK8, MAPK9, MKNK1, NCF1, NFkB (complex), NOS2, NR3C2, P38 MAPK, PADI2, PPARG, PTK2B, RB1, RFWD2, SRC, TP53 | ACACA, ALDH9A1, Ces1b/Ces1c, CTSS, CYCS, ERAP1, GPC4, GPS1, GPT, IDH3A, LAMA4, LMNB1, NAE1, PARK7, PDHB, RAB6B, TGFBI, THOP1 |
| FCGR2A | 1.04E-08 | Akt, AMPK, BAX, CASP1, Cdc42, CEBPA, ERK, FCGR2A, HIF1A, IGF1R, IKBKB, INPP5D, Integrin, JUN, LCP2, MAPK1, MKNK1, MTOR, MYC, MYD88, NFkB (complex), NOS2, PADI2, Pkc(s), PTPN1, Rac, RB1, RXRA, SYK, TICAM1, TP53 | ACACA, AK3, ALDH9A1, Ces1b/Ces1c, CTSS, CYCS, ERAP1, GPC4, GPT, IDH3A, LAMA4, LMNB1, MAPK14, MDH1, NAE1, NME1, PARK7, PDHB, RAB6B, TGFBI, THOP1 |
| GDNF | 6.16E-05 | Akt, ERK1/2, ESR1, GDNF, IKBKB, IRS1, Jnk, JUN, KRAS, MAPK1, NFE2L2, NOS2, NR3C2, PI3K (complex), RARA, Ras, RB1, SREBF2 | ACACA, CTSS, CYCS, ERAP1, FAH, GBE1, GPT, LAMA4, LMNB1, MDH1, NME1, PARK7, PDHB, PRDX4 |
| Growth hormone | 4.50E-05 | Akt, BAX, BID, CASP3, EIF4E, Growth hormone, IKBKB, IRS1, KRAS, Mapk, MAPK1, MAPK3, MKNK1, NFE2L2, NOS2, PI3K (complex), RARA, RB1, SREBF2 | ACACA, CTSS, CYCS, ERAP1, FAH, GBE1, GPC4, GPS1, LAMA4, LMNB1, MDH1, NME1, PARK7, PRDX4 |
| HCK | 3.99E-06 | Akt, AMPK, BTK, CEBPA, EIF4E, ERK, HCK, HIF1A, IKBKB, INPP5D, INSR, JUN, KRAS, LAT, MAPK1, MAPK3, MAPK8, MKNK1, MYD88, NFE2L2, NFkB (complex), NOS2, P38 MAPK, PADI2, PI3K (complex), RB1, RXRA, SYK, TICAM1, TP53, TP73 | AK3, ALDH9A1, Ces1b/Ces1c, CYCS, FAH, GBE1, GPC4, GPS1, GPT, IDH3A, LAMA4, LMNB1, NAE1, PARK7, PDHB, PRDX4, RAB6B, TGFBI, THOP1 |
| HFE | 1.74E-06 | AMPK, BAX, BID, CASP3, ERN1, HFE, MAPK1, RB1 | ACACA, CTSS, ERAP1, GPT, IDH3A, LAMA4, MDH1, NME1, PARK7, PDHB |
| HGF | 7.62E-05 | AKT1, AKT2, AMPK, BAX, BID, CEBPA, EIF4E, EIF4EBP1, ERK1/2, GSK3B, HGF, IGF1R, IKBKB, KRAS, Mapk, MAPK1, Mek, MKNK1, MTOR, NFkB (complex), NOS2, NR3C2, P38 MAPK, PADI2, PI3K (complex), RB1, TCF7L2 | CTSS, CYCS, FAH, FBP1, GPC4, GPS1, GPT, IDH3A, LAMA4, LMNB1, MAPK14, MDH1, NME1, PARK7, PDHB, PRDX4, RAB6B |
| HIF1A | 1.13E-03 | HIF1A, JUN, RB1, TP53 | ALDH9A1, Ces1b/Ces1c, GPT, MDH1, NAE1, NME1, PARK7, TGFBI, THOP1 |
| HMGB1 | 2.45E-06 | BAX, CEBPA, EIF4E, HIF1A, HMGB1, IKBKB, INSR, JUN, MAPK14, MAPK8, MKNK1, P38 MAPK, PADI2, PPARG, RB1, RFWD2, TP53 | ACACA, ALDH9A1, Ces1b/Ces1c, CTSS, CYCS, GPC4, GPS1, GPT, IDH3A, LMNB1, NAE1, NME1, PARK7, PDHB, RAB6B, TGFBI, THOP1 |
| Hmgb1 | 2.42E-04 | BAX, BID, CASP3, CEBPA, Creb, EIF4E, HIF1A, Hmgb1, IKBKB, JUN, MMP9, NFkB (complex), PPARG, RB1 | ACACA, CTSS, CYCS, ERAP1, GPS1, GPT, LMNB1, MDH1, NAE1, NME1, PARK7 |
| HRAS | 3.94E-05 | BAX, CBX7, EIF4E, ERK, ERK1/2, HRAS, IKBKB, IRS1, Jnk, JUN, MAPK1, Mek, NOS2, NR3C2, RALA, RARA, RB1, RXRA, TCF7L2, TP53 | ACACA, ALDH9A1, Ces1b/Ces1c, CTSS, CYCS, ERAP1, FBP1, GPS1, GPT, LAMA4, LMNB1, NME1, PARK7, PDHB, TGFBI, THOP1 |
| HSPB6 | 4.08E-05 | BAX, CEBPA, EIF4E, HSPB6, IKBKB, MAP3K5, MKNK1, MTOR, NFkB (complex), P38 MAPK, PADI2, PPARG, RELA, RFWD2, TP53 | ACACA, ALDH9A1, Ces1b/Ces1c, CTSS, CYCS, ERAP1, GPC4, GPS1, LMNB1, MDH1, NME1, RAB6B, TGFBI, THOP1 |
| HSPB8 | 6.88E-05 | Akt, BAX, BID, CASP3, CEBPA, EIF4E, HSPB8, IKBKB, IRS1, KRAS, MAPK1, MAPK3, MKNK1, NFE2L2, NOS2, P38 MAPK, PADI2, PI3K (complex), PPARG, RARA, RB1, RFWD2, RPS6KB1, SREBF2 | ACACA, CTSS, CYCS, ERAP1, FAH, GBE1, GPC4, GPS1, GPT, LAMA4, LMNB1, NME1, PARK7, PRDX4, RAB6B |
| ID1 | 7.49E-05 | CDK2, CEBPA, ERK1/2, HIF1A, ID1, IKBKB, JUN, NFkB (complex), NR3C2, RB1, SMARCA4, TP53 | ALDH9A1, Ces1b/Ces1c, CYCS, ERAP1, FBP1, GPT, LMNB1, MDH1, NAE1, NME1, PARK7, PDHB, TGFBI, THOP1 |
| IFNAR1 | 5.44E-04 | CEBPA, HIF1A, IFNAR1, IKBKB, IL12 (complex), MAPK1, NFkB (complex), STAT1, STAT4, TP53 | ALDH9A1, Ces1b/Ces1c, CYCS, ERAP1, GPT, LAMA4, LMNB1, NAE1, NME1, TGFBI, THOP1 |
| IGF1 | 1.11E-03 | HIF1A, IGF1, IGF1R, IRS1, IRS2, MAPK1, RB1 | ACACA, CYCS, LAMA4, MAPK14, MDH1, NAE1, NME1, PARK7, TGFBI |
| IGF1R | 5.82E-04 | IGF1R | CYCS, MAPK14, NME1, TGFBI |
| IGHM | 1.61E-08 | AKT1, AMPK, BCR (complex), Cdc42, CRTC2, IGHM, IKBKB, INPP5D, KRAS, MKNK1, MYD88, NOS2, PI3K (complex), RB1, SYK, TICAM1, TP53 | AK3, ALDH9A1, Ces1b/Ces1c, CTSS, CYCS, FAH, GPC4, GPS1, GPT, IDH3A, LMNB1, MDH1, NME1, PARK7, PDHB, PRDX4, TGFBI, THOP1 |
| IL1 | 4.62E-06 | AMPK, BAX, CEBPA, HIF1A, IKBKB, IL1, JUN, MAP3K7, Map3k7, MAPK1, MTOR, NFkB (complex), PRKCZ, TAB1, TAB2, TP53 | ALDH9A1, Ces1b/Ces1c, CTSS, CYCS, ERAP1, GPT, IDH3A, LAMA4, LMNB1, MDH1, NAE1, NME1, PDHB, TGFBI, THOP1 |
| IL12 (complex) | 8.25E-05 | CEBPA, EIF4E, HIF1A, IKBKB, IL12 (complex), MAPK1, MAPK3, MMP9, MTOR, NFE2L2, NFkB (complex), PLAU, TGFB1, TP53 | ALDH9A1, Ces1b/Ces1c, CYCS, ERAP1, GBE1, GPS1, GPT, LAMA4, LMNB1, MDH1, NAE1, NME1, TGFBI, THOP1 |
| IL17RA | 2.70E-05 | BAX, CEBPA, CEBPB, EIF4E, IKBKB, IL17RA, INSR, MKNK1, MTOR, P38 MAPK, PADI2, PPARG, TP53 | ALDH9A1, Ces1b/Ces1c, CTSS, CYCS, GPC4, GPS1, IDH3A, LMNB1, MDH1, NME1, PDHB, RAB6B, TGFBI, THOP1 |
| IL17RD | 3.35E-04 | AKT1, AMPK, BAX, ERK, ERK1/2, IKBKB, IL17RD, MAPK1, MKNK1, NR3C2, P38 MAPK, PADI2, RXRA, TP53 | ALDH9A1, Ces1b/Ces1c, CTSS, GPC4, GPT, IDH3A, LAMA4, LMNB1, NME1, PDHB, RAB6B, TGFBI, THOP1 |
| IL1A | 1.95E-04 | BID, CASP1, CASP3, CASP8, CDK2, CEBPA, EIF4E, ERK1/2, HIF1A, HRAS, IFNG, IKBKB, IL1A, Jnk, JUN, Map3k7, MAPK1, MAPK14, MKNK1, NFE2L2, NFkB (complex), NOS2, NR3C2, P38 MAPK, PADI2, RB1, SMARCA4 | ACACA, CTSS, CYCS, ERAP1, FBP1, GBE1, GPC4, GPS1, GPT, LAMA4, LMNB1, NAE1, PARK7, PDHB, RAB6B |
| IL1B | 4.51E-05 | EIF4E, ERK, GSK3B, HIF1A, IGF1R, IKBKB, IL1B, INSR, IRS1, JAK1, JUN, KRAS, Mapk, MAPK1, MAPK12, MAPK8, MKNK1, MMP9, NFkB (complex), NOS2, OGG1, P38 MAPK, PADI2, SOD2 | ACACA, CTSS, CYCS, FAH, GPC4, GPS1, GPT, IDH3A, LAMA4, LMNB1, MAPK14, NAE1, PDHB, PRDX4, RAB6B, TGFBI, YWHAZ |
| IL1B | 2.42E-04 | IGF1R, IKBKB, IL1B, INSR, IRS1, JUN, MAPK1, NOS2, SOD2 | ACACA, CTSS, CYCS, GPT, IDH3A, LAMA4, LMNB1, MAPK14, PDHB, TGFBI, YWHAZ |
| IL1RN | 2.69E-05 | BID, CASP3, CASP7, CEBPA, HIF1A, IFNG, IKBKB, IL1RN, JUN, MAPK1, NFkB (complex), NOS2, PADI2, Pkc(s), PRKD1, PTEN, TP53 | ALDH9A1, Ces1b/Ces1c, CTSS, CYCS, ERAP1, GPT, IDH3A, LAMA4, LMNB1, MDH1, NAE1, NME1, PDHB, RAB6B, TGFBI, THOP1 |
| IL25 | 5.12E-06 | BAX, CEBPA, EIF4E, HIF1A, IKBKB, IL25, Jnk, JUN, MKNK1, MTOR, NFE2L2, NFkB (complex), P38 MAPK, PADI2, PPARG, RFWD2, TP53 | ACACA, ALDH9A1, Ces1b/Ces1c, CTSS, CYCS, ERAP1, GBE1, GPC4, GPS1, GPT, LMNB1, MDH1, NAE1, NME1, RAB6B, TGFBI, THOP1 |
| IL5 | 4.18E-02 | IL5 | AK3, PRDX4 |
| IL6 | 1.08E-07 | AKT1, AMPK, CAV1, CDK2, EIF4E, ERK, HIF1A, IGF1R, IL6, JAK1, KRAS, Mapk, MAPK1, MKNK1, MYC, NEU3, NOS2, PI3K (complex), PTPN11, RB1, SMARCA4, TP53, XRCC6 | ACACA, ALDH9A1, Ces1b/Ces1c, CYCS, ERAP1, FAH, FBP1, GPC4, GPS1, GPT, HPX, IDH3A, LAMA4, MAPK14, MDH1, NAE1, NME1, PARK7, PDHB, PRDX4, TGFBI, THOP1 |
| IL6 | 1.18E-04 | AMPK, IL6, MAPK1, MYC, NOS2, RB1 | ACACA, CYCS, ERAP1, GPT, HPX, IDH3A, LAMA4, MDH1, NME1, PARK7, PDHB |
| INHBA | 4.09E-05 | Akt, BAX, BID, CASP3, CEBPA, EIF4E, ERK1/2, GSK3B, HIF1A, IGF1R, IKBKB, INHBA, IRS1, IRS2, MKNK1, MYC, NR3C2, P38 MAPK, PADI2, PPARG, PSEN1, RB1, RFWD2, SREBF2, TP53 | ACACA, ALDH9A1, Ces1b/Ces1c, CTSS, CYCS, GPC4, GPS1, LMNB1, MAPK14, MDH1, NAE1, NME1, PARK7, PDHB, RAB6B, THOP1 |
| Ins1 | 7.54E-07 | AKT1, AMPK, BID, EIF4E, IKBKB, Ins1, KRAS, Mapk, MKNK1, NOS2, PI3K (complex), RB1, TP53 | ALDH9A1, Ces1b/Ces1c, CTSS, CYCS, FAH, GPC4, GPS1, IDH3A, LMNB1, MDH1, NME1, PARK7, PDHB, PRDX4, TGFBI, THOP1 |
| Integrin | 1.66E-06 | Cdc42, CEBPA, CTNNB1, EIF4E, ERK1/2, FYN, HIF1A, IGF1R, IKBKB, INSR, Integrin, JUN, KRAS, MKNK1, NOS2, NR3C2, P38 MAPK, PADI2, PI3K (complex), PTPN1, PTPRE, RB1, RHOA, SRC, TCF7L2, TP53 | ALDH9A1, Ces1b/Ces1c, CTSS, CYCS, FAH, FBP1, GPC4, GPS1, GPT, IDH3A, LMNB1, MAPK14, NAE1, NME1, PARK7, PDHB, PRDX4, RAB6B, THOP1 |
| Interferon alpha | 2.29E-04 | CBL, CDK2, CEBPA, EIF4E, HIF1A, IGF1R, IKBKB, Interferon alpha, Jnk, MAPK1, MKNK1, MTOR, NFE2L2, NFkB (complex), PADI2, Pkc(s), RARA, RB1, SMARCA4 | CTSS, CYCS, ERAP1, FBP1, GBE1, GPC4, GPS1, LAMA4, LMNB1, MAPK14, MDH1, NAE1, NME1, PARK7, RAB6B |
| IRAK2 | 3.93E-07 | AMPK, BAX, CEBPA, EIF4E, HIF1A, IKBKB, IRAK2, MAP2K1, MAPK1, MAPK9, MKNK1, MTOR, NFkB (complex), P38 MAPK, PADI2, PPARG, RFWD2, TP53 | ACACA, ALDH9A1, Ces1b/Ces1c, CTSS, CYCS, ERAP1, GPC4, GPS1, IDH3A, LAMA4, LMNB1, MDH1, NAE1, NME1, PDHB, RAB6B, TGFBI, THOP1 |
| JAK1 | 2.24E-04 | AKT1, EIF4E, GSK3B, HIF1A, IGF1R, IKBKB, JAK1, MAPK1, MKNK1, MTOR, MYC, PSEN1, RAF1, RARA, TP53 | ALDH9A1, Ces1b/Ces1c, CTSS, ERAP1, GPC4, GPS1, GPT, LAMA4, LMNB1, MAPK14, MDH1, NAE1, NME1, THOP1 |
| JUN | 5.95E-05 | BAX, BID, CASP3, CDK2, E2f, HIF1A, JUN, MAPK1, RB1, SMARCA4, TP53 | ALDH9A1, Ces1b/Ces1c, CTSS, ERAP1, FBP1, GPT, LAMA4, MDH1, NAE1, NME1, PARK7, TGFBI, THOP1 |
| KIT | 1.24E-05 | AKT1, AMPK, CEBPA, EIF4E, ERK1/2, HIF1A, IKBKB, INSR, JUN, KIT, MAPK1, MAPK8, MKNK1, MTOR, NR3C2, P38 MAPK, PADI2, PPARG, TP53 | ALDH9A1, Ces1b/Ces1c, CYCS, ERAP1, GPC4, GPS1, GPT, IDH3A, LAMA4, LMNB1, MDH1, NAE1, NME1, PDHB, RAB6B, TGFBI, THOP1 |
| KRAS | 5.20E-06 | Akt, ATM, BAX, CBX7, E2f, EIF4E, ERK, IKBKB, JUN, KRAS, MAPK1, Mek, NOS2, RB1, RXRA, SOD2, SREBF2, TCF7L2, TP53 | ACACA, ALDH9A1, Ces1b/Ces1c, CTSS, CYCS, FAH, FBP1, GPS1, GPT, LAMA4, LMNB1, NME1, PARK7, PRDX4, TGFBI, THOP1, YWHAZ |
| LDL | 1.14E-04 | Akt, BAX, CEBPA, CTNNB1, EIF4E, ERK, ERK1/2, ERN1, GSK3B, IGF1R, IKBKB, Jnk, KRAS, LDL, MAPK1, Mek, MKNK1, NFE2L2, NFkB (complex), NOS2, NR3C2, P38 MAPK, PADI2, PI3K (complex), RB1, SPHK1, TCF7L2 | CTSS, CYCS, ERAP1, FAH, FBP1, GBE1, GPC4, GPS1, GPT, LAMA4, LMNB1, MAPK14, PARK7, PDHB, PRDX4, RAB6B |
| LEPR | 6.61E-06 | AMPK, BAX, CEBPA, EIF4E, ERN1, IKBKB, JUN, KRAS, LEPR, MKNK1, MTOR, NCF1, NOS2, P38 MAPK, PADI2, PI3K (complex), RB1 | ACACA, CTSS, CYCS, FAH, GPC4, GPS1, GPT, IDH3A, LMNB1, MDH1, NME1, PARK7, PDHB, PRDX4, RAB6B |
| LGALS3 | 4.78E-03 | LGALS3, RB1, RELA | ERAP1, LMNB1, MDH1, NME1, PARK7 |
| LIF | 4.00E-06 | Akt, BID, EIF4E, ERK1/2, IKBKB, INSR, IRS1, KRAS, LIF, Mapk, MAPK1, MAPK3, MKNK1, NFE2L2, NOS2, NR3C2, PI3K (complex), RARA, RB1, RPE65, SREBF2 | ACACA, CTSS, CYCS, ERAP1, FAH, FBP1, GBE1, GPC4, GPS1, IDH3A, LAMA4, LMNB1, MDH1, NME1, PARK7, PDHB, PRDX4 |
| LPAR1 | 8.21E-05 | AKT1, BAX, EIF4E, ERK, HIF1A, INSR, JUN, LPAR1, MAPK1, MAPK8, MTOR, N-cor, NFkB (complex), NOS2, PLC, RAC1, RELA, RXRA, TP53 | ACACA, ALDH9A1, Ces1b/Ces1c, CTSS, CYCS, ERAP1, GPS1, GPT, LAMA4, MDH1, NAE1, NME1, TGFBI, THOP1 |
| LPAR2 | 8.25E-05 | AKT1, BAX, EIF4E, ERK, HIF1A, INSR, JUN, LPAR2, MAPK1, MAPK8, MTOR, N-cor, NFkB (complex), NOS2, PLC, RELA, RXRA, Tgf beta, TP53 | ACACA, ALDH9A1, Ces1b/Ces1c, CTSS, CYCS, ERAP1, GPS1, GPT, LAMA4, MDH1, NAE1, NME1, TGFBI, THOP1 |
| MAGI2 | 1.29E-05 | Akt, Cdc42, EIF4E, HIF1A, IGF1R, MAGI2, NFkB (complex), PTEN, RB1, RELA | ACACA, CTSS, CYCS, ERAP1, GPS1, IDH3A, MAPK14, MDH1, NAE1, NME1, PARK7, PDHB, TGFBI |
| MLIP | 3.66E-05 | Akt, Cdc42, HIF1A, IGF1R, MLIP, NFkB (complex), PRKAA, PTEN, RB1, RELA, TP53 | ACACA, ALDH9A1, Ces1b/Ces1c, CTSS, CYCS, ERAP1, IDH3A, MAPK14, MDH1, NAE1, NME1, PARK7, PDHB, THOP1 |
| MTOR | 1.05E-05 | Akt, AMPK, Gsk3, HDAC2, IGF1R, IKBKB, IRS1, MAPK1, MTOR, PDPK1, RB1, RHOA, SIRT1, Tgf beta, TP53 | ACACA, ALDH9A1, Ces1b/Ces1c, CYCS, DPT, IDH3A, LAMA4, LMNB1, MAPK14, MDH1, NME1, PARK7, PDHB, TGFBI, THOP1 |
| MYD88 | 1.39E-06 | Akt, CASP1, CEBPA, EIF4E, ERK1/2, HIF1A, IKBKB, INSR, IRAK1, IRS1, Jnk, KRAS, Mapk, MAPK8, MKNK1, MTOR, MYD88, NFkB (complex), NOS2, NR3C2, P38 MAPK, PADI2, PI3K (complex), PPARG, RB1, RFWD2, SREBF2, TP53 | ACACA, AK3, ALDH9A1, Ces1b/Ces1c, CYCS, ERAP1, FAH, GPC4, GPS1, IDH3A, LMNB1, MDH1, NAE1, PARK7, PDHB, PRDX4, RAB6B, TGFBI, THOP1 |
| NADPH oxidase | 1.15E-04 | Cdc42, CEBPA, EGFR, HDAC2, HIF1A, IKBKB, JUN, KDM5A, MAPK1, NADPH oxidase, NFkB (complex), RB1, SP1, TP53 | ACACA, ALDH9A1, Ces1b/Ces1c, CTSS, CYCS, DPT, ERAP1, GPT, LAMA4, LMNB1, MDH1, NAE1, PARK7, THOP1 |
| NCF1 | 3.10E-05 | ATM, CEBPA, EIF4E, ERK, FYN, HIF1A, IKBKB, JUN, MAP3K5, MAPK1, MTOR, NCF1, NFkB (complex), NOS2, SOD2, TP53 | ACACA, ALDH9A1, Ces1b/Ces1c, CYCS, ERAP1, GPS1, GPT, LAMA4, LMNB1, MDH1, NAE1, NME1, TGFBI, THOP1, YWHAZ |
| NFE2L2 | 4.44E-05 | 26s Proteasome, BAX, BID, CASP3, CEBPA, Creb, EIF4E, HMOX1, IKBKB, MAPK1, MKNK1, MTOR, NFE2L2, NFKB1, P38 MAPK, PADI2, Pkc(s), PPARG, RELA, TP53 | ALDH9A1, Ces1b/Ces1c, CTSS, CYCS, ERAP1, GBE1, GPC4, GPS1, GPT, LAMA4, LMNB1, MDH1, NME1, RAB6B, TGFBI, THOP1 |
| NFkB (complex) | 1.04E-06 | CEBPA, EIF4E, HIF1A, IGF1R, IKBKB, IL6ST, INSR, MAPK8, NFkB (complex), RB1, SOD2, TP53 | ACACA, ALDH9A1, Ces1b/Ces1c, CYCS, ERAP1, GPS1, HPX, IDH3A, LMNB1, MAPK14, NAE1, NME1, PARK7, PDHB, TGFBI, THOP1, YWHAZ |
| NFkB (complex) | 6.63E-04 | CEBPA, HIF1A, IKBKB, NFkB (complex), TP53 | ACACA, ALDH9A1, Ces1b/Ces1c, CYCS, ERAP1, LMNB1, NAE1, NME1, TGFBI, THOP1 |
| NOS1 | 1.31E-04 | BAX, ERK1/2, HIF1A, INSR, IRS1, JUN, MAPK8, NFkB (complex), NOS1, NR3C2, PRKAA, RELA, TP53 | ACACA, ALDH9A1, Ces1b/Ces1c, CTSS, CYCS, ERAP1, GPT, IDH3A, NAE1, NME1, PDHB, TGFBI, THOP1 |
| NPR1 | 1.72E-04 | Calcineurin protein(s), CaMKII, Cdc42, CEBPA, ERK1/2, HIF1A, IKBKB, IRS1, MYD88, NFkB (complex), NPR1, NR3C2, TP53 | ACACA, AK3, ALDH9A1, Ces1b/Ces1c, CTSS, CYCS, ERAP1, LMNB1, NAE1, NME1, PDHB, TGFBI, THOP1 |
| NR3C2 | 2.26E-04 | BAX, BID, CASP3, Creb, NR3C2, PPARG, RELA, TP53 | ACACA, ALDH9A1, Ces1b/Ces1c, CTSS, ERAP1, GPT, MDH1, NME1, PDHB, TGFBI, THOP1 |
| NTRK1 | 1.36E-04 | Akt, BID, CEBPA, EIF4E, IKBKB, INSR, IRS1, MAPK1, MAPK3, MKNK1, NFE2L2, NTRK1, P38 MAPK, PADI2, PPARG, RB1, RFWD2, SH2B1, SOD2, SREBF2 | ACACA, ERAP1, GBE1, GPC4, GPS1, GPT, IDH3A, LAMA4, LMNB1, NME1, PARK7, PDHB, RAB6B, YWHAZ |
| P38 MAPK | 1.59E-04 | ADAM17, BAX, CEBPA, CEBPB, CEBPD, EIF4E, HIF1A, IGF1R, IKBKB, IL6ST, INSR, MKNK1, MTOR, NFE2L2, NFkB (complex), P38 MAPK, PADI2, PPARG, RELA, RFWD2 | ACACA, CTSS, CYCS, ERAP1, GBE1, GPC4, GPS1, HPX, IDH3A, LMNB1, MAPK14, MDH1, NAE1, PDHB, RAB6B |
| PBK | 8.77E-06 | CEBPA, DUSP1, EIF4E, H2AFX, IKBKB, INSR, MAPK1, MAPK14, MAPK8, MKNK1, P38 MAPK, PADI2, PBK, PPARG, RB1, RFWD2, TP53 | ACACA, ALDH9A1, Ces1b/Ces1c, CYCS, GPC4, GPS1, IDH3A, LAMA4, LMNB1, NME1, PARK7, PDHB, RAB6B, TGFBI, THOP1 |
| Pdgf (complex) | 1.53E-04 | AKT1, AMPK, IKBKB, KRAS, NOS2, Pdgf (complex), PI3K (complex), RB1, TP53 | ALDH9A1, Ces1b/Ces1c, CYCS, FAH, IDH3A, LMNB1, NME1, PARK7, PDHB, PRDX4, TGFBI, THOP1 |
| PDGF BB | 2.04E-05 | CEBPA, EIF4E, ERK, ERK1/2, FOXO3, HIF1A, IKBKB, INSR, IRS1, ITGB1, Jnk, Mapk, MAPK1, MAPK8, Mek, MKNK1, NOS2, NR3C2, P38 MAPK, PADI2, PDGF BB, RAF1, RB1, TCF7L2, TP53 | ACACA, ALDH9A1, Ces1b/Ces1c, CYCS, ERAP1, FBP1, GPC4, GPS1, IDH3A, LAMA4, LMNB1, MDH1, NAE1, NME1, PARK7, PDHB, RAB6B, THOP1 |
| PF4 | 5.87E-05 | BAX, CEBPA, EIF4E, ERK1/2, IKBKB, IRS1, Jnk, JUN, KRAS, MAPK1, MKNK1, NFE2L2, NOS2, NR3C2, P38 MAPK, PADI2, PF4, PI3K (complex), PPARG, RB1, RFWD2 | ACACA, CTSS, CYCS, FAH, GBE1, GPC4, GPS1, GPT, LAMA4, LMNB1, NME1, PARK7, PDHB, PRDX4, RAB6B |
| PIK3CB | 2.18E-04 | Akt, AKT1, AMPK, AURKB, GSK3A, GSK3B, HIF1A, IGF1R, MAPK1, MYC, PIK3CB, PSEN1, RB1, TP53 | ALDH9A1, Ces1b/Ces1c, CTSS, CYCS, GPT, IDH3A, LAMA4, MAPK14, NAE1, NME1, PARK7, PDHB, THOP1 |
| PLA2G10 | 6.21E-04 | Cpla2, HIF1A, INSR, JUN, MAPK1, MAPK8, NR1H3, PLA2G10, PPARG | ACACA, CYCS, ERAP1, FBP1, GPT, IDH3A, LAMA4, MDH1, NAE1, PDHB |
| PLA2G10 | 5.30E-03 | MAPK1, NR1H3, PLA2G10 | ACACA, ERAP1, FBP1, LAMA4 |
| PLAC8 | 4.34E-05 | AKT1, AMPK, IGF1R, MAPK1, MDM2, N-cor, NFkB (complex), PLAC8, RB1, TP53 | ACACA, ALDH9A1, Ces1b/Ces1c, CYCS, ERAP1, IDH3A, LAMA4, MAPK14, MDH1, NME1, PARK7, PDHB, THOP1 |
| PLAT | 3.05E-04 | BAX, BID, CASP1, CASP3, CASP8, CEBPA, EIF4E, HIF1A, IKBKB, IRS1, JUN, MKNK1, MTOR, NFkB (complex), P38 MAPK, PADI2, PLAT, PPARG, PRKACA, RB1, RELA, RFWD2 | ACACA, CTSS, CYCS, ERAP1, GPC4, GPS1, GPT, LMNB1, MDH1, NAE1, NME1, PARK7, RAB6B |
| PPARA | 2.95E-02 | PPARA | ACACA, GPT, HPX |
| PPARD | 3.33E-02 | PPARD | ALDH9A1, FBP1 |
| PPARG | 4.49E-03 | PPARG | ACACA, GPT, MDH1, PDHB |
| PPP1CB | 8.14E-05 | AKT1, AMPK, CEBPA, HIF1A, IKBKB, MAPK1, N-cor, NFkB (complex), PPP1CB, TP53 | ACACA, ALDH9A1, Ces1b/Ces1c, CYCS, ERAP1, IDH3A, LAMA4, LMNB1, NAE1, NME1, PDHB, TGFBI, THOP1 |
| PRDX2 | 9.27E-05 | BAX, BTK, CEBPA, CTNNB1, EIF4E, ERK, ERK1/2, HIF1A, IGF1R, IKBKB, MAPK1, MKNK1, NFkB (complex), NOS2, NR3C2, P38 MAPK, PADI2, PRDX2, PTPN11, RB1, SRC, TCF7L2, TP53 | ALDH9A1, Ces1b/Ces1c, CTSS, CYCS, ERAP1, FBP1, GPC4, GPS1, LAMA4, LMNB1, MAPK14, NAE1, PARK7, PDHB, RAB6B, TGFBI, THOP1 |
| PROC | 6.62E-06 | BID, CASP1, CASP3, CASP8, CEBPA, EGFR, EIF4E, HDAC6, HIF1A, IKBKB, JUN, MAPK1, MKNK1, MTOR, NFkB (complex), P38 MAPK, PADI2, PPARG, PROC, RB1, RFWD2, TP53 | ACACA, ALDH9A1, Ces1b/Ces1c, CYCS, ERAP1, GPC4, GPS1, GPT, LAMA4, LMNB1, MDH1, NAE1, NME1, PARK7, RAB6B, TGFBI, THOP1 |
| PSEN1 | 4.86E-04 | PSEN1 | CTSS, CYCS, NME1, PEBP1, YWHAZ |
| PTEN | 4.93E-05 | PTEN | ACACA, IDH3A, MAPK14, MDH1, PDHB |
| REN | 1.27E-04 | CEBPA, EIF4E, ERK, HIF1A, IKBKB, MTOR, NFkB (complex), NOS2, REN, RXRA, TP53 | ACACA, ALDH9A1, Ces1b/Ces1c, CYCS, ERAP1, GPS1, GPT, LMNB1, MDH1, NAE1, NME1, TGFBI, THOP1 |
| Ren2 | 1.98E-02 | Ren2 | CYCS |
| SELE | 3.28E-05 | BAX, CEBPA, EIF4E, ERK, Hsp27, IKBKB, MAPK1, MKNK1, MTOR, NOS2, P38 MAPK, PADI2, PPARG, RFWD2, RXRA, SELE, TP53 | ACACA, ALDH9A1, Ces1b/Ces1c, CTSS, CYCS, GPC4, GPS1, GPT, LAMA4, LMNB1, MDH1, NME1, RAB6B, TGFBI, THOP1 |
| SEMA7A | 3.92E-02 | SEMA7A | CTSS |
| SERPINA1 | 5.51E-04 | AKT1, AMPK, EIF4E, ERK1/2, IGF1R, IKBKB, IRS1, JUN, MMP9, MTOR, MYC, NR3C2, RB1, SERPINA1, SPHK1 | ACACA, CYCS, GPS1, GPT, IDH3A, LMNB1, MAPK14, MDH1, PARK7, PDHB, TGFBI |
| SLC2A4 | 2.40E-05 | IGF1R, IKBKB, INSR, IRS1, KRAS, PI3K (complex), RB1, SLC2A4 | ACACA, CYCS, FAH, IDH3A, LMNB1, MAPK14, MDH1, NME1, PARK7, PDHB, PRDX4, TGFBI |
| SLC2A4 | 2.00E-03 | INSR, IRS1, SLC2A4 | ACACA, CYCS, IDH3A, PDHB |
| SLIT2 | 1.52E-04 | AKT1, AMPK, GSK3B, HIF1A, IGF1R, MAPK1, MTOR, MYC, PSEN1, SLIT2, TP53 | ALDH9A1, Ces1b/Ces1c, CTSS, CYCS, GPT, IDH3A, LAMA4, MAPK14, MDH1, NAE1, NME1, PDHB, THOP1 |
| SOCS1 | 1.78E-04 | BAX, CEBPA, EIF4E, HIF1A, IKBKB, INSR, IRS2, Jnk, JUN, MAPK1, MAPK8, MKNK1, MTOR, NFKBIA, P38 MAPK, PADI2, PPARG, SOCS1, STAT1 | ACACA, CTSS, CYCS, GPC4, GPS1, GPT, IDH3A, LAMA4, LMNB1, MDH1, NAE1, PDHB, RAB6B |
| SOD1 | 6.60E-06 | BID, Calcineurin protein(s), CEBPA, HIF1A, IKBKB, MYD88, NFkB (complex), SOD1, SOD2, TP53 | ACACA, AK3, ALDH9A1, Ces1b/Ces1c, CTSS, CYCS, ERAP1, GPT, LMNB1, NAE1, NME1, TGFBI, THOP1, YWHAZ |
| SOD2 | 3.12E-05 | BID, CEBPA, HIF1A, IKBKB, Jnk, NFE2L2, NFkB (complex), SOD2, TP53 | ACACA, ALDH9A1, Ces1b/Ces1c, CTSS, CYCS, ERAP1, GBE1, GPT, LMNB1, NAE1, NME1, TGFBI, THOP1, YWHAZ |
| STK25 | 9.28E-03 | BAX, STK25, YWHAZ | CTSS, FBP1 |
| STK3 | 1.54E-04 | AKT1, BAX, HIF1A, IKBKB, INSR, Jnk, JUN, MAPK1, MAPK8, N-cor, NFE2L2, RAF1, RHOA, STK3, Tgf beta, TP53 | ACACA, ALDH9A1, Ces1b/Ces1c, CTSS, CYCS, GBE1, GPT, LAMA4, LMNB1, NAE1, NME1, TGFBI, THOP1 |
| TERT | 1.16E-04 | ATM, EIF4E, EIF4EBP1, IKBKB, JUN, MAPK1, NFkB (complex), RB1, RELA, SOD2, TERT | ERAP1, GPS1, GPT, LAMA4, LMNB1, MDH1, NME1, PARK7, YWHAZ |
| TNFRSF1A | 2.90E-04 | CASP1, CASP8, CD44, CEBPA, EIF4E, ERK1/2, Gsk3, IGF1R, IKBKB, INSR, IRS1, Jnk, MAPK1, MAPK14, MAPK8, MAPK9, MKNK1, NFkB (complex), NFKB1, P38 MAPK, PADI2, PPARG, PRKCD, RB1, RELA, RFWD2, TNFRSF1A, TP53 | ACACA, ALDH9A1, Ces1b/Ces1c, ERAP1, GPC4, GPS1, IDH3A, LAMA4, LMNB1, MAPK14, NME1, PARK7, RAB6B, THOP1 |
| TNFRSF1B | 1.96E-06 | BAX, BID, CASP1, CASP3, CASP8, CEBPA, EIF4E, ERK1/2, HIF1A, IKBKB, IRS1, Jnk, JUN, MKNK1, MTOR, NFE2L2, NFkB (complex), NR3C2, P38 MAPK, PADI2, PPARG, RELA, RFWD2, TNFRSF1B, TP53 | ACACA, ALDH9A1, Ces1b/Ces1c, CTSS, CYCS, ERAP1, GBE1, GPC4, GPS1, GPT, LMNB1, MDH1, NAE1, NME1, PDHB, RAB6B, TGFBI, THOP1 |
| TNFRSF25 | 5.20E-06 | BAX, CEBPA, EIF4E, ERK, HIF1A, IKBKB, MAPK1, MKNK1, MTOR, NFkB (complex), NFKB1, NOS2, P38 MAPK, PADI2, PPARG, RFWD2, RXRA, TNFRSF25, TP53 | ACACA, ALDH9A1, Ces1b/Ces1c, CTSS, CYCS, ERAP1, GPC4, GPS1, GPT, LAMA4, LMNB1, MDH1, NAE1, NME1, RAB6B, TGFBI, THOP1 |
| TNFSF14 | 1.20E-05 | BAX, BID, CASP3, CEBPA, HIF1A, IKBKB, INSR, JUN, MAPK8, NFkB (complex), TNFSF14, TP53 | ACACA, ALDH9A1, Ces1b/Ces1c, CTSS, CYCS, ERAP1, GPT, IDH3A, LMNB1, NAE1, NME1, PDHB, TGFBI, THOP1 |
| TNFSF15 | 9.20E-07 | BAX, CEBPA, EIF4E, ERK1/2, HIF1A, IKBKB, IRS1, Jnk, JUN, MAPK1, MKNK1, MTOR, NFE2L2, NFkB (complex), NFKB1, NR3C2, P38 MAPK, PADI2, PPARG, RFWD2, RIPK1, TNFSF15, TP53 | ACACA, ALDH9A1, Ces1b/Ces1c, CTSS, CYCS, ERAP1, GBE1, GPC4, GPS1, GPT, LAMA4, LMNB1, MDH1, NAE1, NME1, PDHB, RAB6B, TGFBI, THOP1 |
| TP53 | 1.61E-05 | BAX, EIF4E, IGF1R, JUN, MAPK9, MTOR, NFkB (complex), SOD2, TP53 | ACACA, ALDH9A1, Ces1b/Ces1c, CTSS, CYCS, ERAP1, GPS1, GPT, MAPK14, MDH1, NME1, TGFBI, THOP1, YWHAZ |
| TRAF3IP2 | 1.71E-04 | CEBPA, HIF1A, IKBKB, JUN, MAPK7, NFkB (complex), PPARG, TP53, TRAF3IP2 | ACACA, ALDH9A1, Ces1b/Ces1c, CYCS, ERAP1, GPT, LMNB1, MDH1, NAE1, NME1, TGFBI, THOP1 |
| VDR | 4.90E-05 | CYP19A1, ESR1, FOXO3, HIF1A, IKBKB, IRS1, KRAS, NOS2, PI3K (complex), RB1, TP53, VDR | ACACA, ALDH9A1, Ces1b/Ces1c, CTSS, CYCS, FAH, LMNB1, MDH1, NAE1, NME1, PARK7, PRDX4, TGFBI, THOP1 |
| WRN | 2.47E-04 | ESR1, IKBKB, INSR, KRAS, MAPK1, NFE2L2, PRKCB, PRKCD, PRKCE, RB1, WRN | CTSS, CYCS, FAH, GBE1, IDH3A, LAMA4, LMNB1, MDH1, NME1, PARK7, PDHB, PRDX4 |

Proteins with correlation to DNL-ratio were subjected to IPA Core analyses. Upstrean regulating molecules or master regulators of signalling networks involved in fibrosis were screened for the key word „fibrosis“ (Ingenuity, Qiagen) are indicated.
