# Supplementary figures and images for: Adipokinome Signatures in Obese Mouse Models Reflect Adipose Tissue Health and Are Associated with Serum Lipid Composition
Source: Int J Mol Sci. 2019 May 24;20(10):2559. doi: 10.3390/ijms20102559 (PMC6567124; doi:10.3390/ijms20102559)

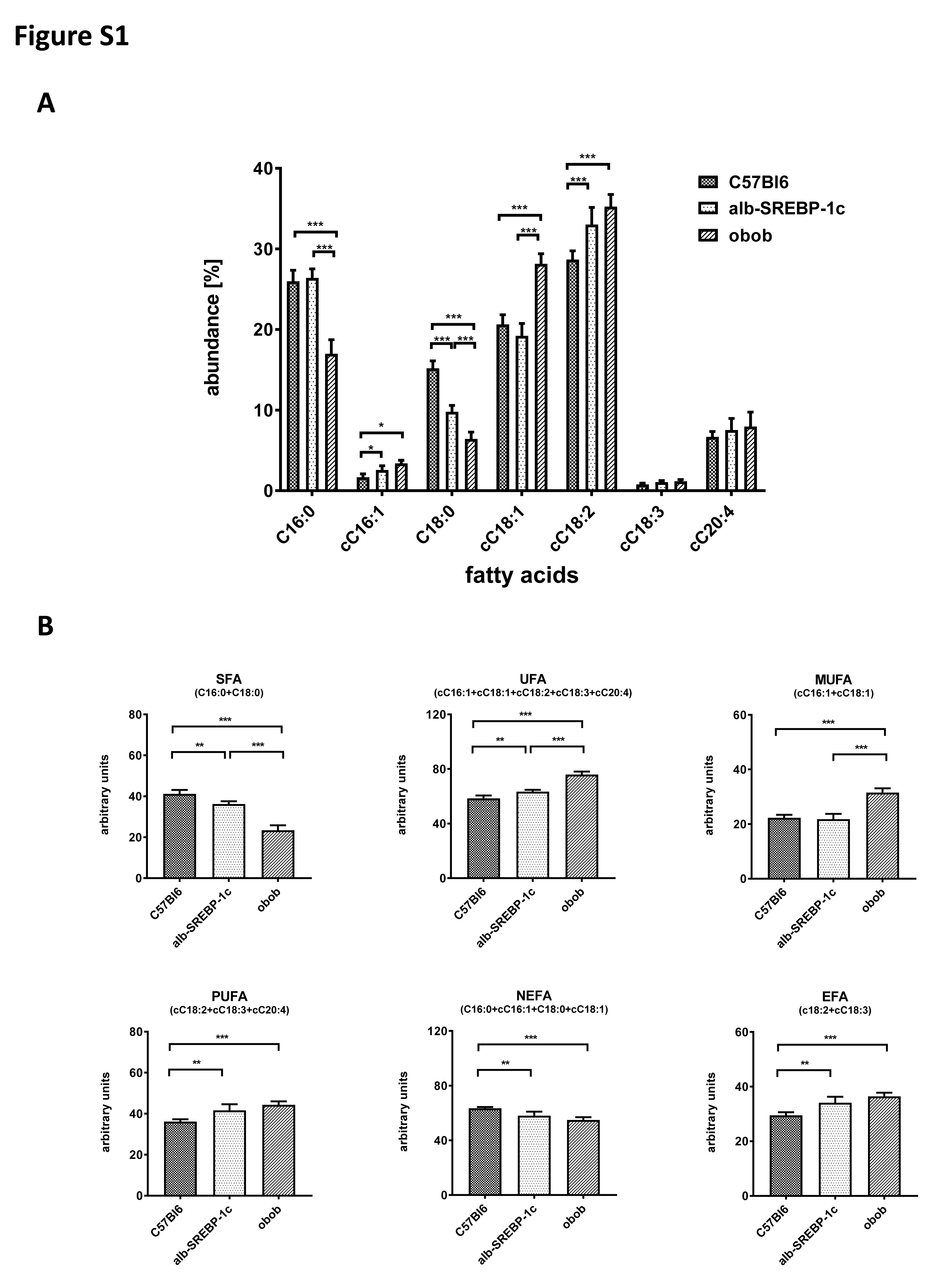

Supplement: Supplementary file 1 [file ijms-20-02559-s001.zip › IJMS_Figure S1.tif]

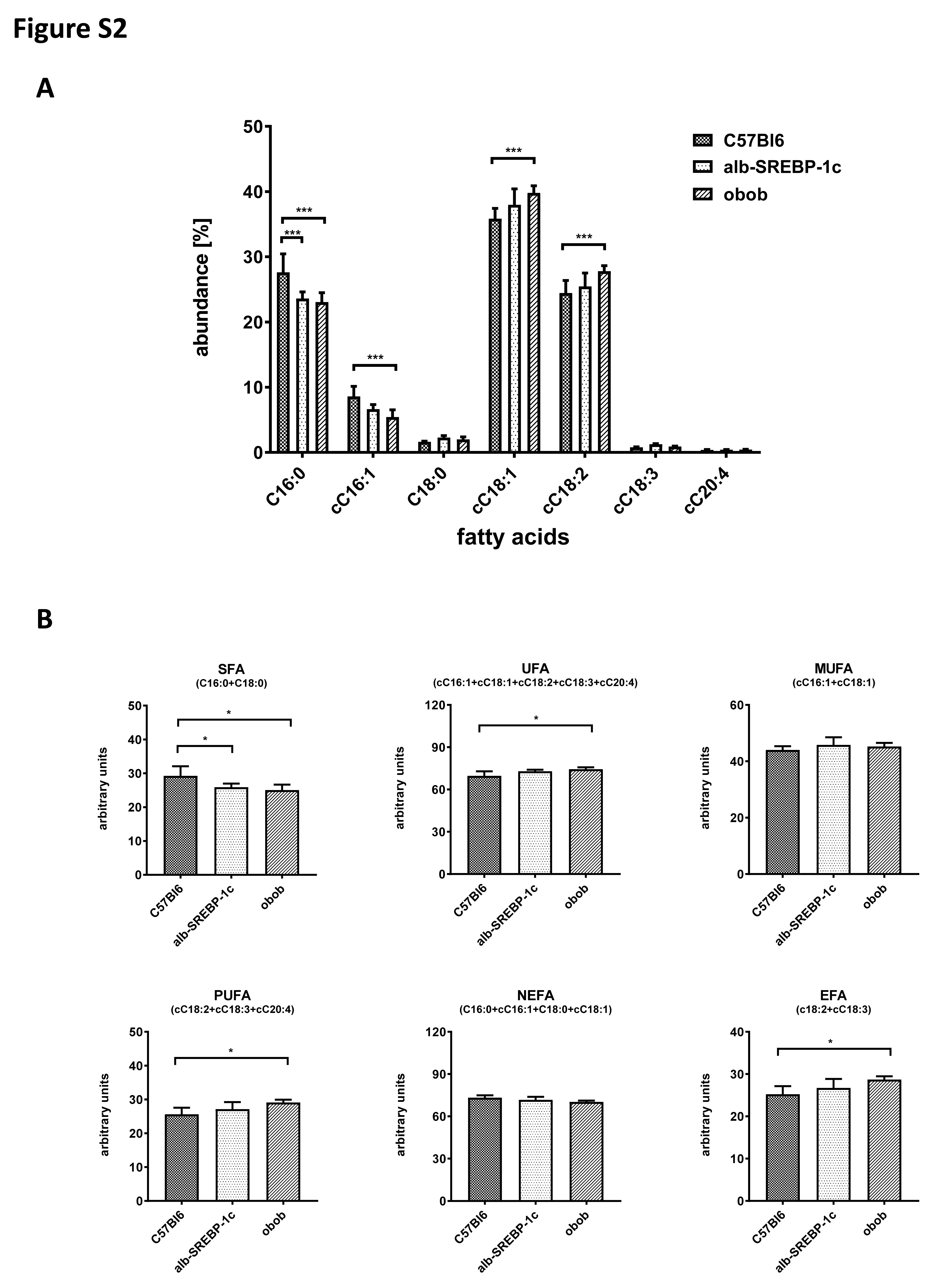

Supplement: Supplementary file 1 [file ijms-20-02559-s001.zip › IJMS_Figure S2.tif]

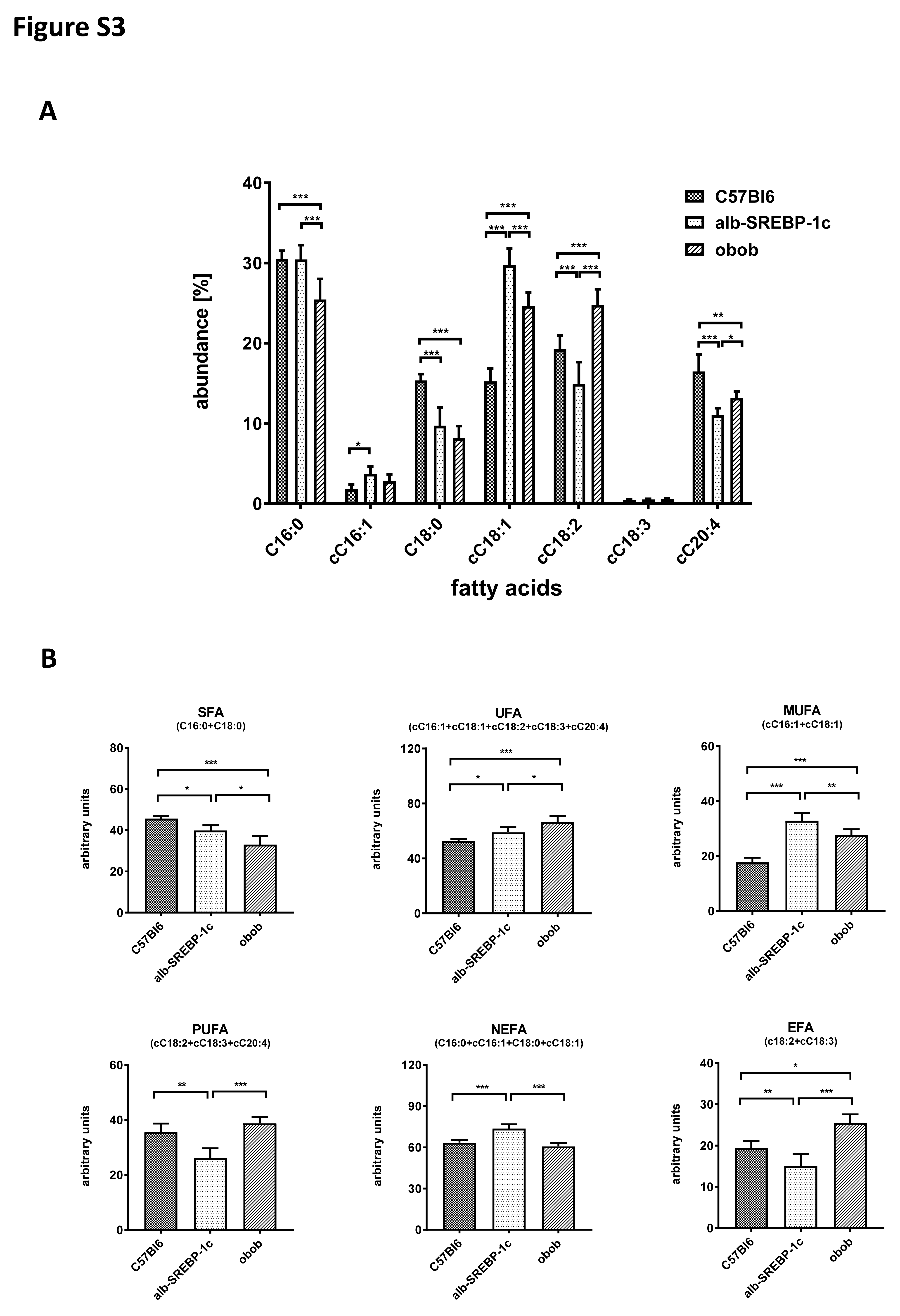

Supplement: Supplementary file 1 [file ijms-20-02559-s001.zip › IJMS_Figure S3.tif]

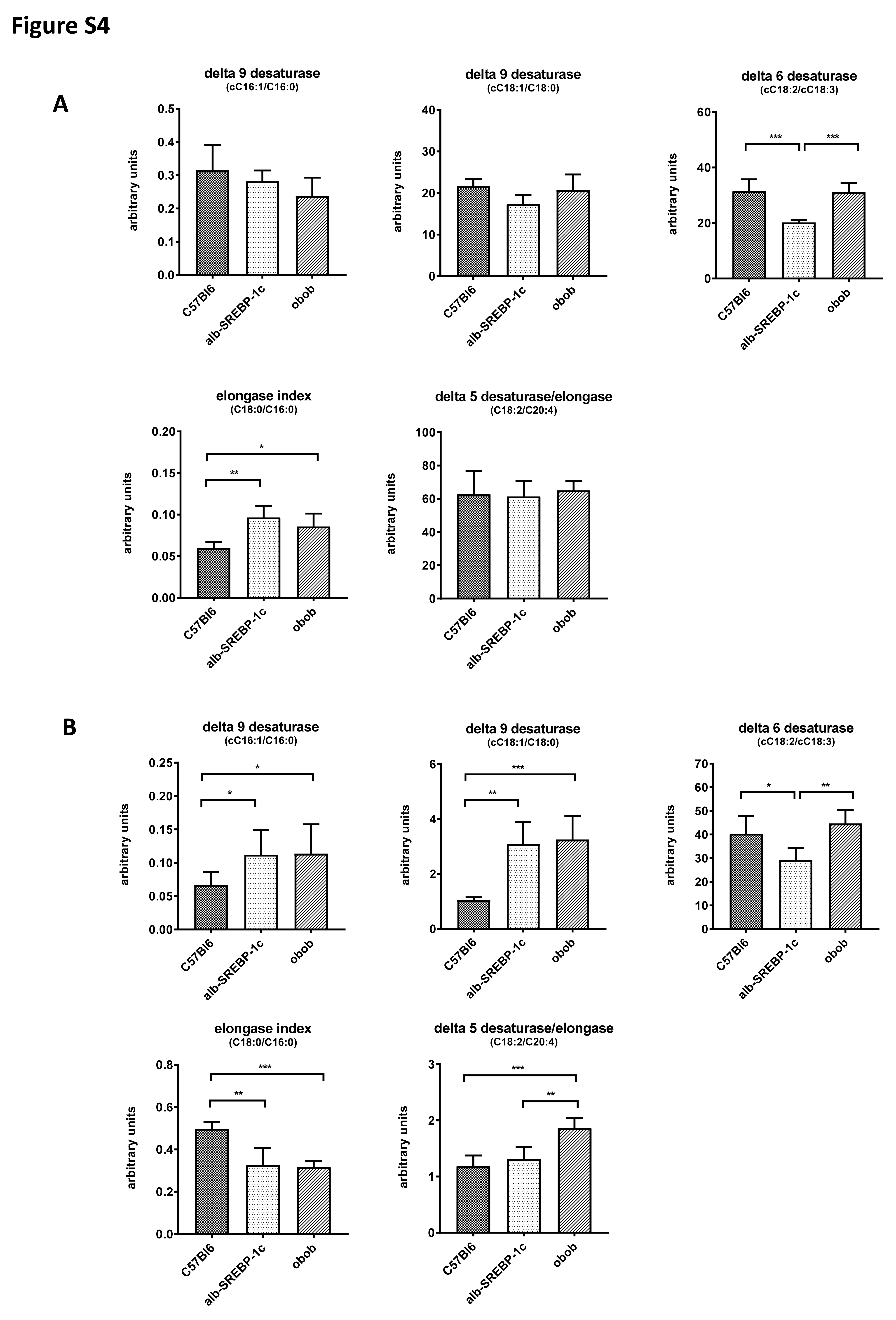

Supplement: Supplementary file 1 [file ijms-20-02559-s001.zip › IJMS_Figure S4.tif]
