# Supplementary material for: Adipokinome Signatures in Obese Mouse Models Reflect Adipose Tissue Health and Are Associated with Serum Lipid Composition
Source: Int J Mol Sci. 2019 May 24;20(10):2559. doi: 10.3390/ijms20102559 (PMC6567124; doi:10.3390/ijms20102559)
Supplement: Supplementary file 1 [file ijms-20-02559-s001.zip › revision IJMS Online Supplement Data.doc]

**Online Supplement Data**

**Supplement Figure S1** Division of serum lipids in chemical groups

Data are expressed as mean ± SD (n = 15 of each phenotype). *p < 0.05, **p < 0.01 by 1-way ANOVA with Sidak post-hoc test.

**Supplement Figure S2** Division of adipose tissue lipids in chemical groups

Data are expressed as mean ± SD (n = 15 of each phenotype). *p < 0.05, **p < 0.01 by 1-way ANOVA with Sidak post-hoc test.

**Supplement Figure S2** Division of liver tissue lipids in chemical groups

Data are expressed as mean ± SD (n = 15 of each phenotype). *p < 0.05, **p < 0.01 by 1-way ANOVA with Sidak post-hoc test.

**Supplement Figure S4. Indices for lipid metabolism of C57, alb-SREBP-1c and obob mice in WAT (A) and liver (B).**

Elongation index (C18:0/C16:0), ∆9 desaturase index (cC16:1/C16:0), ∆9 desaturase index (cC18:1/C18:0), ∆6 desaturase index (cC18:3/cC18:2), ∆5 desaturase index (cC18:2/cC20:4) derived from serum FFA, liver TFA or adipose tissue TFA measures.

Data are expressed as mean ± SD (n = 15 of each phenotype).

*p < 0.05, **p < 0.01 by 1-way ANOVA with Sidak post-hoc test.

**Supplemental Table 1 Pearson correlation analyses of liver-to-adipose-tissue DNL-ratio and adipokines**

**Supplemental Table 2 Identified Proteins from secretomes of visceral adipocytes of male mice**

**Supplemental Table 3 Differential abundance of adipokines**

**Supplemental Table 4 Enrichment analyses of adipokines**

**Supplemental Table 5 Accompanying data to Figure 4: Enrichment analyses of phenotype predictive differential abundant adipokines**

**Supplemental Table 6 Accompanying data to Figure 6, IPA Core analyses of adipokines with correlation liver-to-adipose-tissue DNL ratio with focus on cC18:1 (A) or fibrosis (B) related gene sets**.
